# Supplementary material for: The double protonation of dihapto-coordinated benzene complexes enables dearomatization using aromatic nucleophiles
Source: Nat Commun. 2023 May 30;14:3145. doi: 10.1038/s41467-023-38945-0 (PMC10229636; doi:10.1038/s41467-023-38945-0)
Supplement: Supplementary file 1 — Supplementary Information [file 41467_2023_38945_MOESM1_ESM.pdf]

The Double Protonation of Dihapto-Coordinated Benzene Complexes Enables Dearomatization Using Aromatic Nucleophiles

Justin T. Weatherford-Pratt, Jacob A. Smith, Jeremy M. Bloch, Megan N. Ericson, Jeffery T. Myers, Karl S. Westendorff, Diane A. Dickie, and W. Dean Harman*

Department of Chemistry, University of Virginia, Charlottesville, Virginia 22904, United States

Supplementary Information

Table of Contents

Supplementary Fig 1. Compound 5 ^{1}H NMR (800 MHz, CD_2Cl_2, δ, 0 °C) and ^{13}C NMR (800 MHz, CD_2Cl_2, δ, 0 °C) 4

Supplementary Fig 2. Compound 14P ^{1}H NMR (800 MHz, *d*$_3$-MeCN, δ, 0 °C) and ^{13}C NMR (800 MHz, *d*$_3$-MeCN, δ, 0 °C) 5

Supplementary Fig 3. Compound 14D ^{1}H NMR (600 MHz, *d*$_3$-MeCN, δ, 25 °C) and ^{13}C NMR (800 MHz, *d*$_3$-MeCN, δ, 0 °C) 6

Supplementary Fig 4. Compound 15D ^{1}H NMR (800 MHz, *d*$_6$-acetone, δ, 25 °C) and ^{13}C NMR (800 MHz, *d*$_6$-acetone, δ, 25 °C) 7

Supplementary Fig 5. Compound 16D ^{1}H NMR (800 MHz, *d*$_6$-acetone, δ, 25 °C) and ^{13}C NMR (800 MHz, *d*$_6$-acetone, δ, 25 °C) 8

Supplementary Fig 6. Compound 17D^{1}H NMR (800 MHz, *d*$_6$-acetone, δ, 25 °C) and ^{13}C NMR (800 MHz, *d*$_6$-acetone, δ, 25 °C) 9

Supplementary Fig 7. Compound 17P ^{1}H NMR (600 MHz, *d*$_6$-acetone, δ, 25 °C) and ^{13}C NMR (800 MHz, *d*$_6$-acetone, δ, 25 °C) 10

Supplementary Fig 8. Compound 6D ^{1}H NMR (800 MHz, *d*$_3$-MeCN, δ, 25 °C) and ^{13}C NMR (800 MHz, *d*$_3$-MeCN, δ, 25 °C) 11

Supplementary Fig 9. Compound 7D ^{1}H NMR (800 MHz, *d*$_3$-MeCN, δ, 25 °C) and ^{13}C NMR (800 MHz, *d*$_3$-MeCN, δ, 25 °C) 12

Supplementary Fig 10. Compound 8D ^{1}H NMR (600 MHz, *d*$_3$-MeCN, δ, 25 °C) and ^{13}C NMR (800 MHz, *d*$_3$-MeCN, δ, 25 °C) 13

Supplementary Fig 11. Compound 8P ^{1}H NMR (600 MHz, *d*$_3$-MeCN, δ, 25 °C) and ^{13}C NMR (800 MHz, *d*$_3$-MeCN, δ, 25 °C) 14

Supplementary Fig 12. Compound 9D ^{1}H NMR (600 MHz, *d*$_3$-MeCN, δ, 25 °C) and ^{13}C NMR (800 MHz, *d*$_3$-MeCN, δ, 25 °C) 15

Supplementary Fig 13. Compound 10D ^{1}H NMR (600 MHz, *d*$_6$-acetone, δ, 25 °C) and ^{13}C NMR (800 MHz, *d*$_6$-acetone, δ, 25 °C) 16

Supplementary Fig 14. Compound 11D ^{1}H NMR (800 MHz, *d*$_6$-acetone, δ, 25 °C) and ^{13}C NMR (800 MHz, *d*$_6$-acetone, δ, 25 °C) 17

Supplementary Fig 15. Compound 11P(a) and 11P(b) ^{1}H NMR (800 MHz, CD_2Cl_2, δ, 25 °C) and NOESY (800 MHz, CD_2Cl_2, δ, 25 °C) 18

Supplementary Fig 16. Compound 21 ^{1}H NMR (800 MHz, *d*$_6$-acetone, δ, 25 °C) and ^{13}C NMR (800 MHz, *d*$_6$-acetone, δ, 25 °C) 19

Supplementary Fig 17. Compound 22 ^{1}H NMR (600 MHz, *d*$_3$-MeCN, δ, 25 °C) and ^{13}C NMR (800 MHz, *d*$_3$-MeCN, δ, 25 °C) 20

Supplementary Fig 18. Compound 23 ^{1}H NMR (600 MHz, *d*$_6$-acetone, δ, 25 °C) and ^{13}C NMR (800 MHz, *d*$_6$-acetone, δ, 25 °C) 21

Supplementary Fig 19. Compound 6D-*d*$_6$ ^{1}H NMR (800 MHz, *d*$_3$-MeCN, δ, 25 °C) 22

Supplementary Fig 20. Compound 6D-Mo ^{1}H NMR (800 MHz, *d*$_6$-acetone, δ, 25 °C) 22

Supplementary Fig 21. Compound 24D ^{1}H NMR (800 MHz, CD_2Cl_2, δ, 25 °C) and ^{13}C NMR (800 MHz, CD_2Cl_2, δ, 25 °C) 23

Supplementary Fig 22. Compound 25D ^{1}H NMR (800 MHz, d_3-MeCN, δ, 25 °C) and ^{31}P NMR (500 MHz, d_3-MeCN, δ, 25 °C) 24

Supplementary Fig 23. Compound 26D ^{1}H NMR (800 MHz, CD_2Cl_2, δ, 25 °C) and ^{13}C NMR (800 MHz, CD_2Cl_2, δ, 25 °C) 25

Supplementary Fig 24. Compound 27D ^{1}H NMR (800 MHz, d_3-MeCN, δ, 25 °C) and ^{13}C NMR (800 MHz, d_3-MeCN, δ, 25 °C) 26

Supplementary Fig 25. Compound 28D ^{1}H NMR (800 MHz, CD_2Cl_2, δ, 25 °C) and ^{13}C NMR (800 MHz, CD_2Cl_2, δ, 25 °C) 27

Supplementary Fig 26. Compound 29D ^{1}H NMR (600 MHz, CD_2Cl_2, δ, 25 °C) and ^{13}C NMR (800 MHz, CD_2Cl_2, δ, 25 °C) 28

Supplementary Fig 27. Compound 30D ^{1}H NMR (800 MHz, d_6-acetone, δ, 25 °C) and ^{13}C NMR (800 MHz, d_3-acetone, δ, 25 °C) 29

Supplementary Fig 28. Compound 31D ^{1}H NMR (800 MHz, CD_2Cl_2, δ, 25 °C) and ^{13}C NMR (800 MHz, CD_2Cl_2, δ, 25 °C) 30

Supplementary Fig 29. Compound 32D ^{1}H NMR (800 MHz, CD_2Cl_2, δ, 25 °C) and ^{13}C NMR (800 MHz, CD_2Cl_2, δ, 25 °C) 31

Supplementary Fig 30. Compound 33D ^{1}H NMR (800 MHz, d_3-MeCN, δ, 25 °C) and ^{13}C NMR (800 MHz, d_3-MeCN, δ, 25 °C) 32

Supplementary Fig 31. Compound 34D ^{1}H NMR (800 MHz, d_3-MeCN, δ, 25 °C) and ^{13}C NMR (800 MHz, d_3-MeCN, δ, 25 °C) 33

Supplementary Fig 32. Compound 35 ^{1}H NMR (800 MHz, d_6-DMSO, δ, 25 °C) and ^{13}C NMR (800 MHz, d_6-DMSO, δ, 25 °C) 34

Supplementary Fig 33. Compound 36 ^{1}H NMR (800 MHz, d_6-acetone, δ, 25 °C) and ^{13}C NMR (800 MHz, d_6-acetone, δ, 25 °C) 35

Supplementary Fig 34. Compound 37 ^{1}H NMR (800 MHz, CD_2Cl_2, δ, 25 °C) and ^{13}C NMR (800 MHz, CD_2Cl_2, δ, 25 °C) 36

Supplementary Fig 35. Compound 38 ^{1}H NMR (800 MHz, d_6-acetone, δ, 25 °C) and ^{13}C NMR (800 MHz, d_6-acetone, δ, 25 °C) 38

Supplementary Fig 36. Compound 39 ^{1}H NMR (800 MHz, d_6-acetone, δ, 25 °C) and ^{13}C NMR (800 MHz, d_6-acetone, δ, 25 °C) 39

Supplementary Fig 37. Compound 40 ^{1}H NMR (800 MHz, d_6-acetone, δ, 25 °C) and ^{13}C NMR (800 MHz, d_6-acetone, δ, 25 °C) 40

Supplementary Fig 38. Compound 41 ^{1}H NMR (800 MHz, d_3-MeCN, δ, 25 °C) and ^{13}C NMR (800 MHz, d_3-MeCN, δ, 25 °C) 41

Experimental Procedures & Characterizations 42

Supplementary Fig 39. DFT Calculations for 1 64

Supplementary Fig 40. DFT Calculations for 1H, 1H ---- 2, & 2 65

Supplementary Fig 41. DFT Calculations for 2H, 2H --- 5, & 5 66

Supplementary Fig 42. Treatment of 1 with neat DOTf 68

Crystallographic Data 68

References 71

Supplementary Fig 1. Compound 5 ^{1}H NMR (800 MHz, CD_2Cl_2, δ, 0 °C) and ^{13}C NMR (800 MHz, CD_2Cl_2, δ, 0 °C)

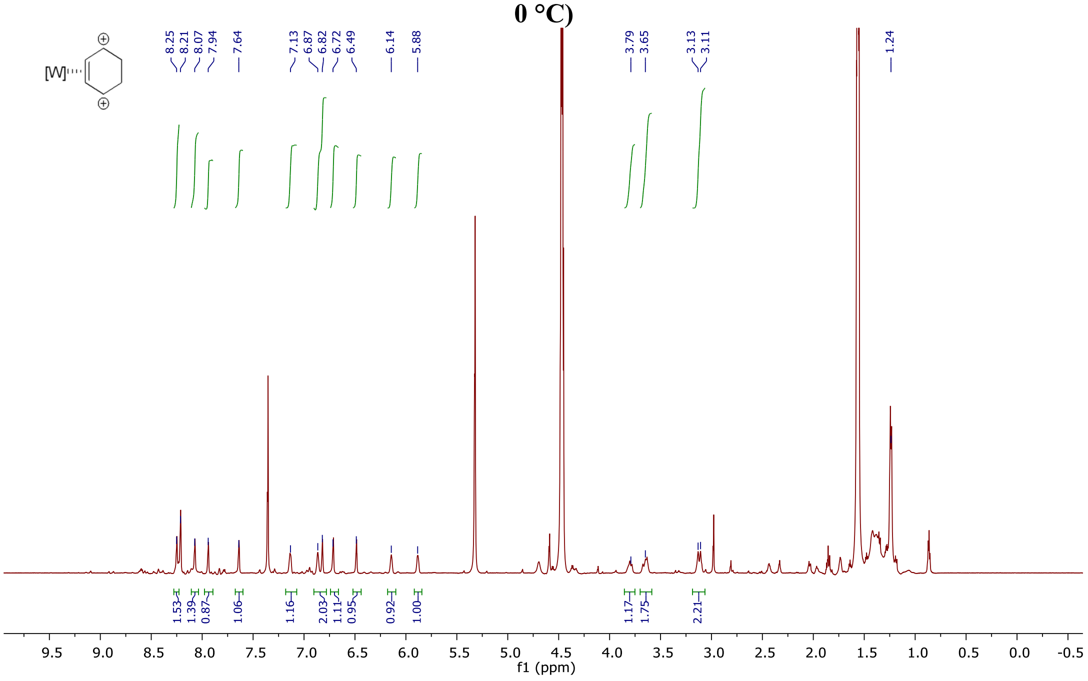

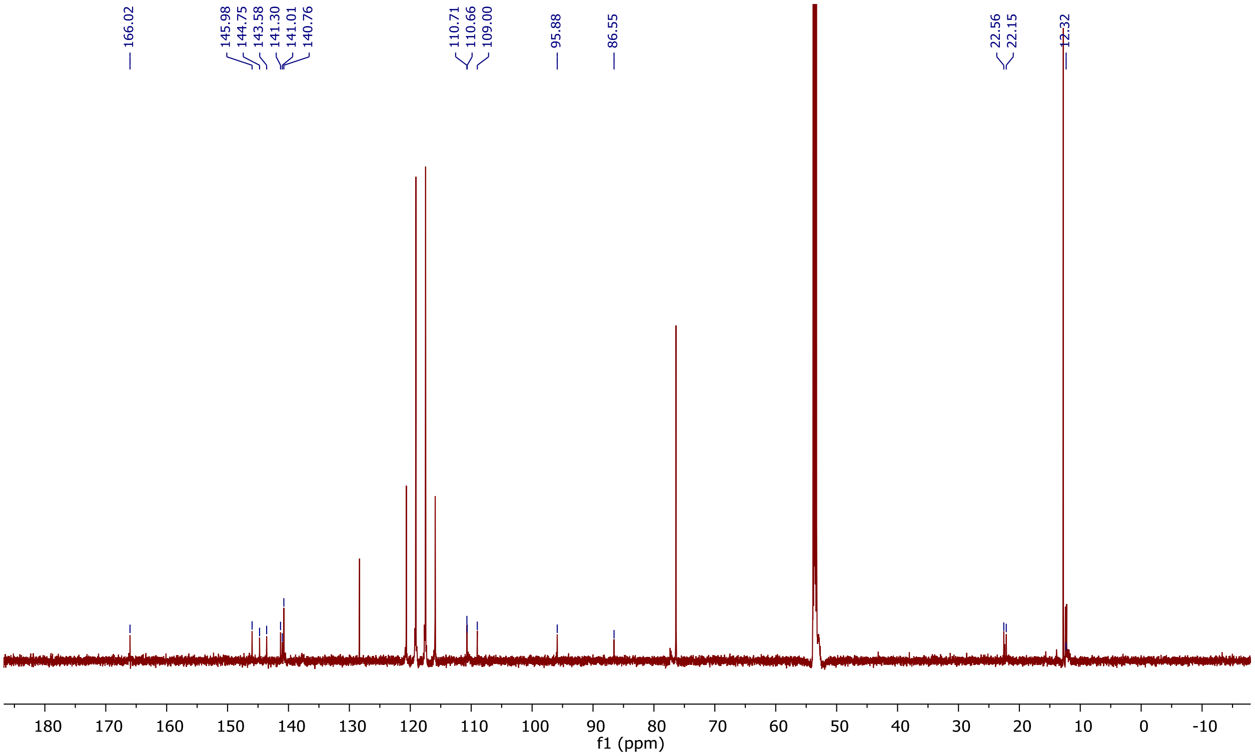

Supplementary Fig 2. Compound 14P ^{1}H NMR (800 MHz, d_3-MeCN, δ, 0 °C) and ^{13}C NMR (800 MHz, d_3-MeCN, δ, 0 °C)

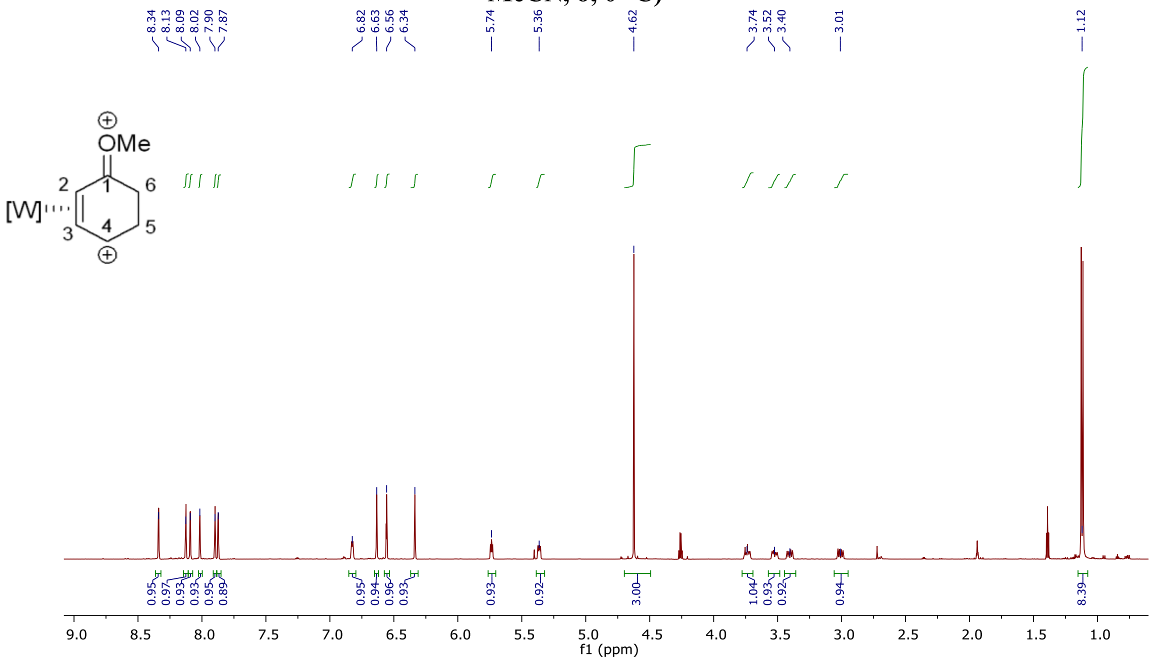

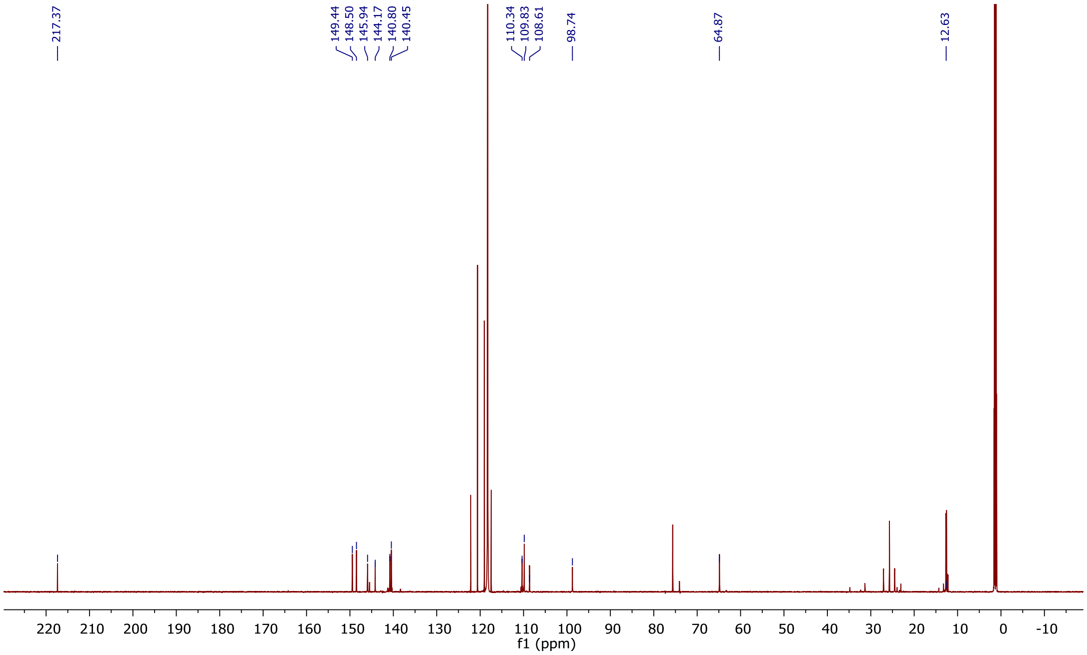

Supplementary Fig 3. Compound 14D ^{1}H NMR (600 MHz, d_3-MeCN, δ, 25 °C) and ^{13}C NMR (800 MHz, d_3-MeCN, δ, 0 °C)

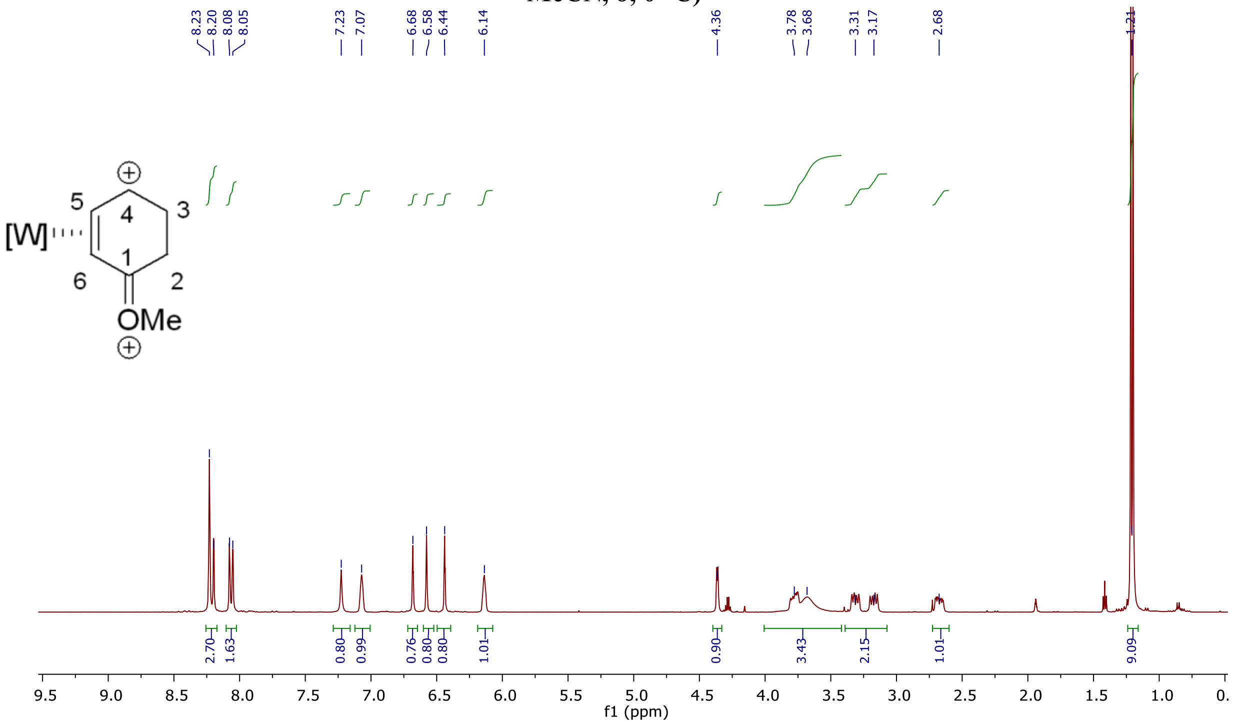

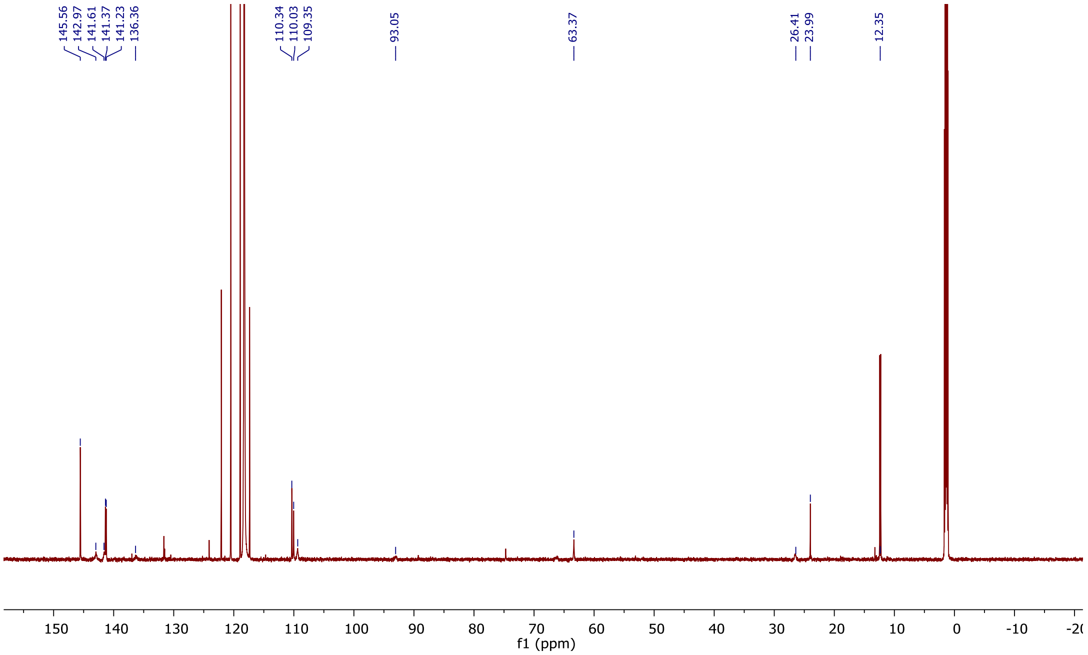

Supplementary Fig 4. Compound 15D ^{1}H NMR (800 MHz, d_6-acetone, δ, 25 °C) and ^{13}C NMR (800 MHz, d_6-acetone, δ, 25 °C)

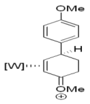

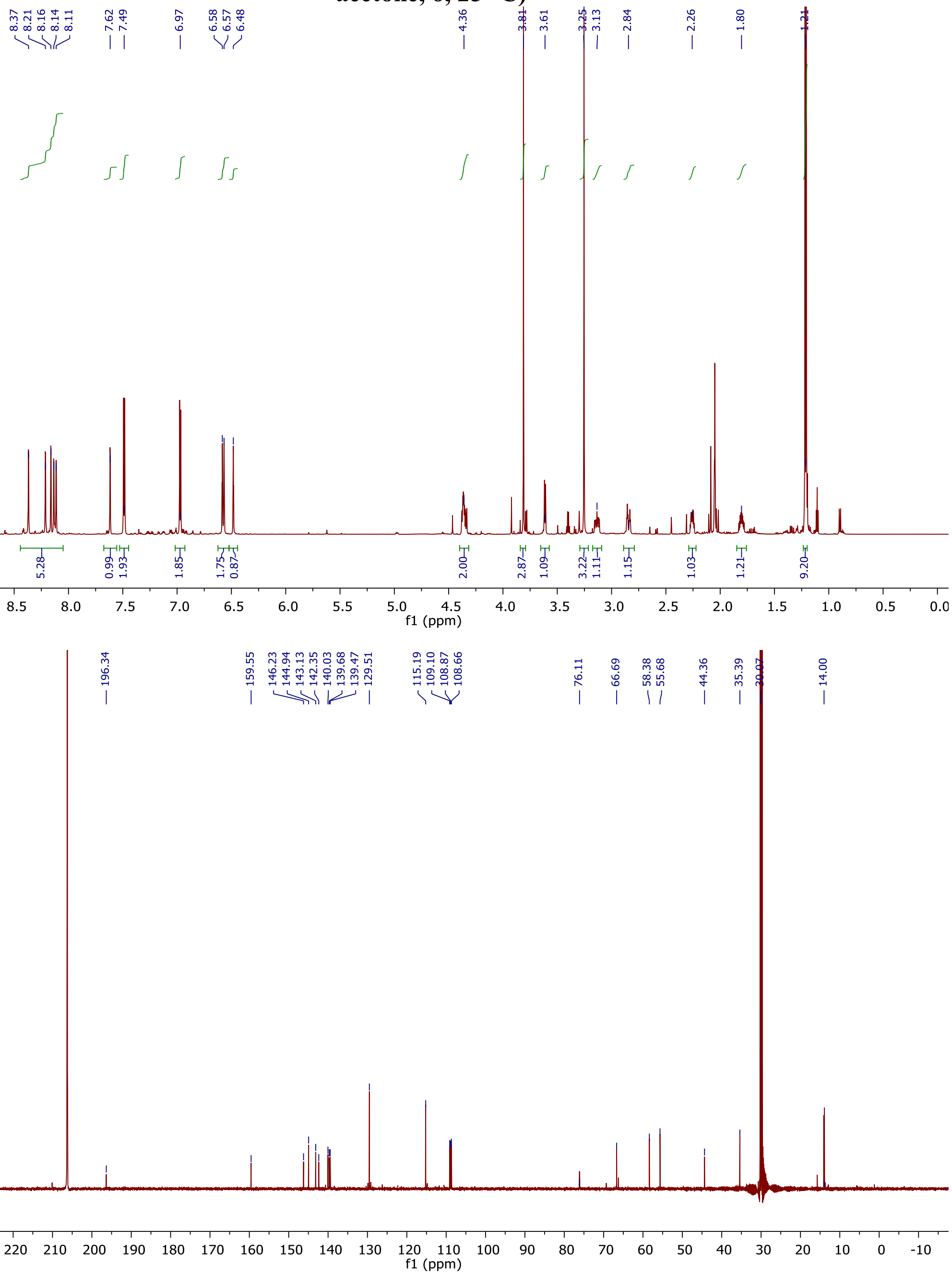

Supplementary Fig 5. Compound 16D ^{1}H NMR (800 MHz, d_6-acetone, δ, 25 °C) and ^{13}C NMR (800 MHz, d_6-acetone, δ, 25 °C)

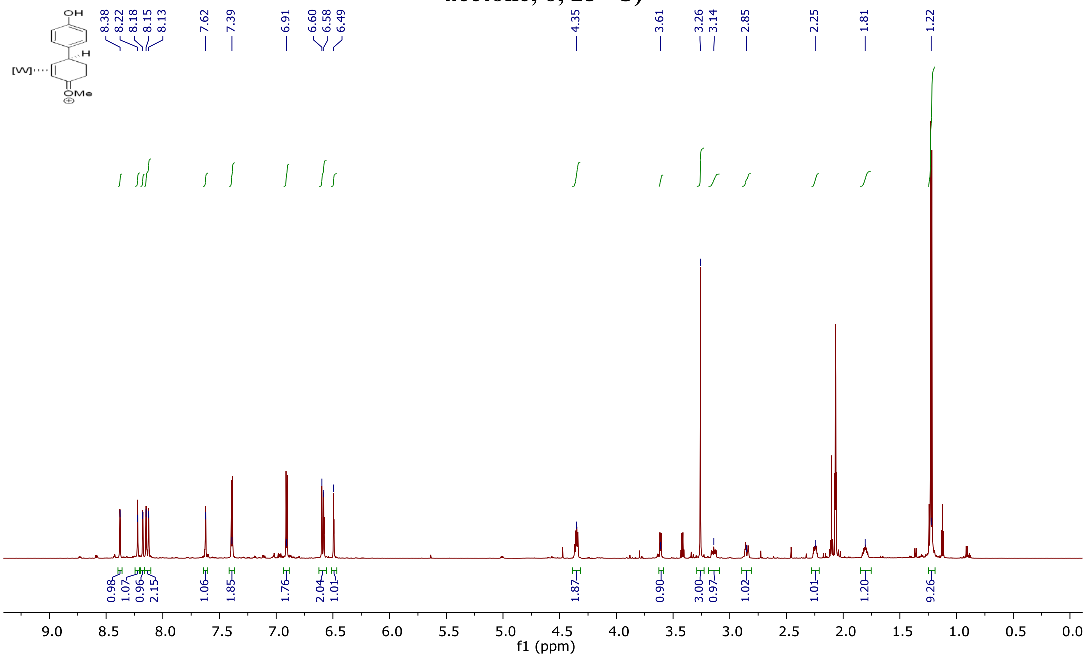

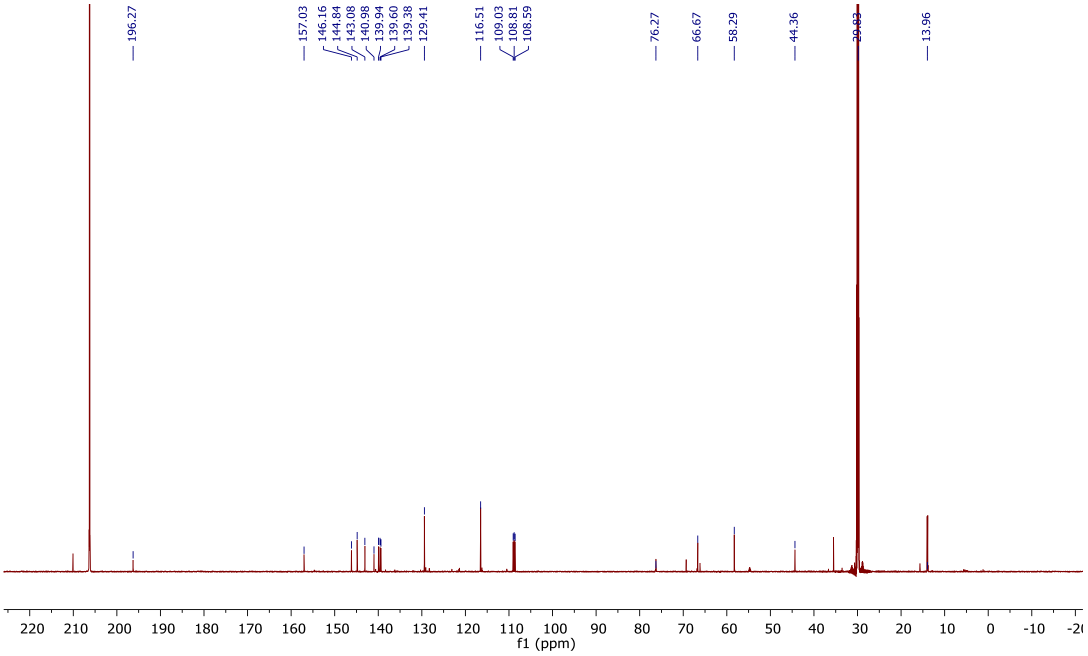

Supplementary Fig 6. Compound 17D^{1}H NMR (800 MHz, *d$_6$*-acetone, δ, 25 °C) and ^{13}C NMR (800 MHz, *d$_6$*-acetone, δ, 25 °C)

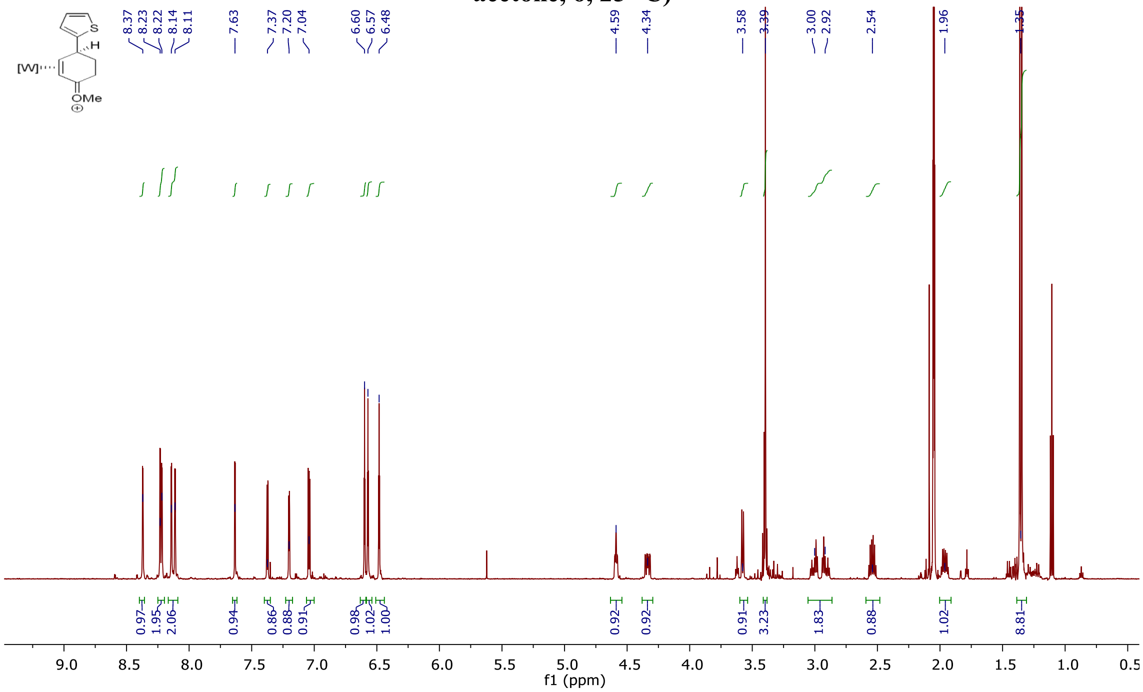

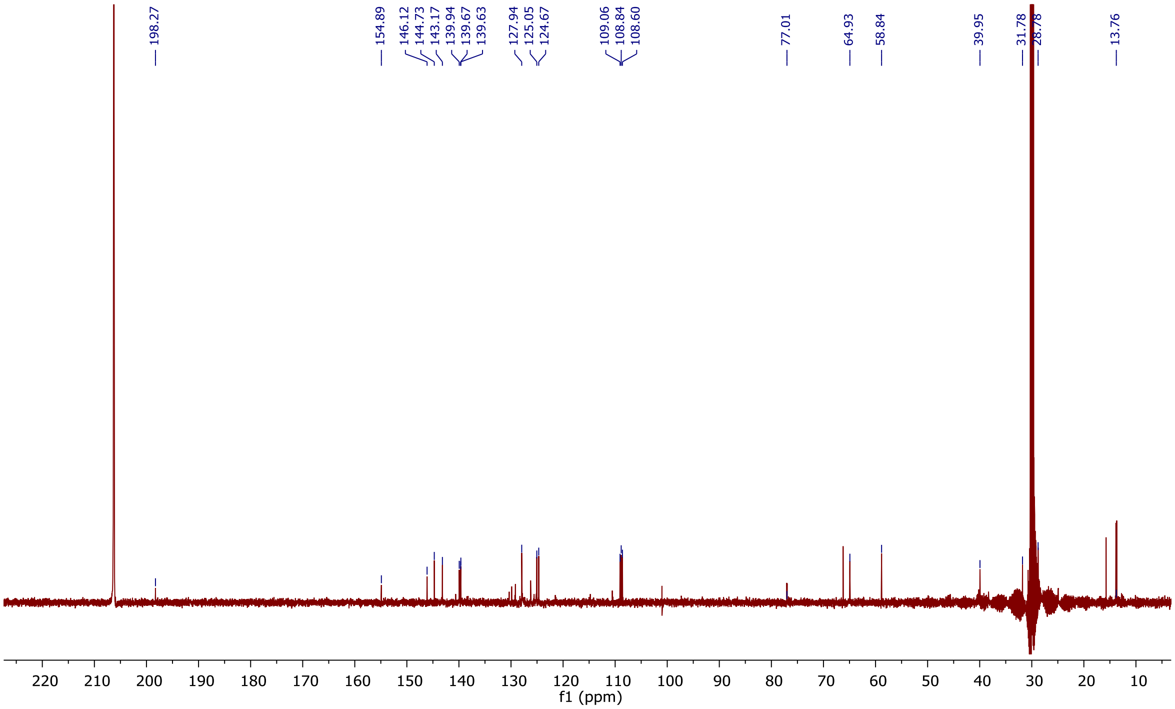

Supplementary Fig 7. Compound 17P ^{1}H NMR (600 MHz, d_6-acetone, δ, 25 °C) and ^{13}C NMR (800 MHz, d_6-acetone, δ, 25 °C)

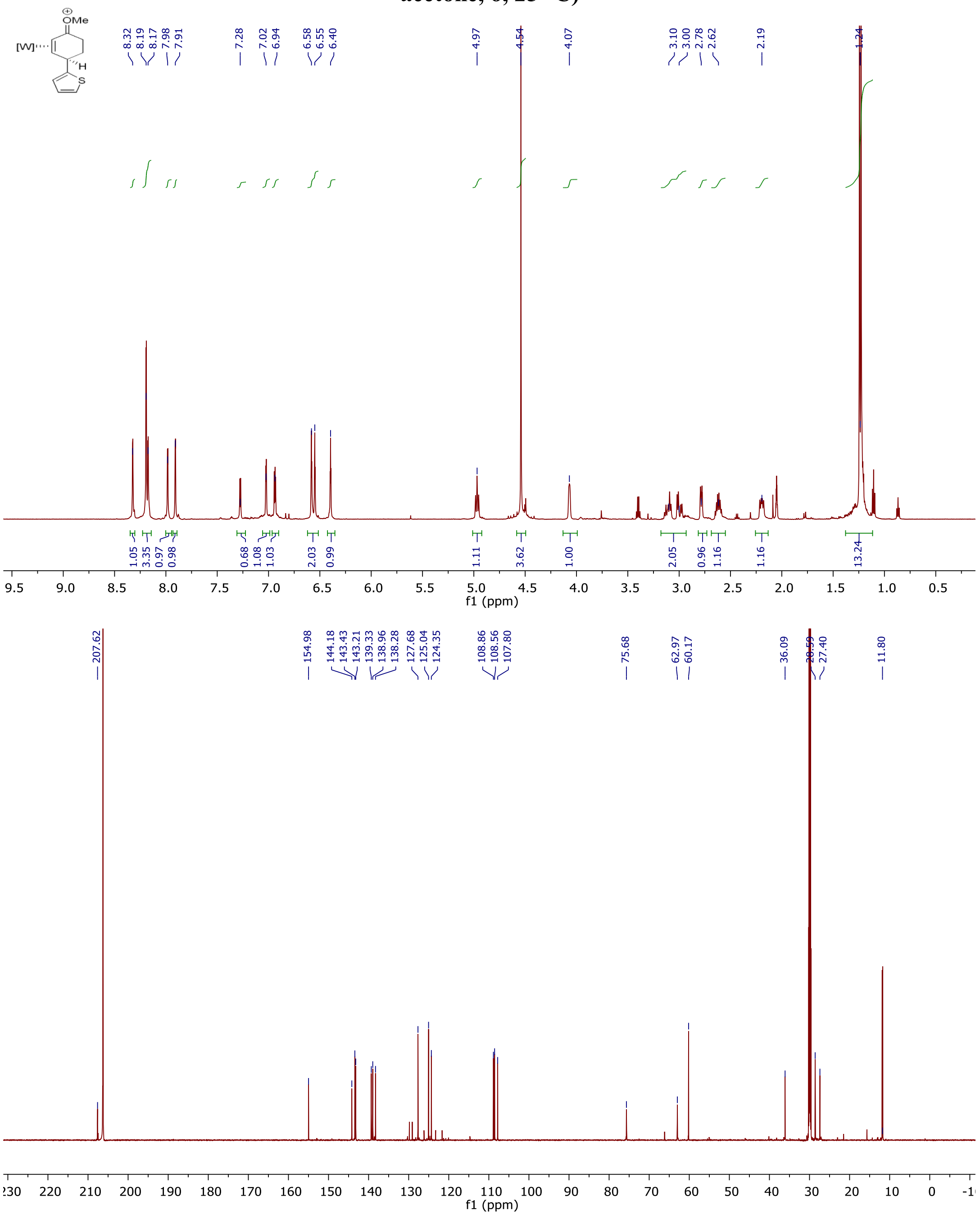

Supplementary Fig 8. Compound 6D ^{1}H NMR (800 MHz, d_3-MeCN, δ, 25 °C) and ^{13}C NMR (800 MHz, d_3-MeCN, δ, 25 °C)

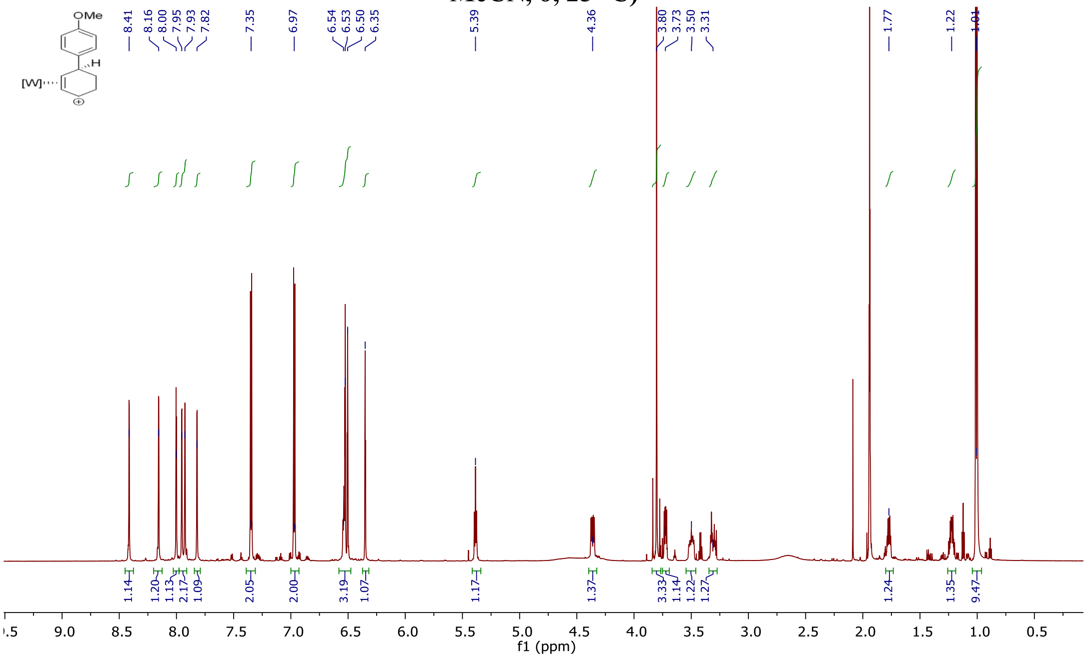

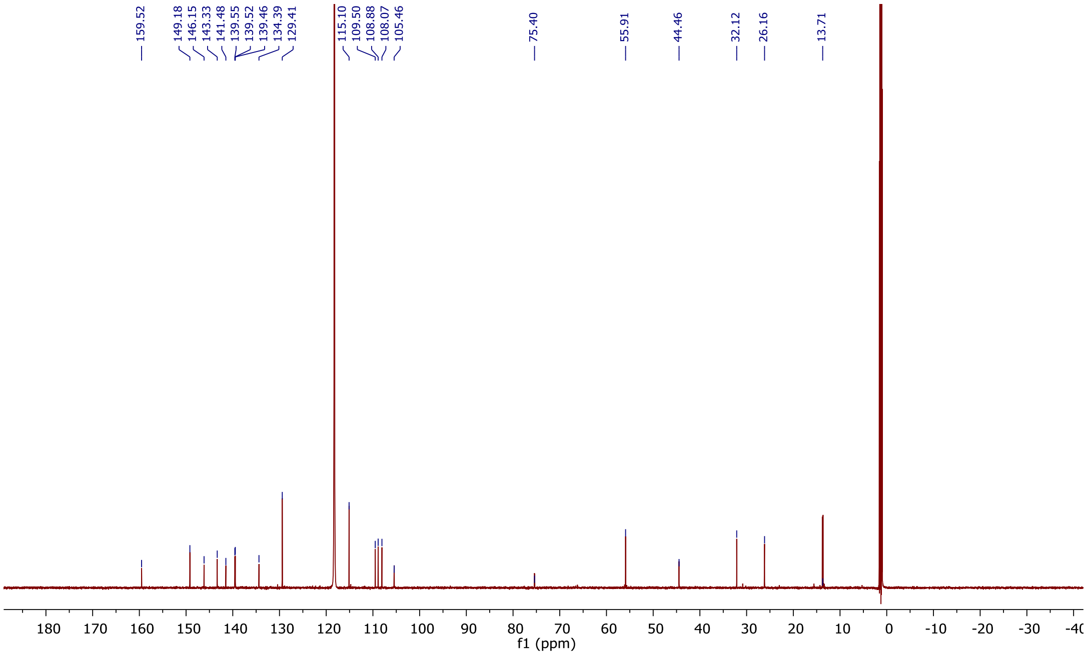

Supplementary Fig 9. Compound 7D ^{1}H NMR (800 MHz, d_3-MeCN, δ, 25 °C) and ^{13}C NMR (800 MHz, d_3-MeCN, δ, 25 °C)

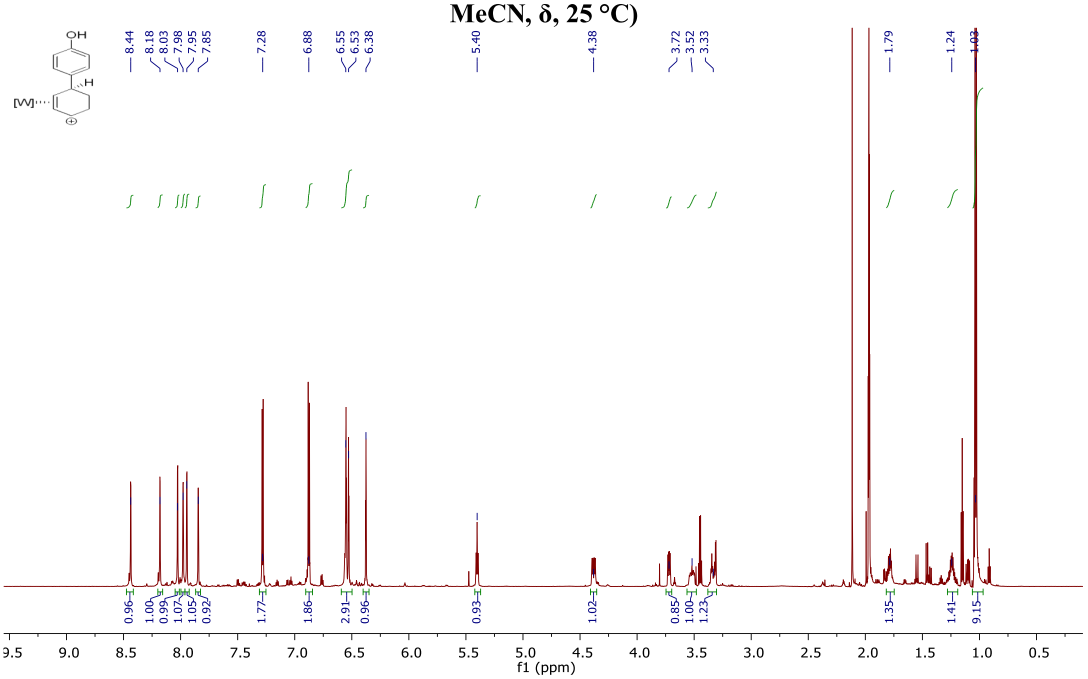

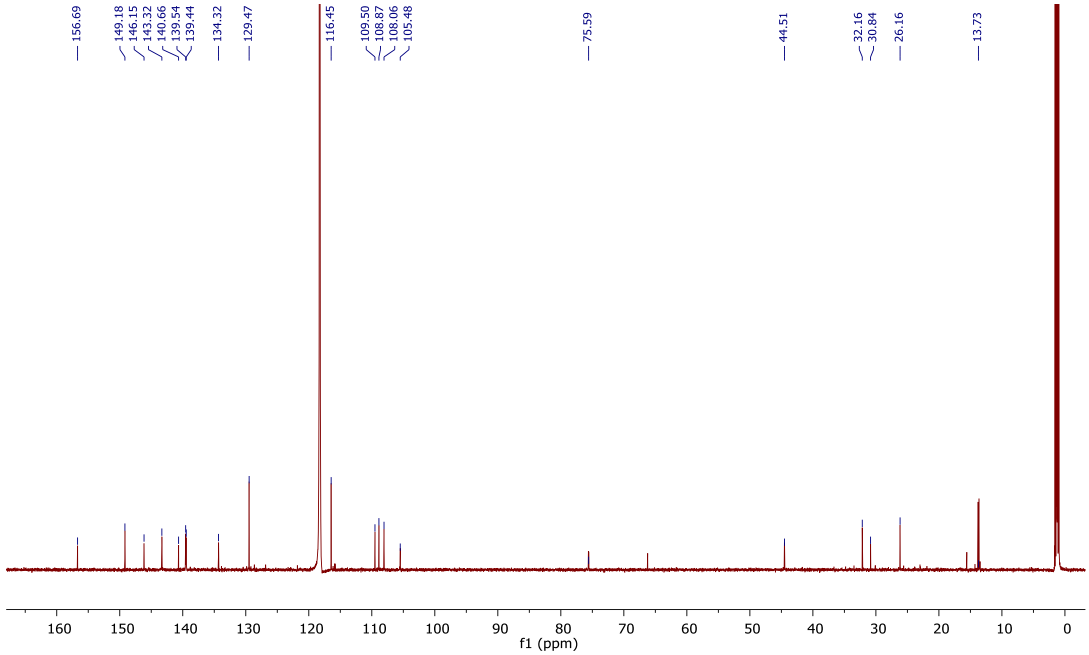

Supplementary Fig 10. Compound 8D ^{1}H NMR (600 MHz, d_3-MeCN, δ, 25 °C) and ^{13}C NMR (800 MHz, d_3-MeCN, δ, 25 °C)

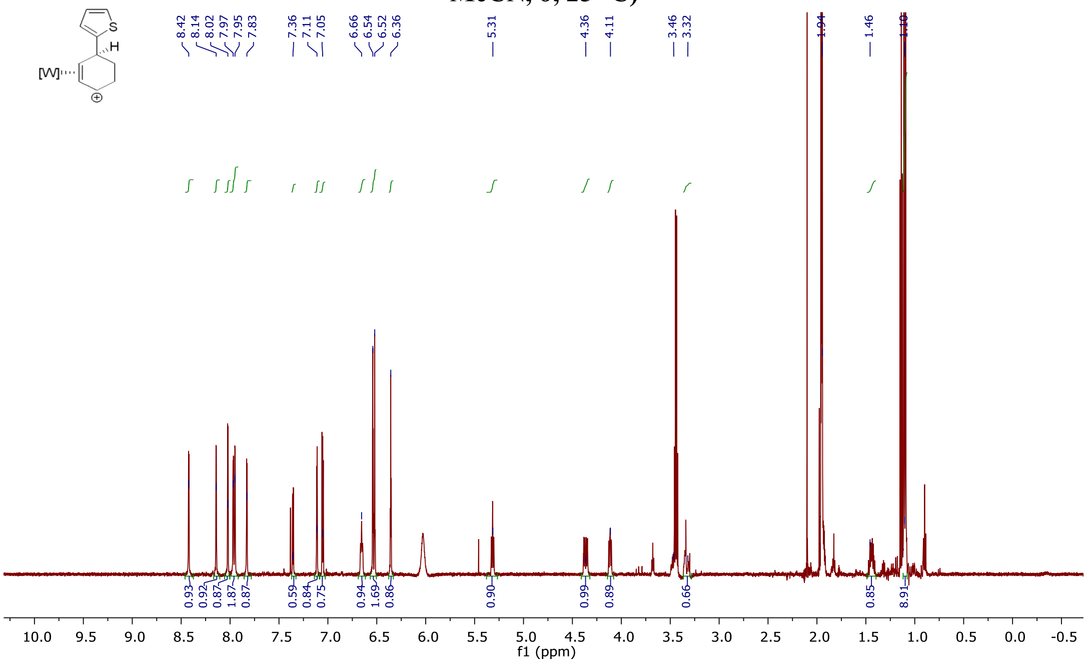

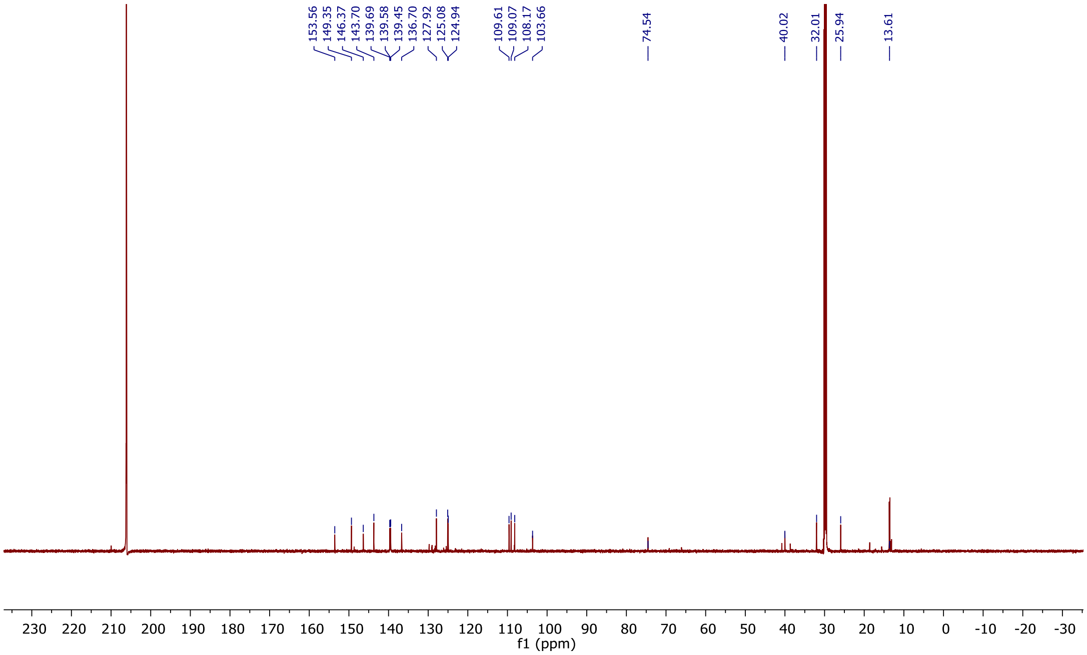

Supplementary Fig 11. Compound 8P ^{1}H NMR (600 MHz, d_3-MeCN, δ, 25 °C) and ^{13}C NMR (800 MHz, d_3-MeCN, δ, 25 °C)

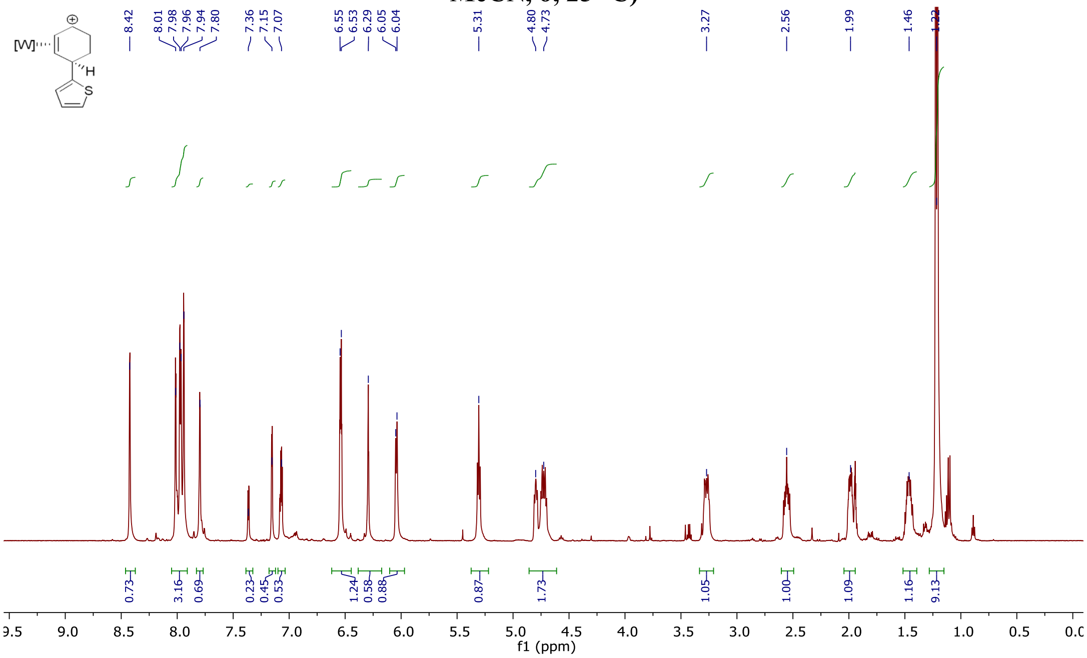

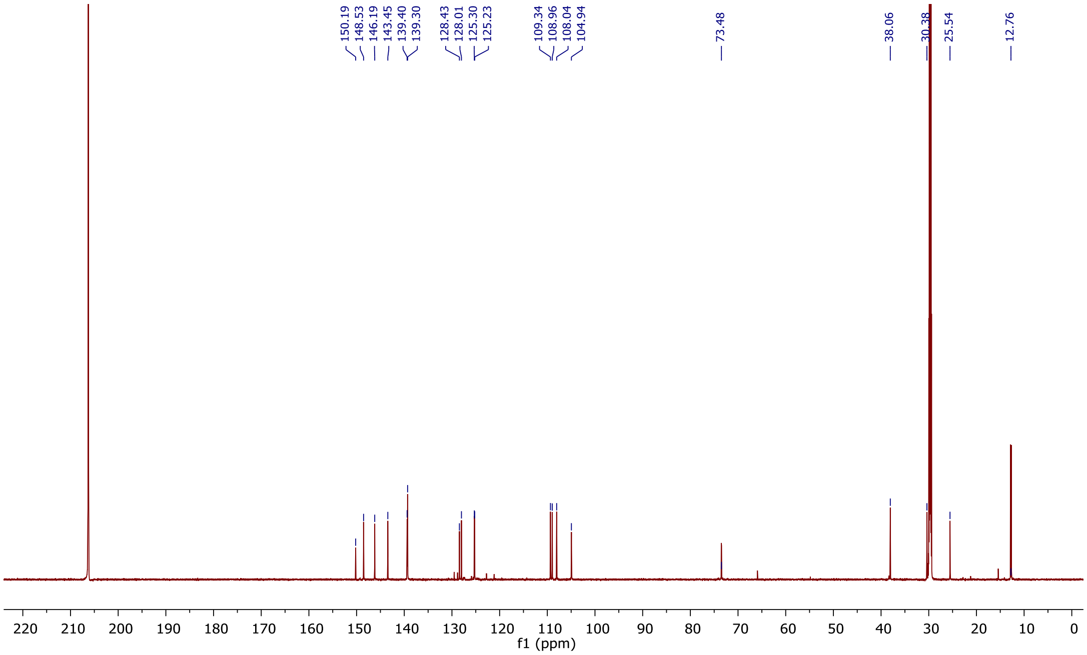

Supplementary Fig 12. Compound 9D ^{1}H NMR (600 MHz, d_3-MeCN, δ, 25 °C) and ^{13}C NMR (800 MHz, d_3-MeCN, δ, 25 °C)

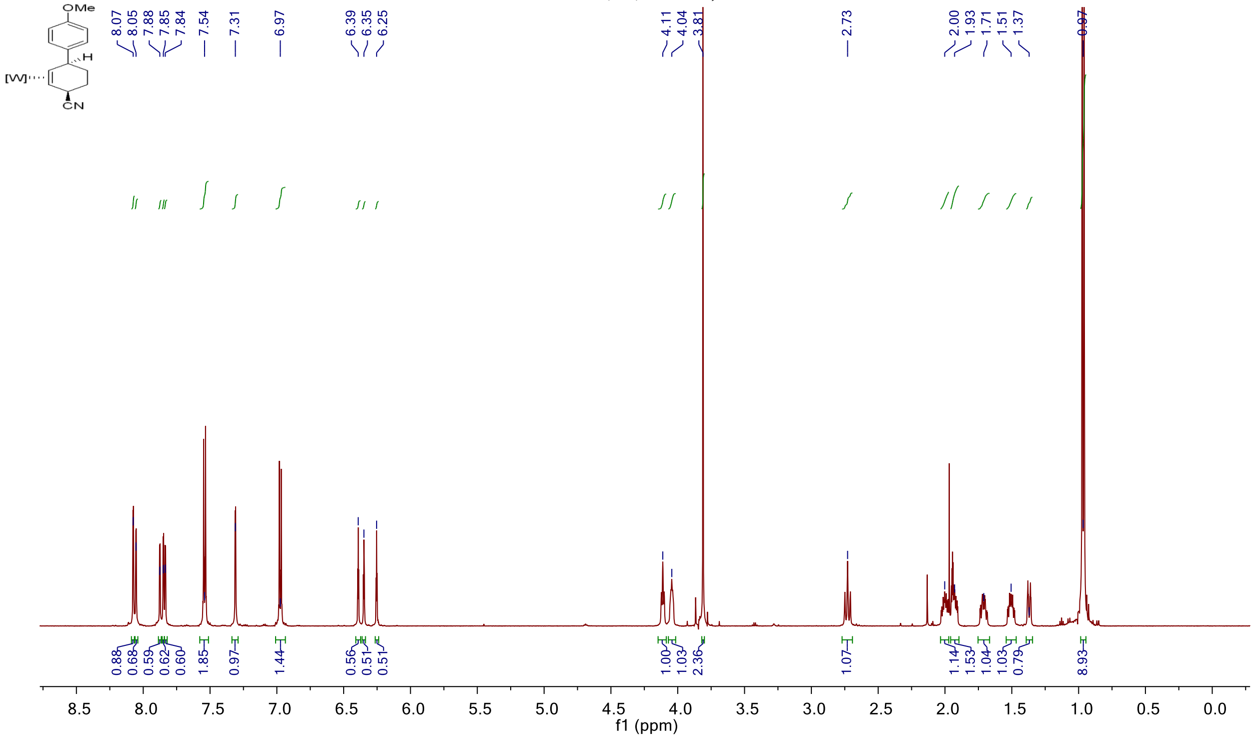

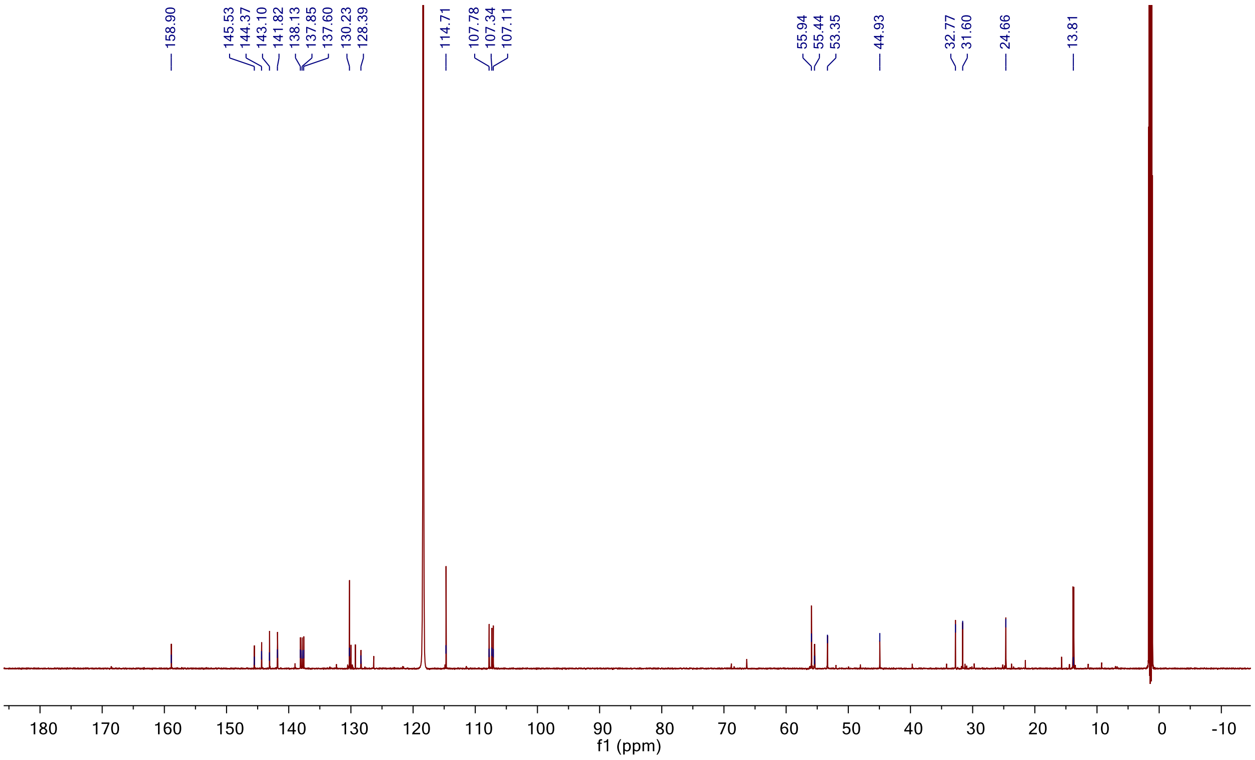

Supplementary Fig 13. Compound 10D ^{1}H NMR (600 MHz, d_6-acetone, δ, 25 °C) and ^{13}C NMR (800 MHz, d_6-acetone, δ, 25 °C)

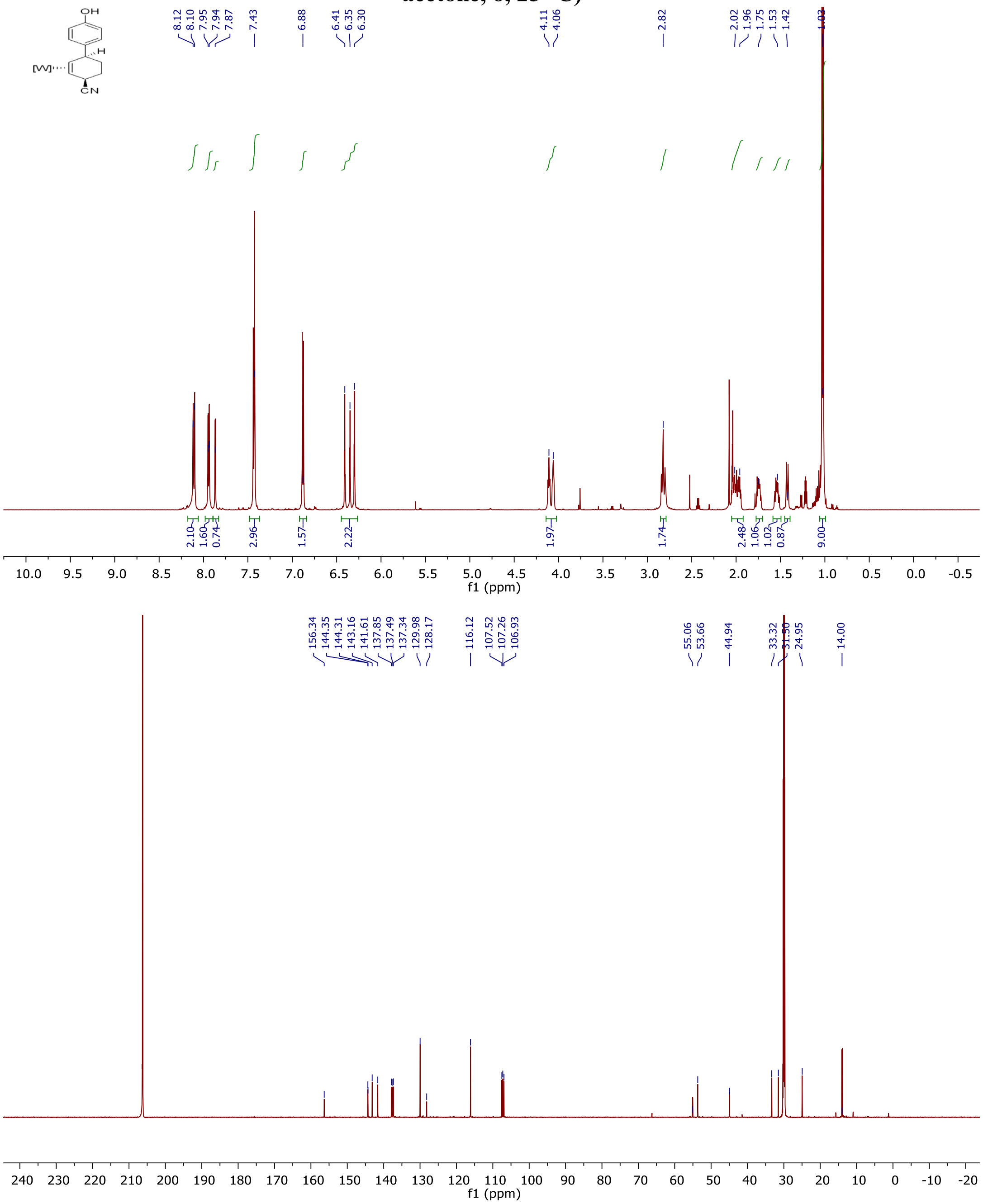

Supplementary Fig 14. Compound 11D ^{1}H NMR (800 MHz, d_6-acetone, δ, 25 °C) and ^{13}C NMR (800 MHz, d_6-acetone, δ, 25 °C)

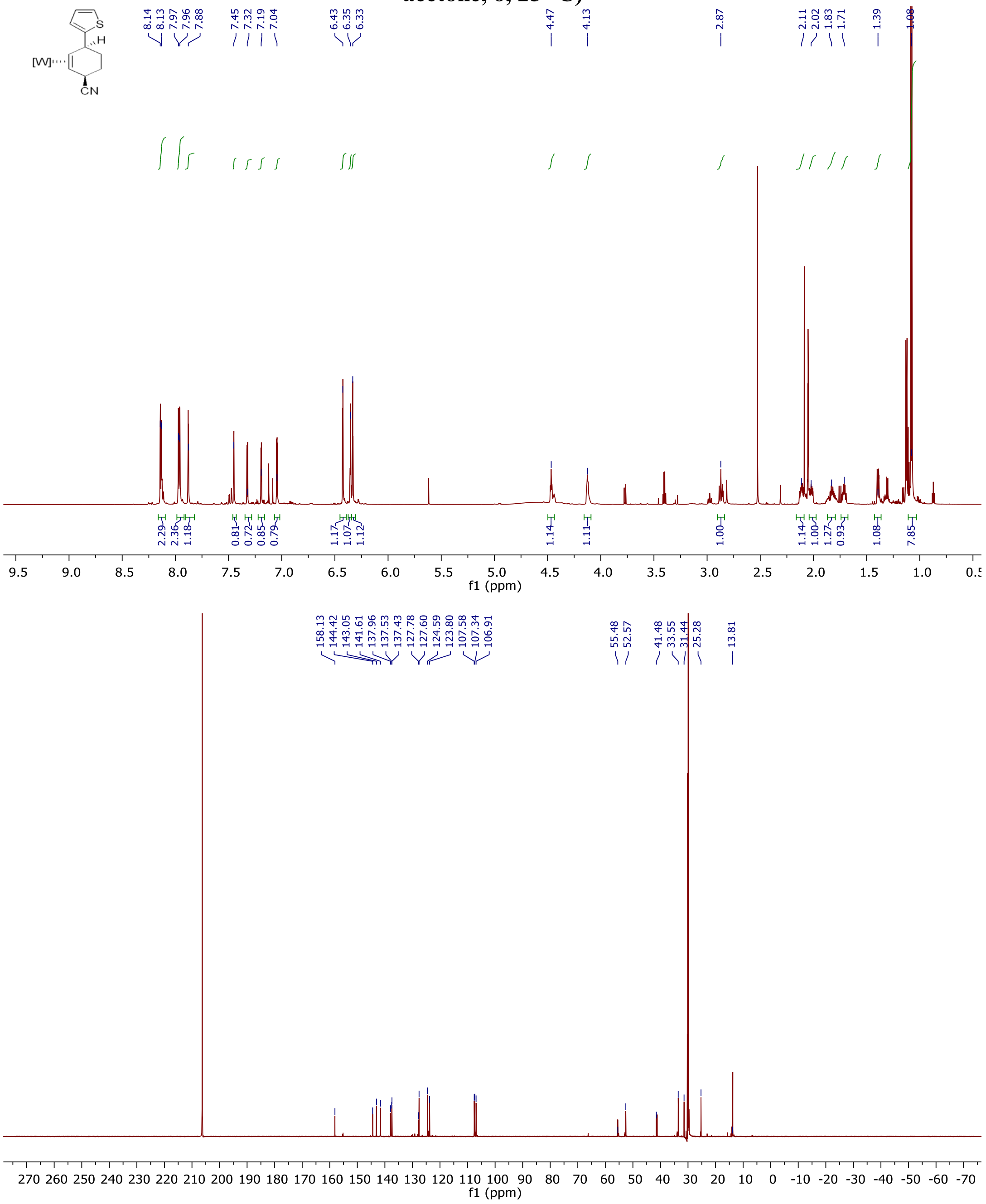

Supplementary Fig 15. Compound 11P(a) and 11P(b) ^{1}H NMR (800 MHz, CD_2Cl_2, δ, 25 °C) and NOESY (800 MHz, CD_2Cl_2, δ, 25 °C)

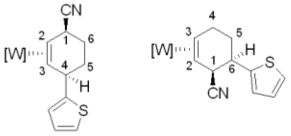

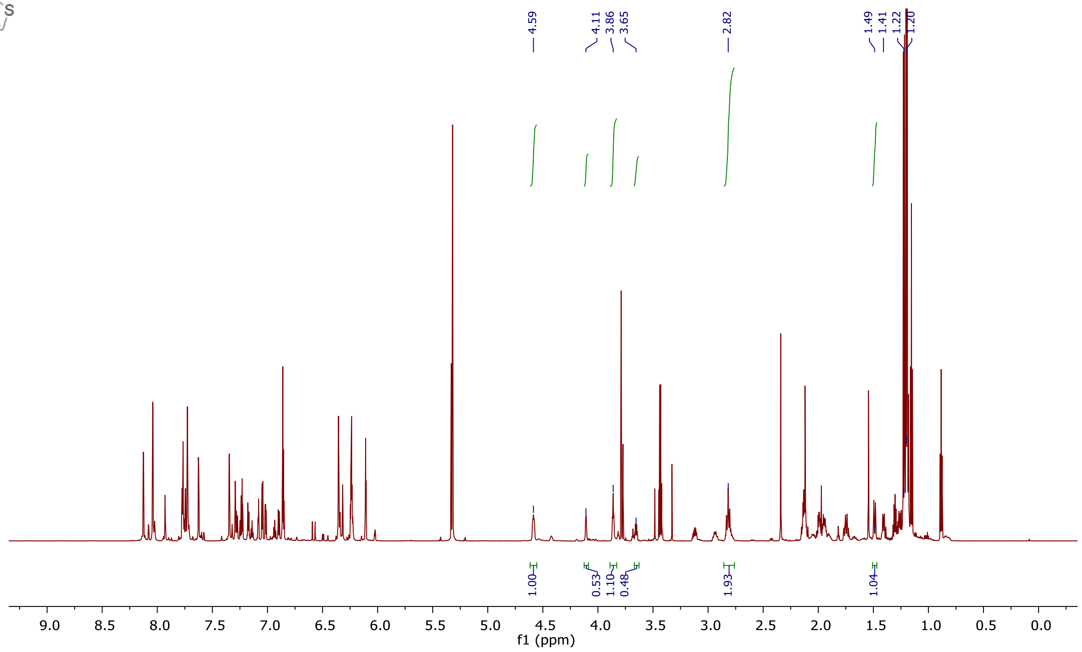

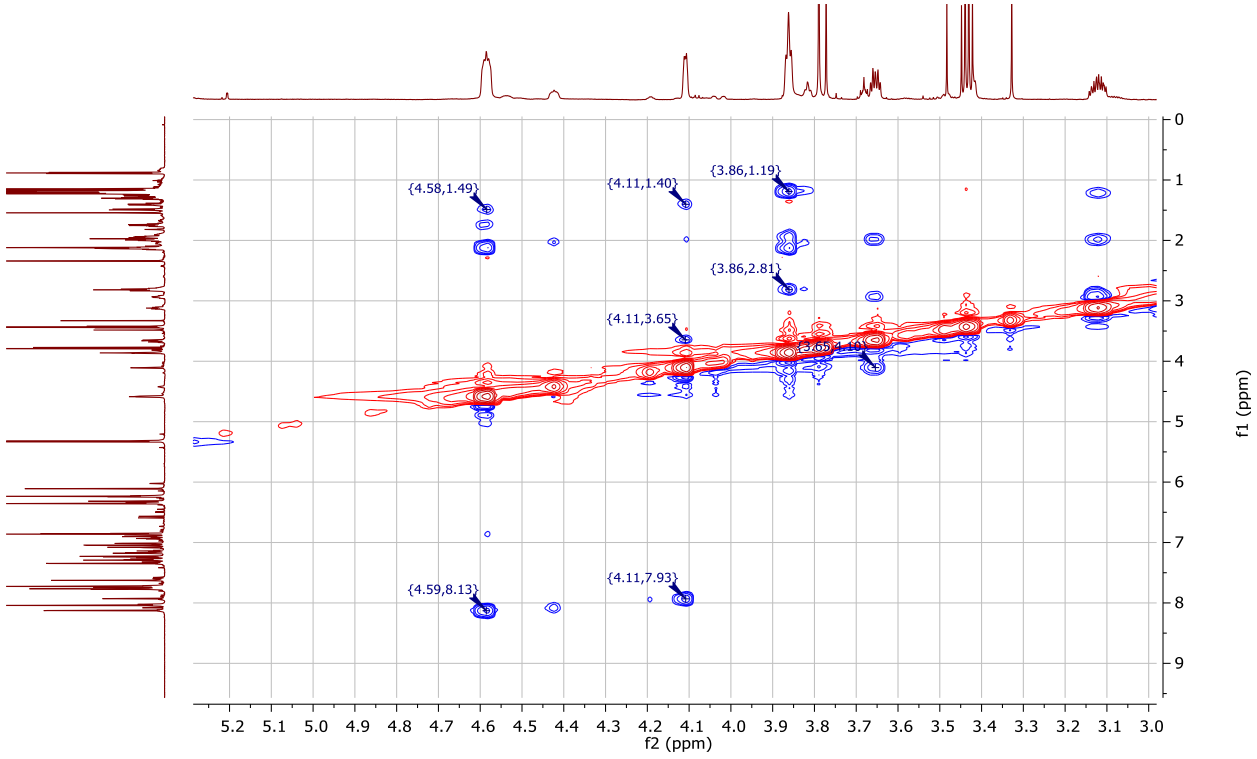

Supplementary Fig 16. Compound 21 ^{1}H NMR (800 MHz, d_6-acetone, δ, 25 °C) and ^{13}C NMR (800 MHz, d_6-acetone, δ, 25 °C)

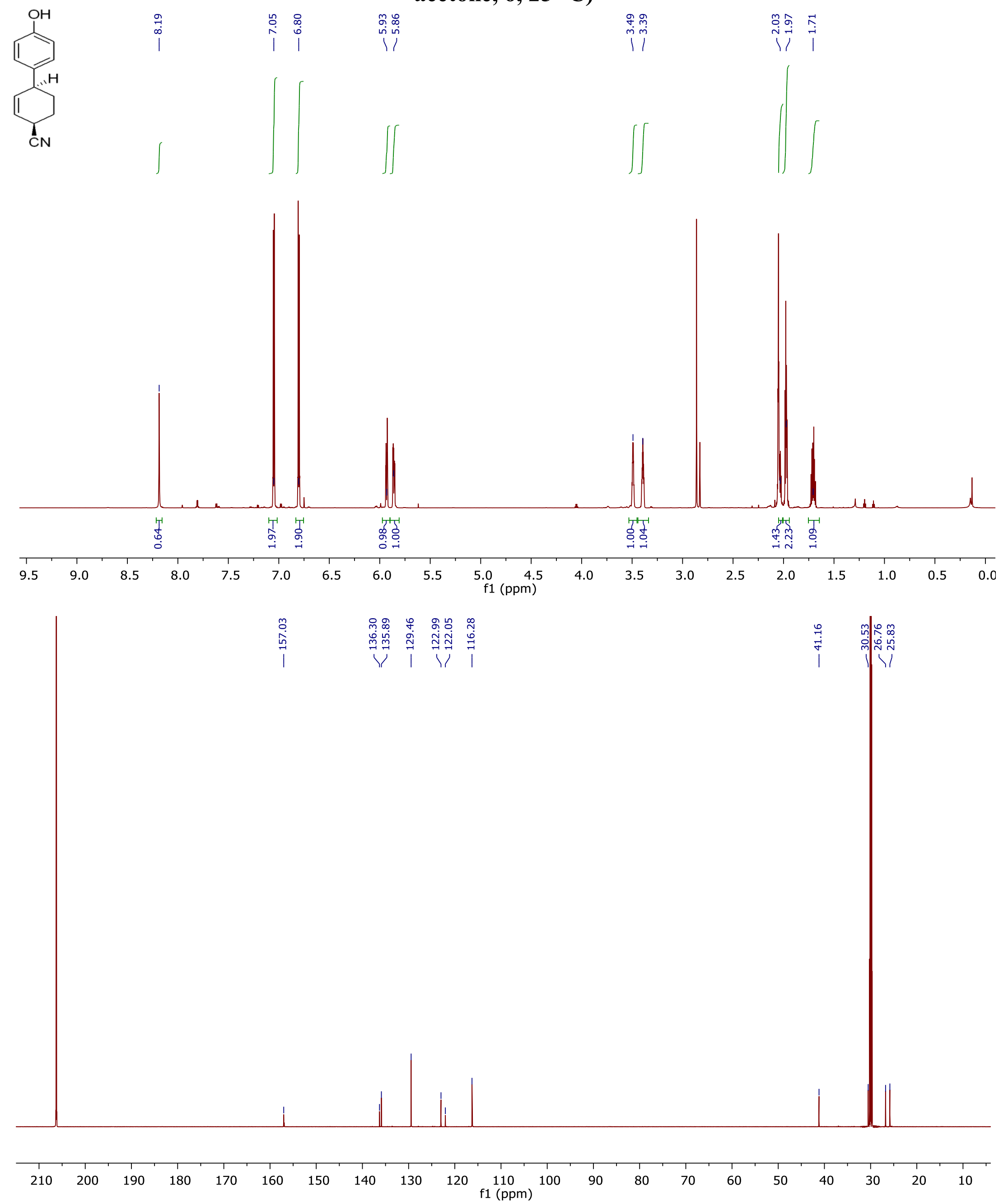

Supplementary Fig 17. Compound 22 ^{1}H NMR (600 MHz, d_3-MeCN, δ, 25 °C) and ^{13}C NMR (800 MHz, d_3-MeCN, δ, 25 °C)

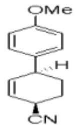

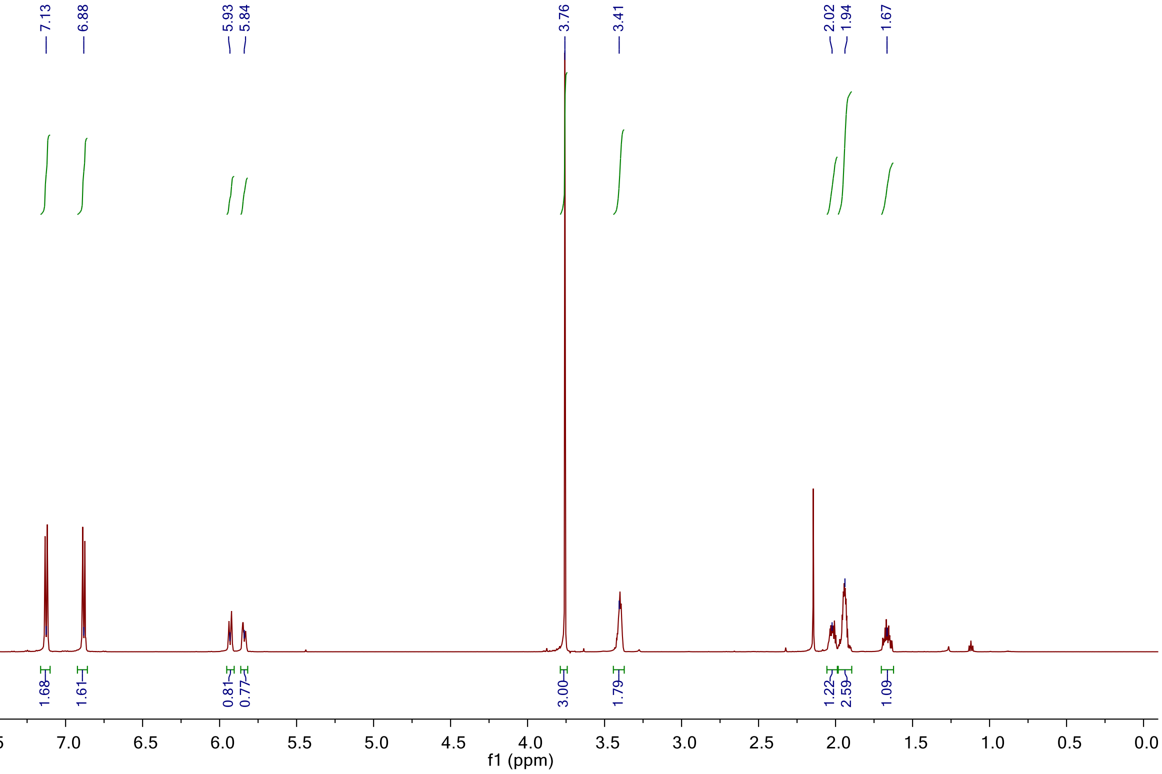

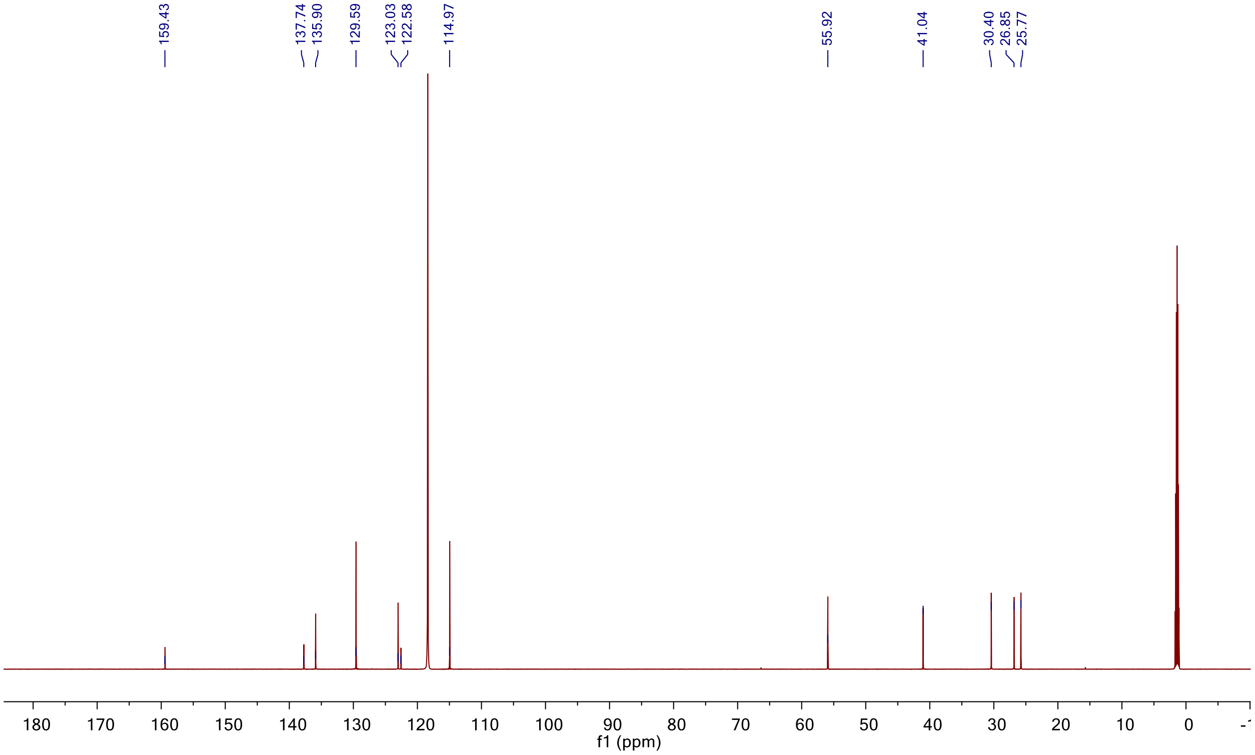

Supplementary Fig 18. Compound 23 ^{1}H NMR (600 MHz, d_6-acetone, δ, 25 °C) and ^{13}C NMR (800 MHz, d_6-acetone, δ, 25 °C)

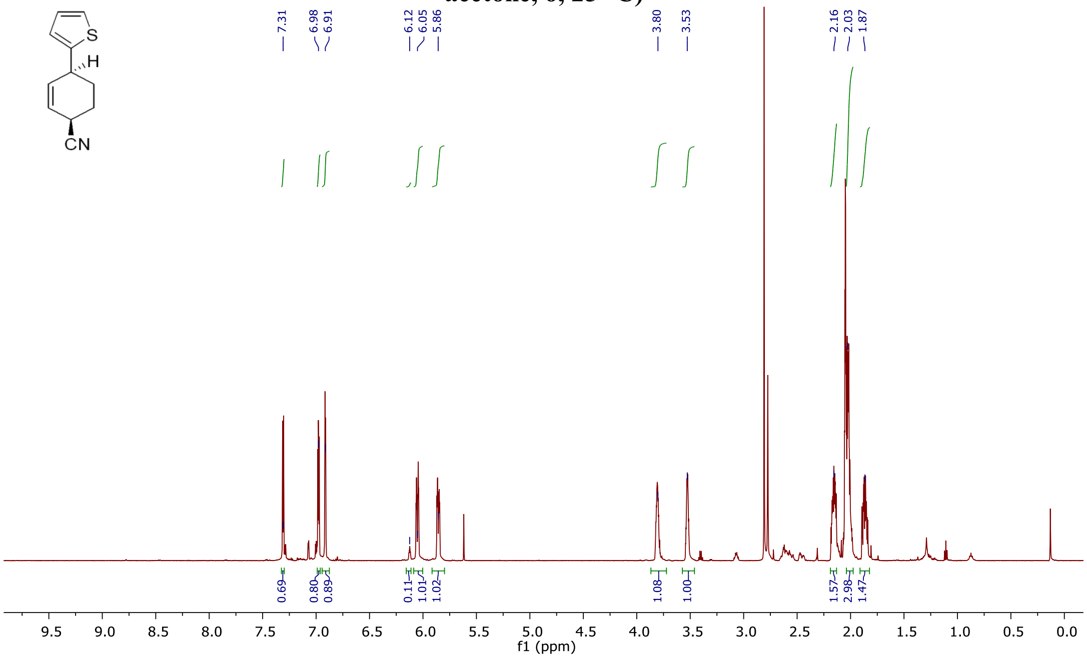

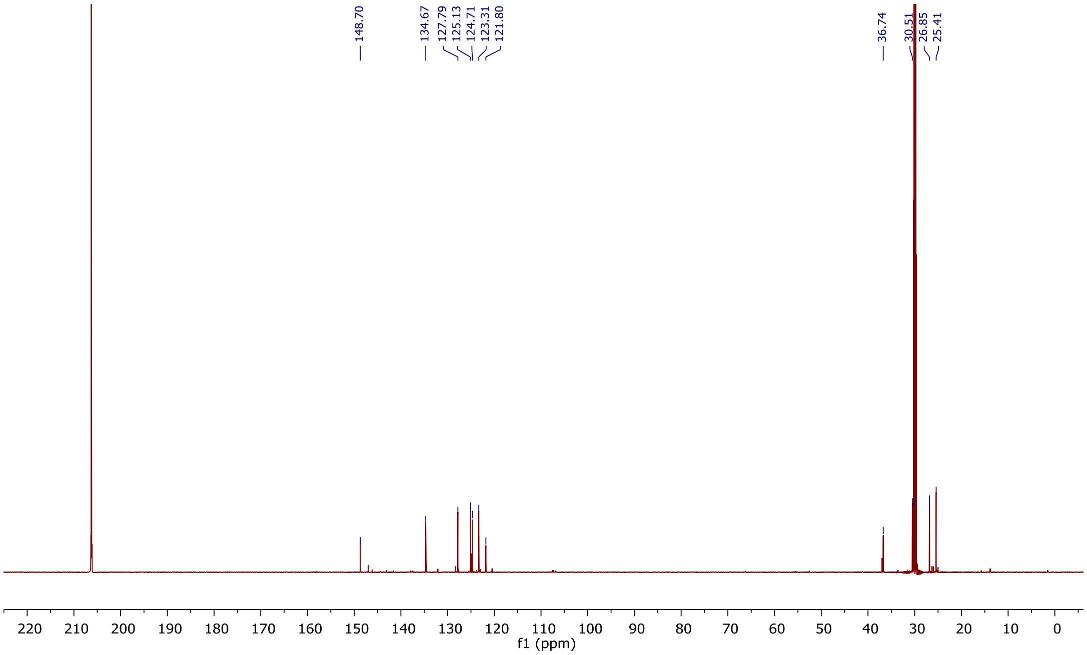

Supplementary Fig 19. Compound 6D-*d₆* ¹H NMR (800 MHz, *d₃*-MeCN, δ, 25 °C)

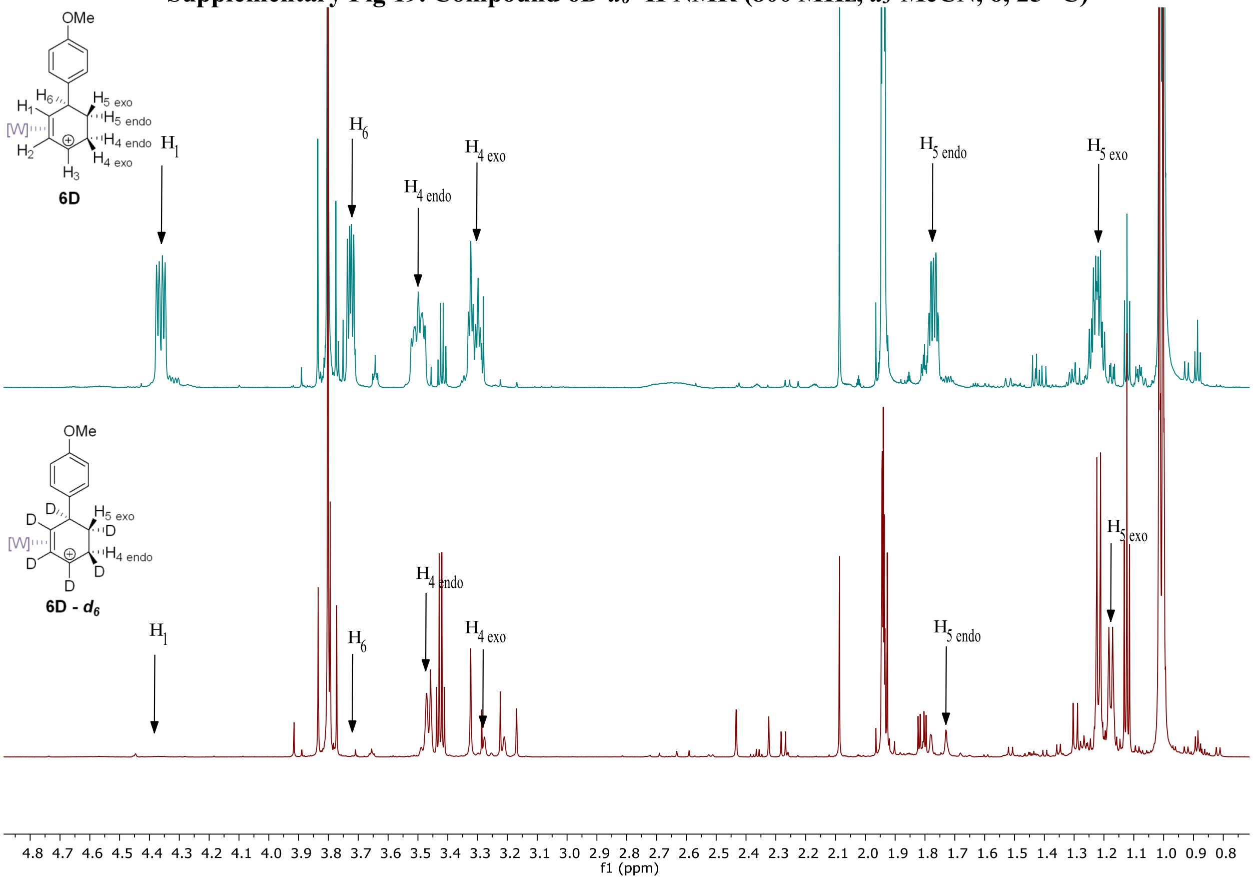

Supplementary Fig 20. Compound 6D-Mo ¹H NMR (800 MHz, *d₆*-acetone, δ, 25 °C)

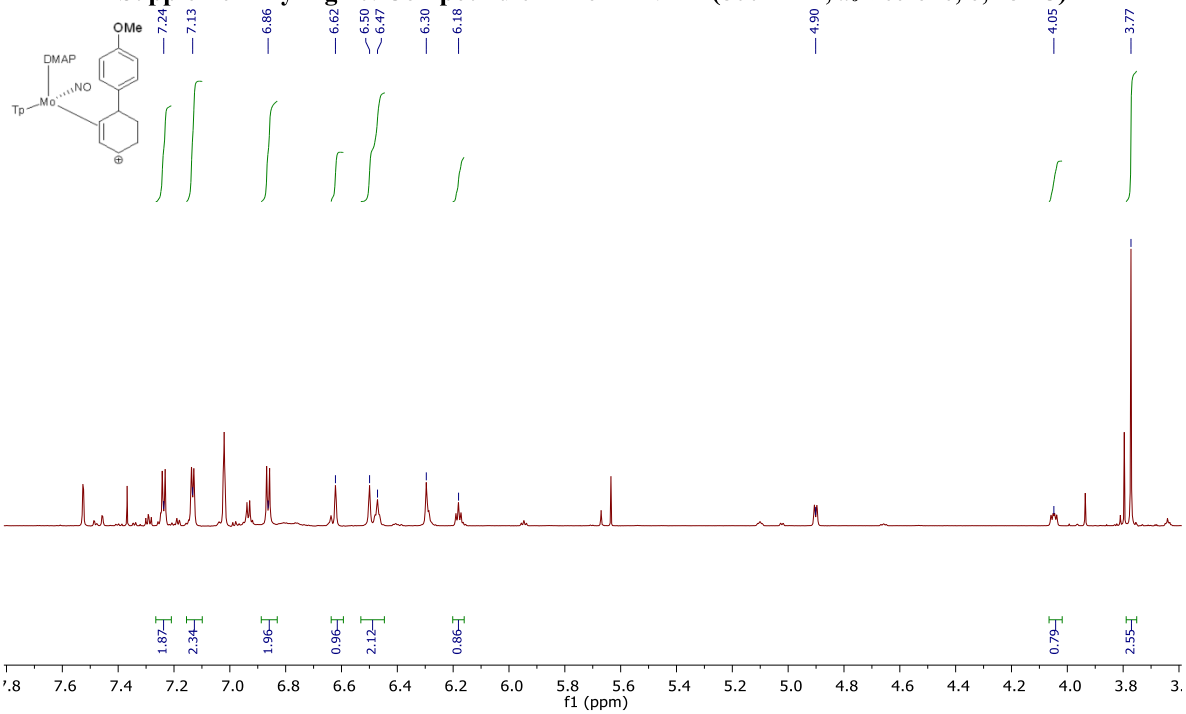

Supplementary Fig 21. Compound 24D ^{1}H NMR (800 MHz, CD_2Cl_2, δ, 25 °C) and ^{13}C NMR (800 MHz, CD_2Cl_2, δ, 25 °C)

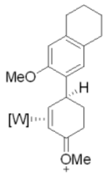

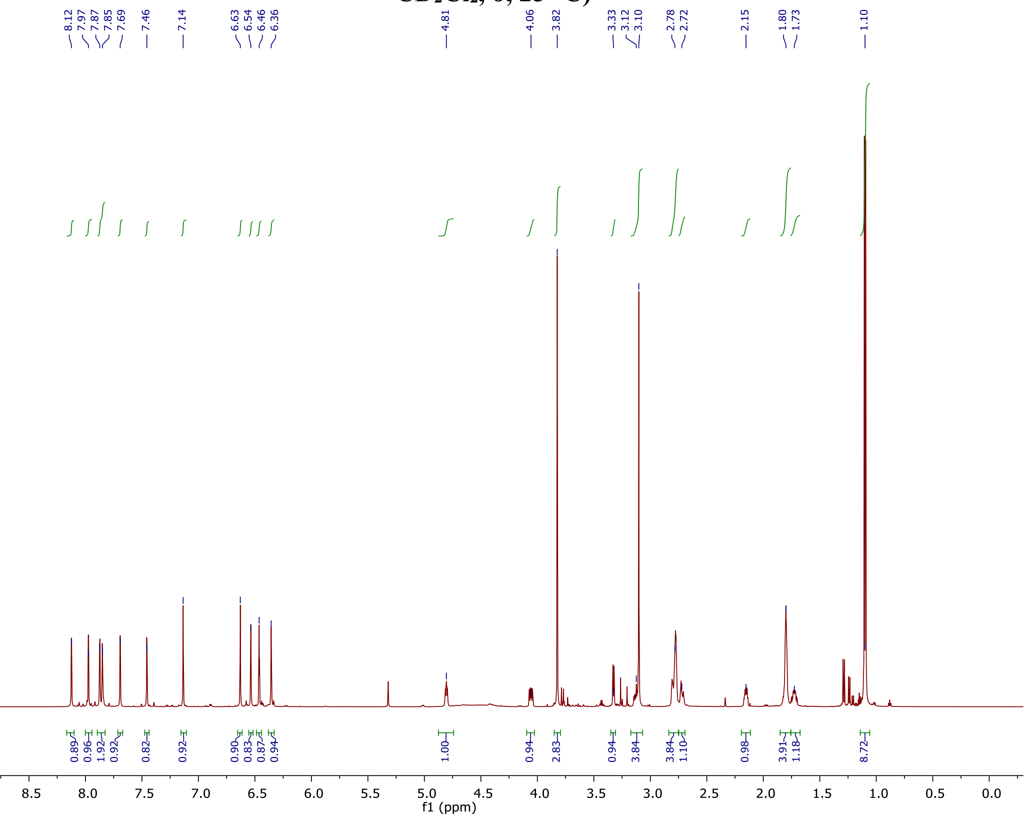

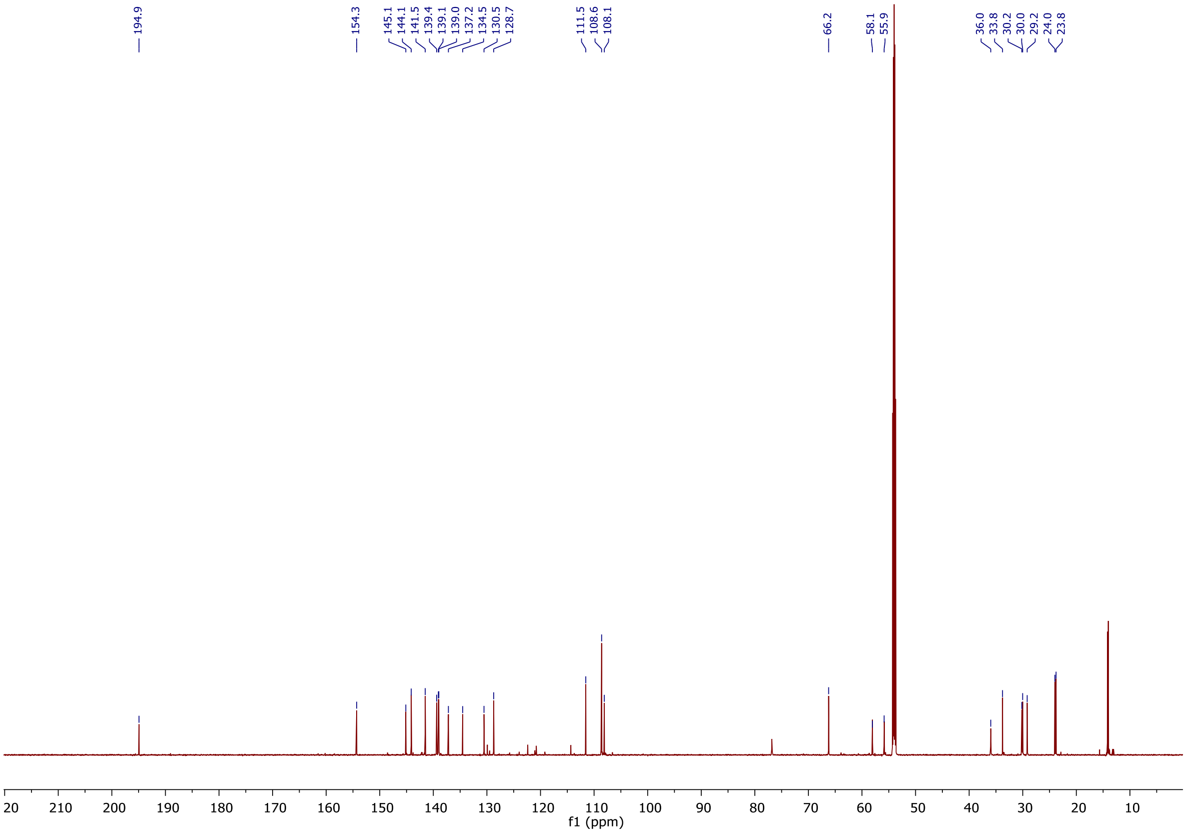

Supplementary Fig 22. Compound 25D ^{1}H NMR (800 MHz, d_3-MeCN, δ, 25 °C) and ^{31}P NMR (500 MHz, d_3-MeCN, δ, 25 °C)

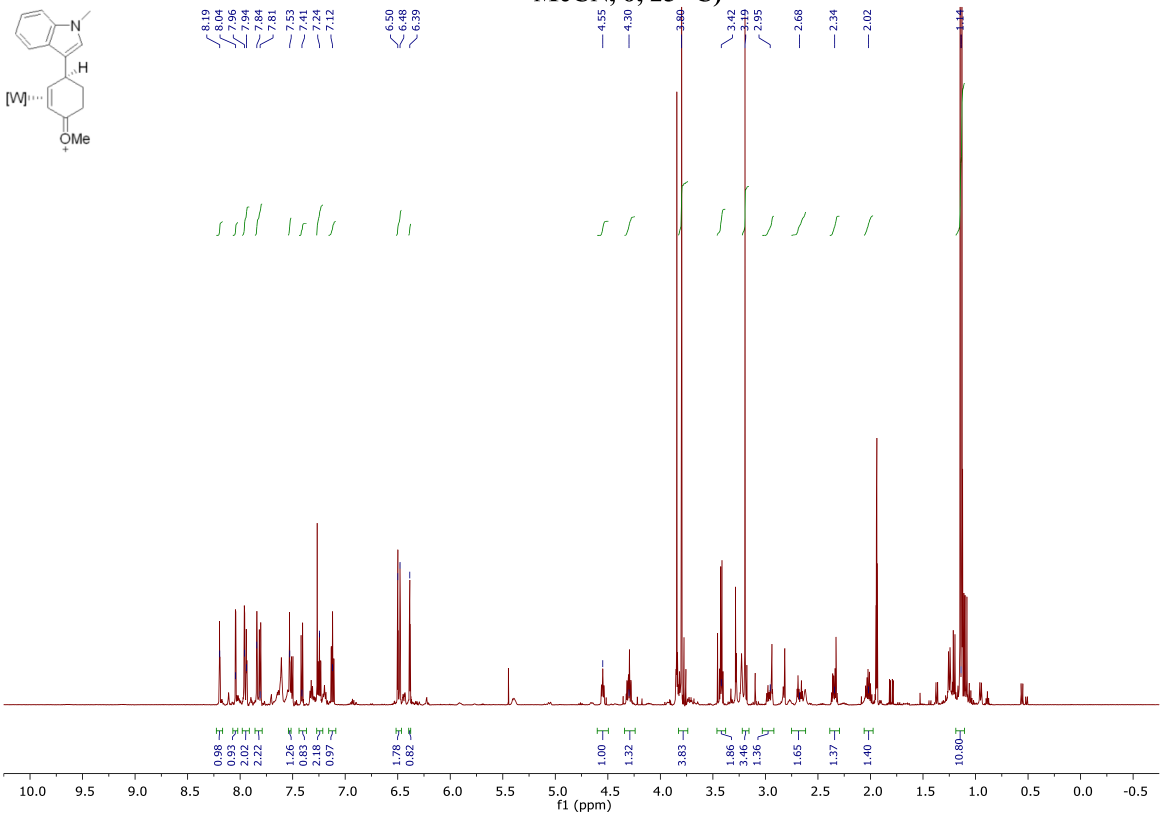

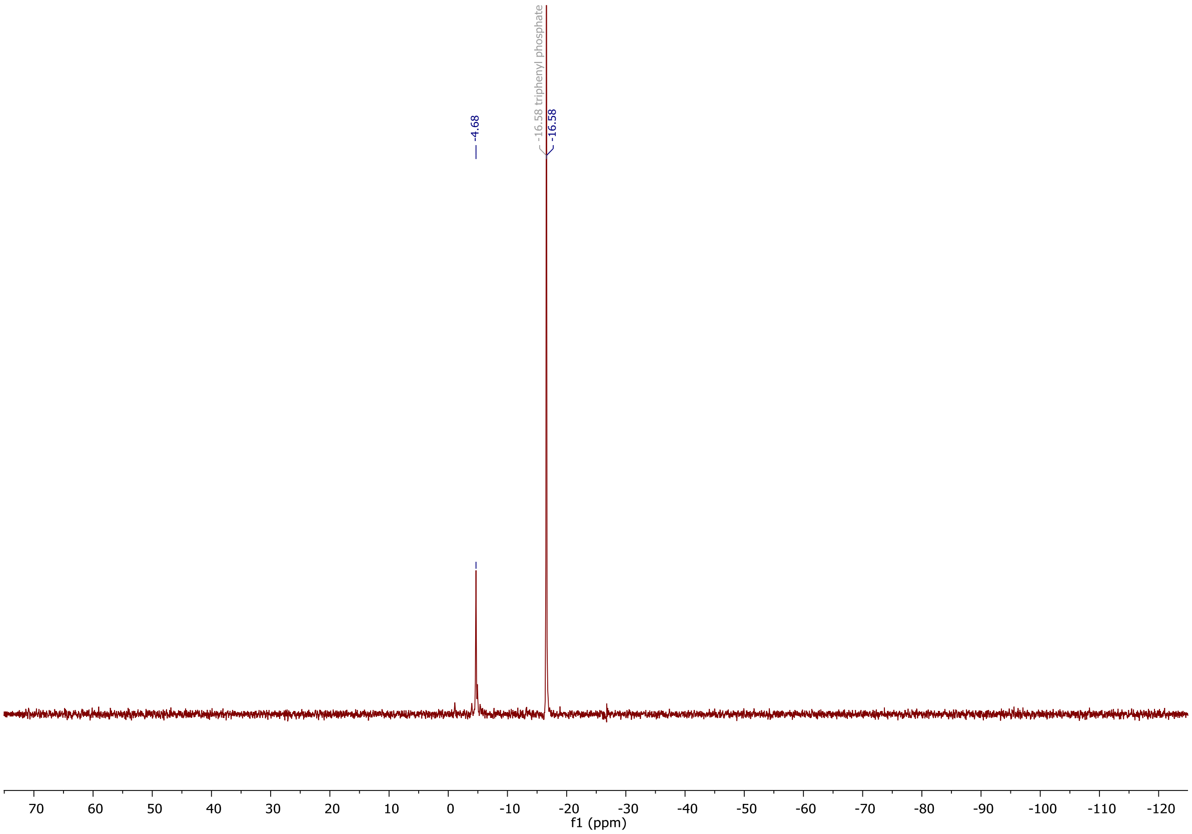

Supplementary Fig 23. Compound 26D ^{1}H NMR (800 MHz, CD_2Cl_2, δ, 25 °C) and ^{13}C NMR (800 MHz, CD_2Cl_2, δ, 25 °C)

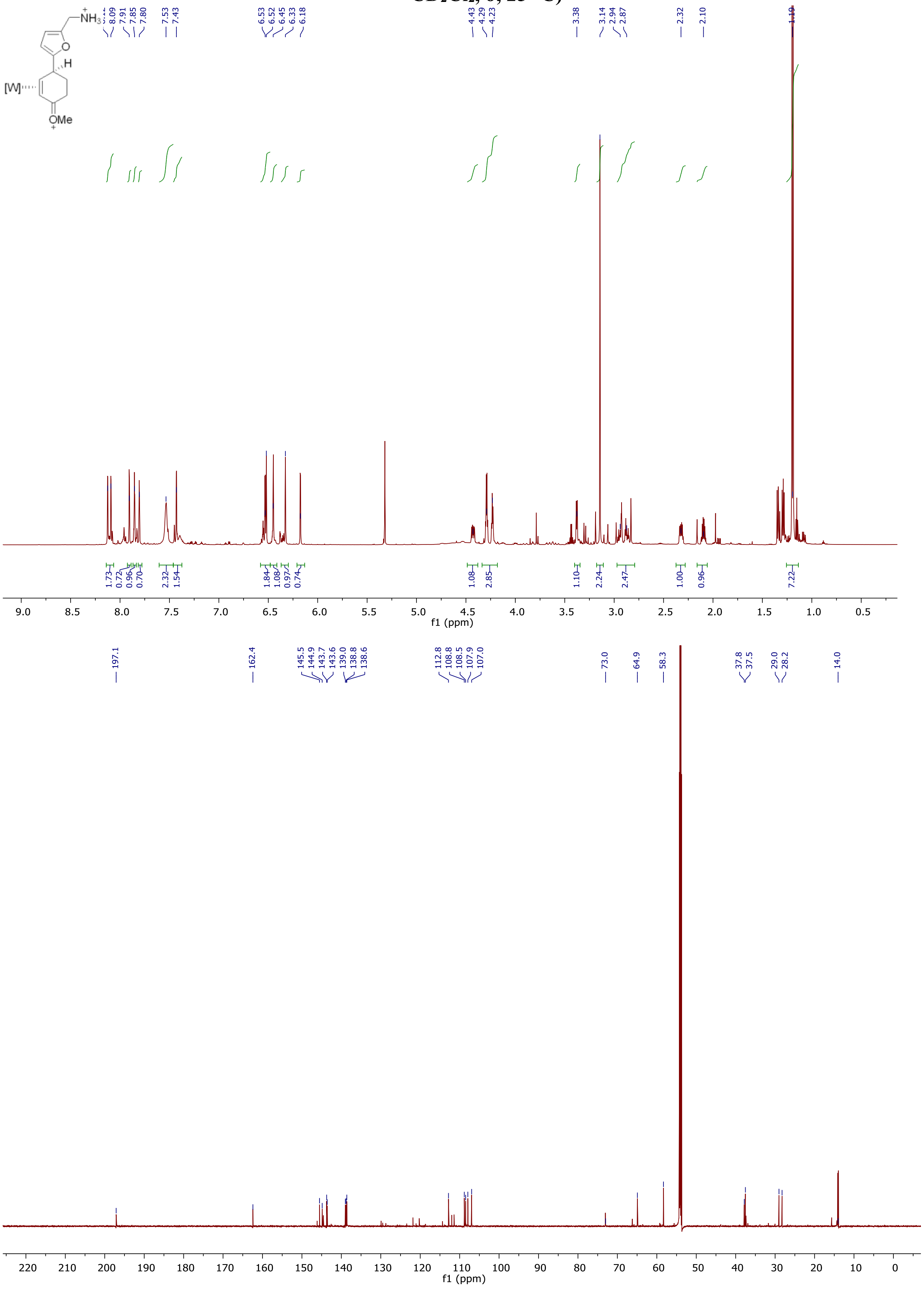

Supplementary Fig 24. Compound 27D ¹H NMR (800 MHz, d_3-MeCN, δ, 25 °C) and ¹³C NMR (800 MHz, d_3-MeCN, δ, 25 °C)

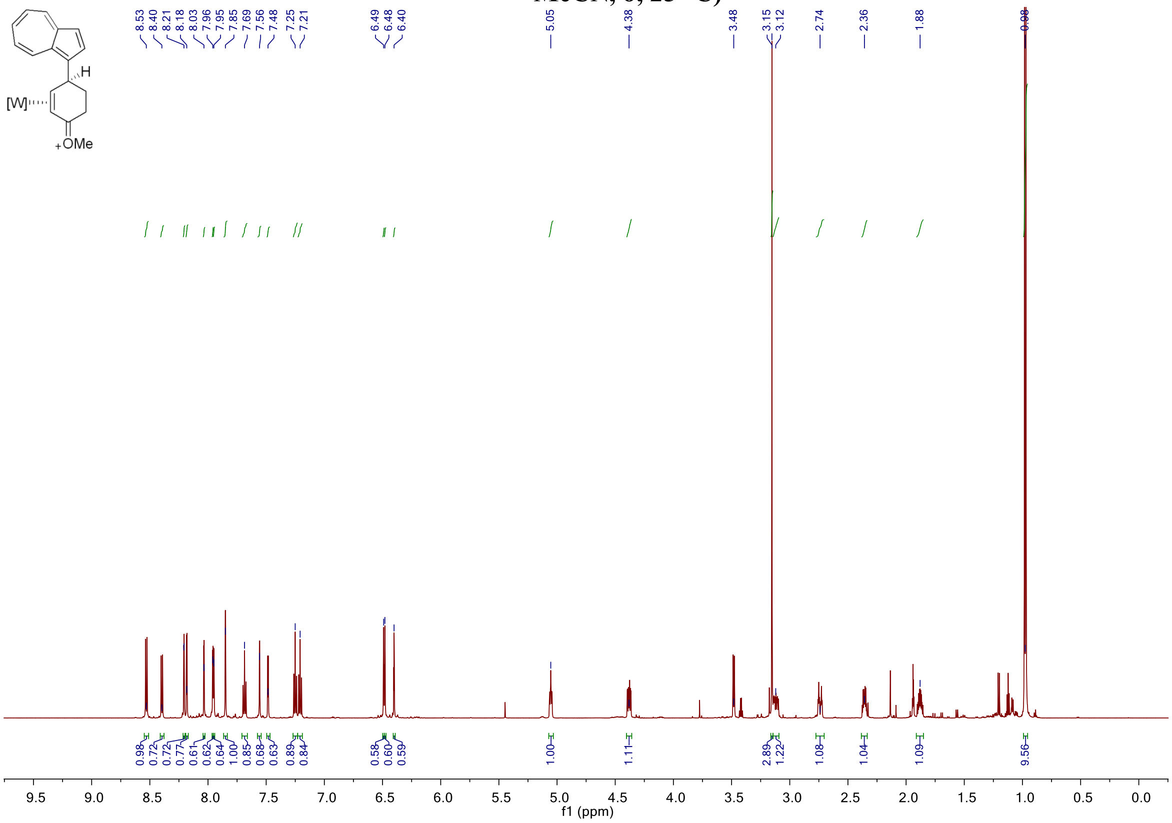

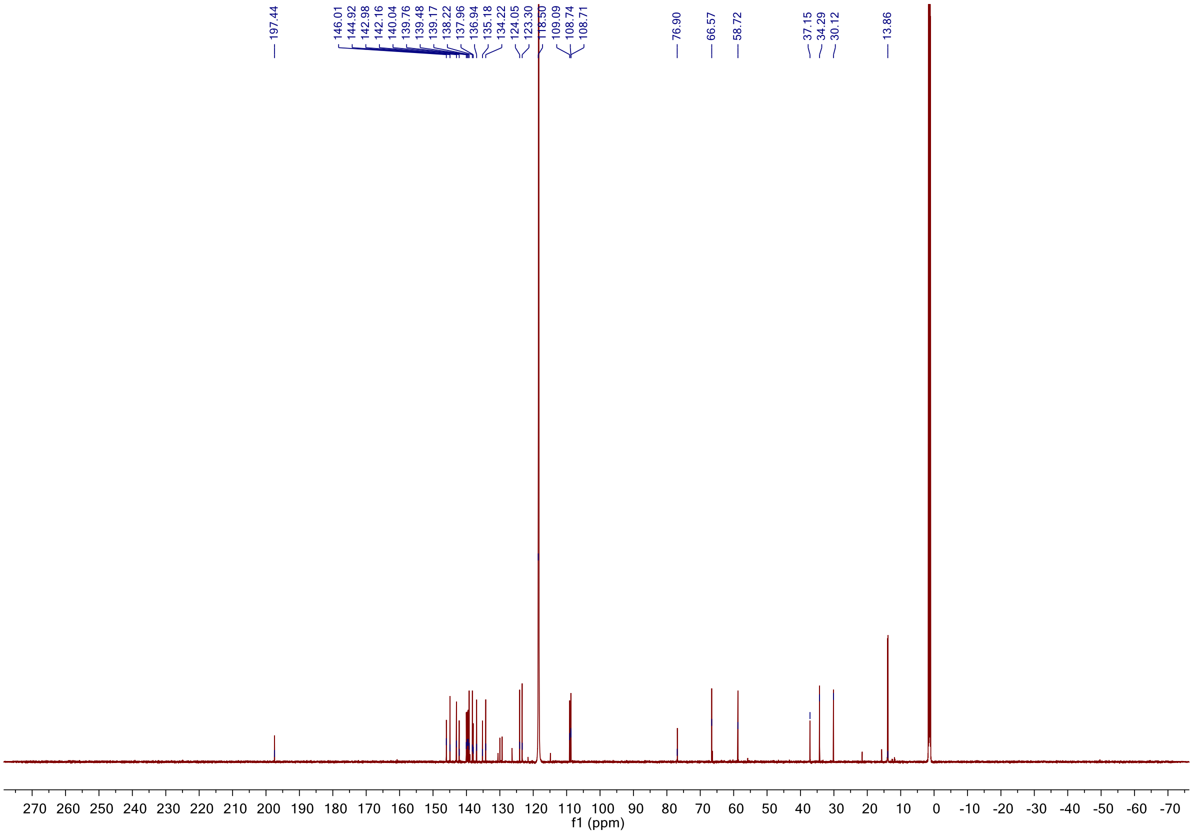

Supplementary Fig 25. Compound 28D ^{1}H NMR (800 MHz, CD_2Cl_2, δ, 25 °C) and ^{13}C NMR (800 MHz, CD_2Cl_2, δ, 25 °C)

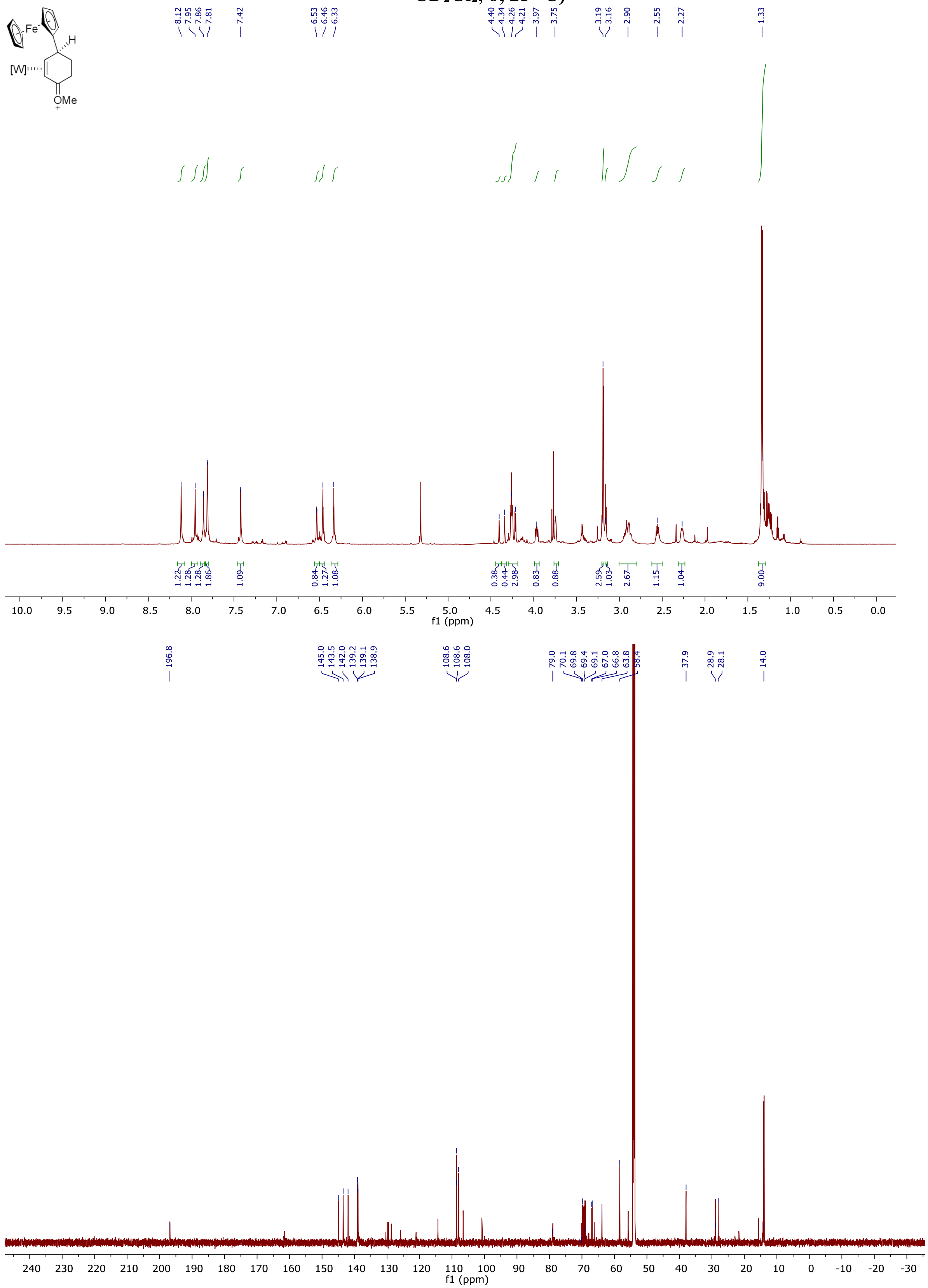

Supplementary Fig 26. Compound 29D ^{1}H NMR (600 MHz, CD_2Cl_2, δ, 25 °C) and ^{13}C NMR (800 MHz, CD_2Cl_2, δ, 25 °C)

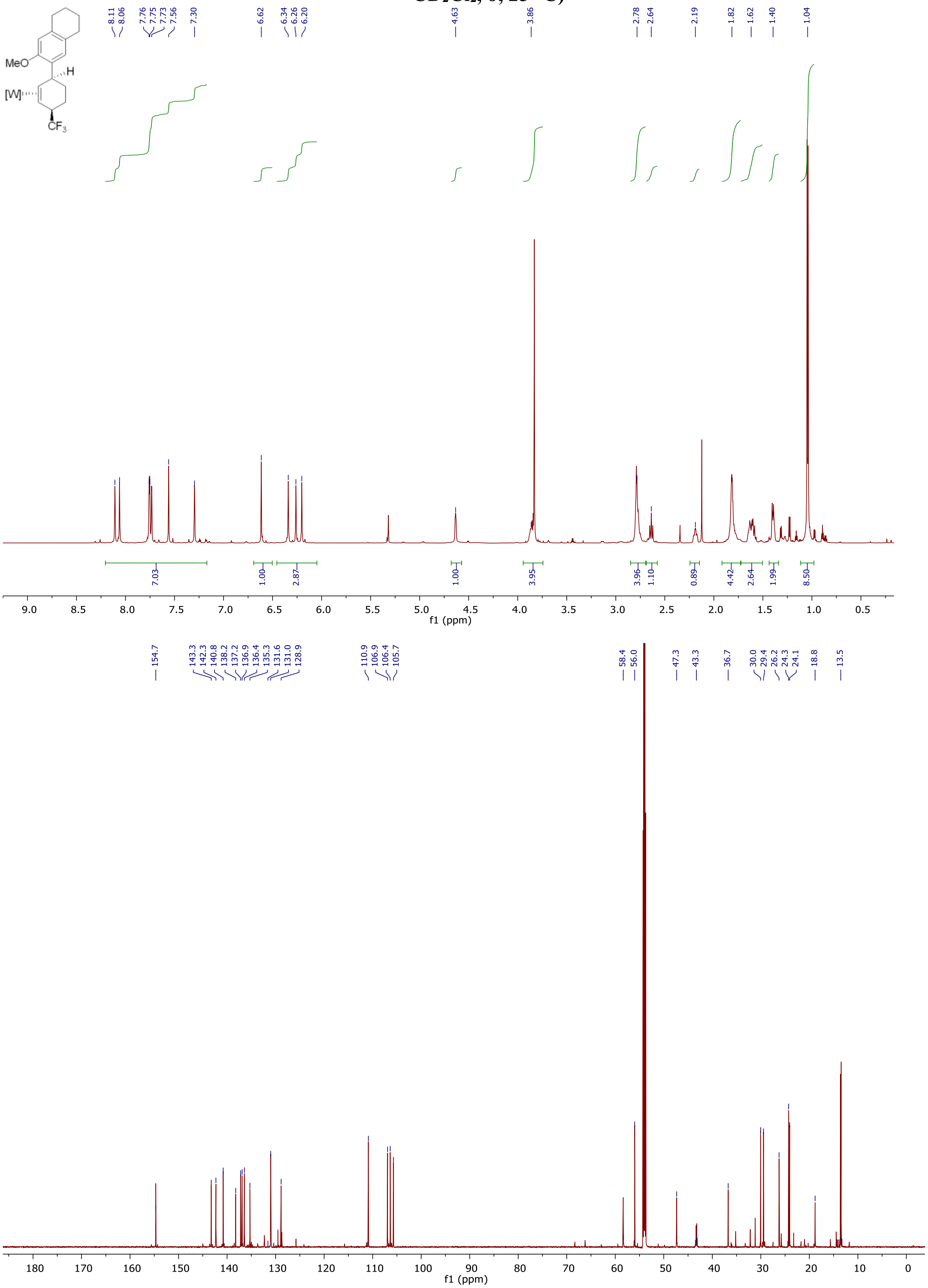

Supplementary Fig 27. Compound 30D ^{1}H NMR (800 MHz, d_6-acetone, δ, 25 °C) and ^{13}C NMR (800 MHz, d_3-acetone, δ, 25 °C)

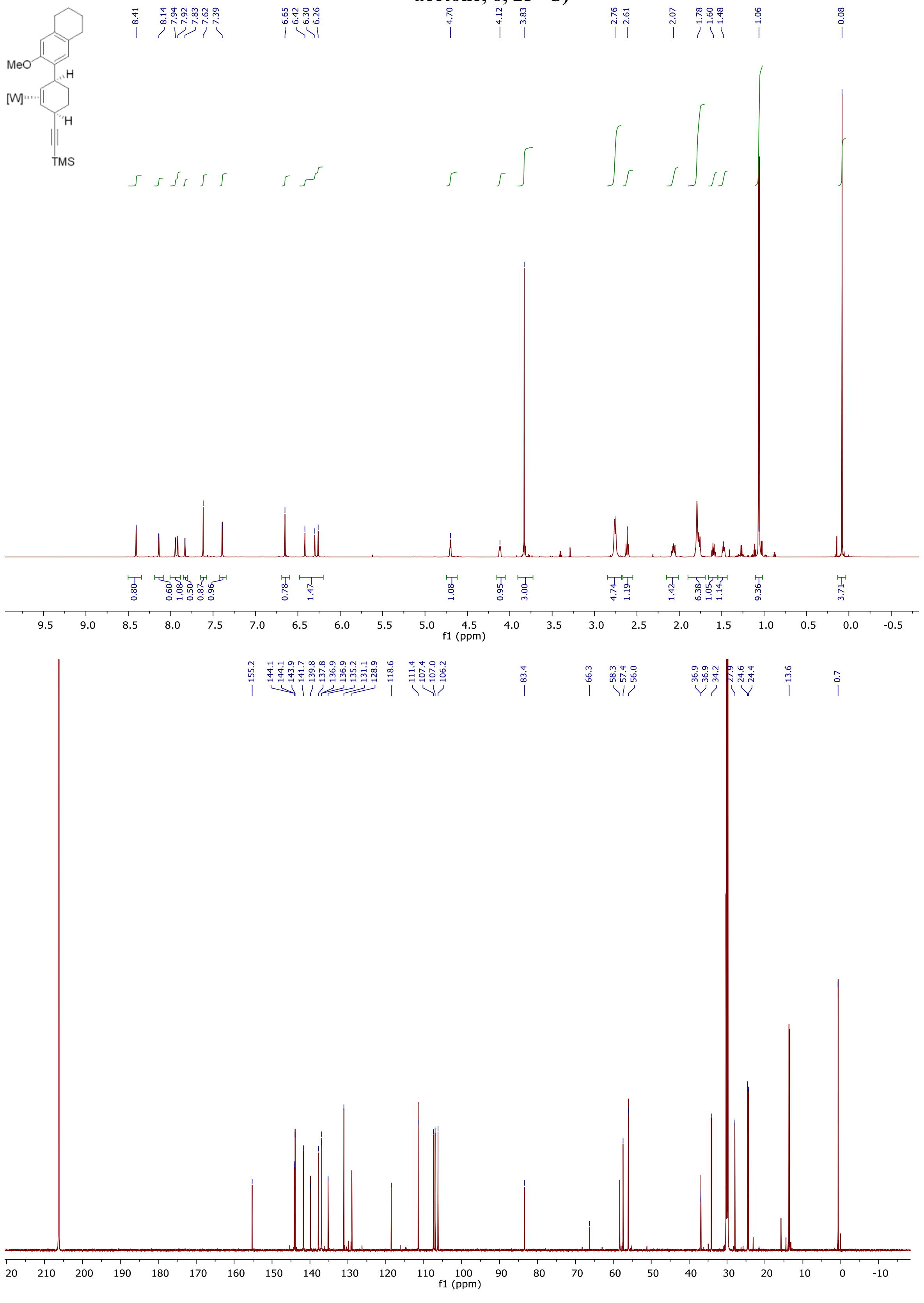

Supplementary Fig 28. Compound 31D ^{1}H NMR (800 MHz, CD_2Cl_2, δ, 25 °C) and ^{13}C NMR (800 MHz, CD_2Cl_2, δ, 25 °C)

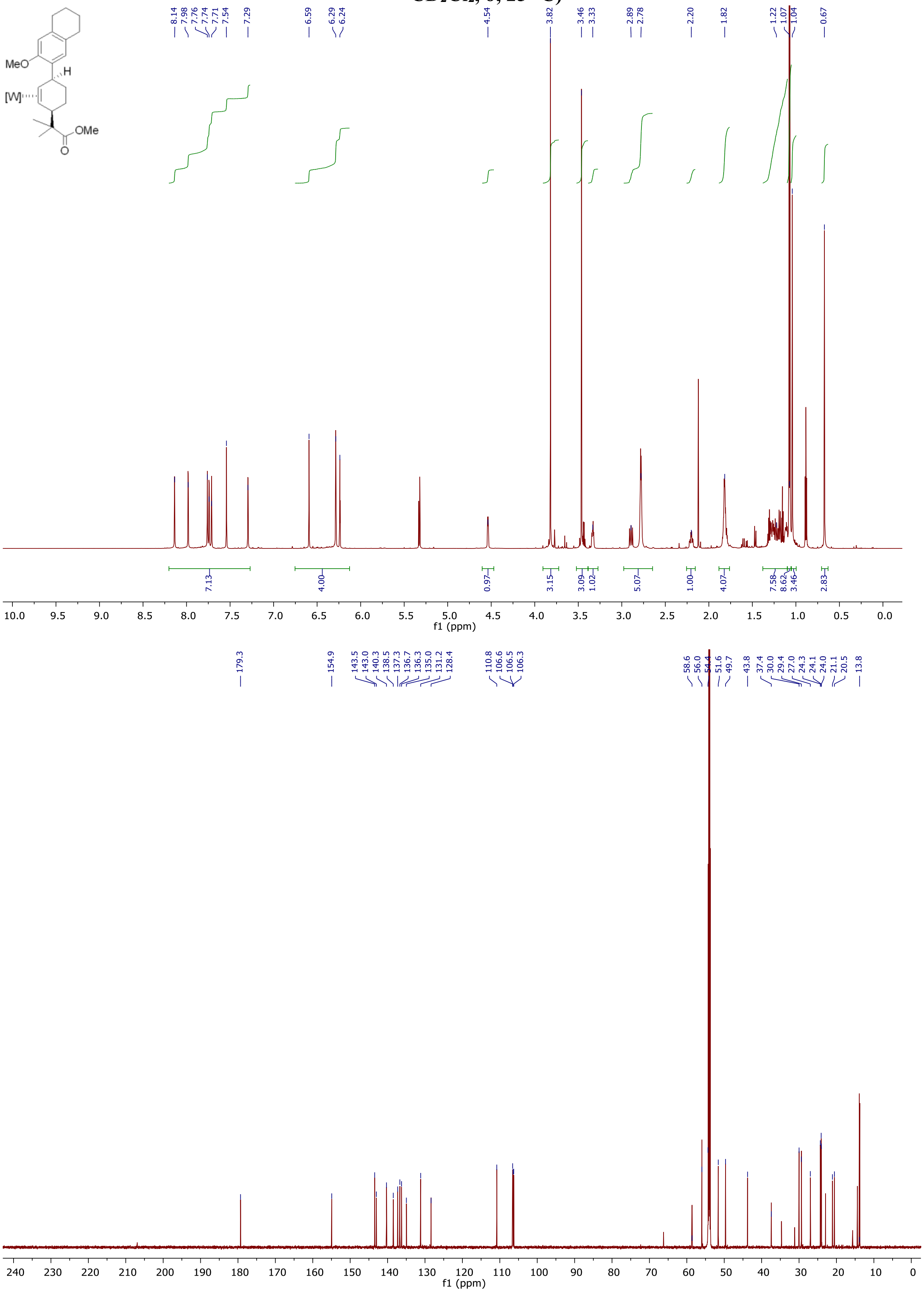

Supplementary Fig 29. Compound 32D ^{1}H NMR (800 MHz, CD_2Cl_2, δ, 25 °C) and ^{13}C NMR (800 MHz, CD_2Cl_2, δ, 25 °C)

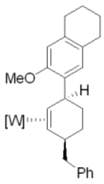

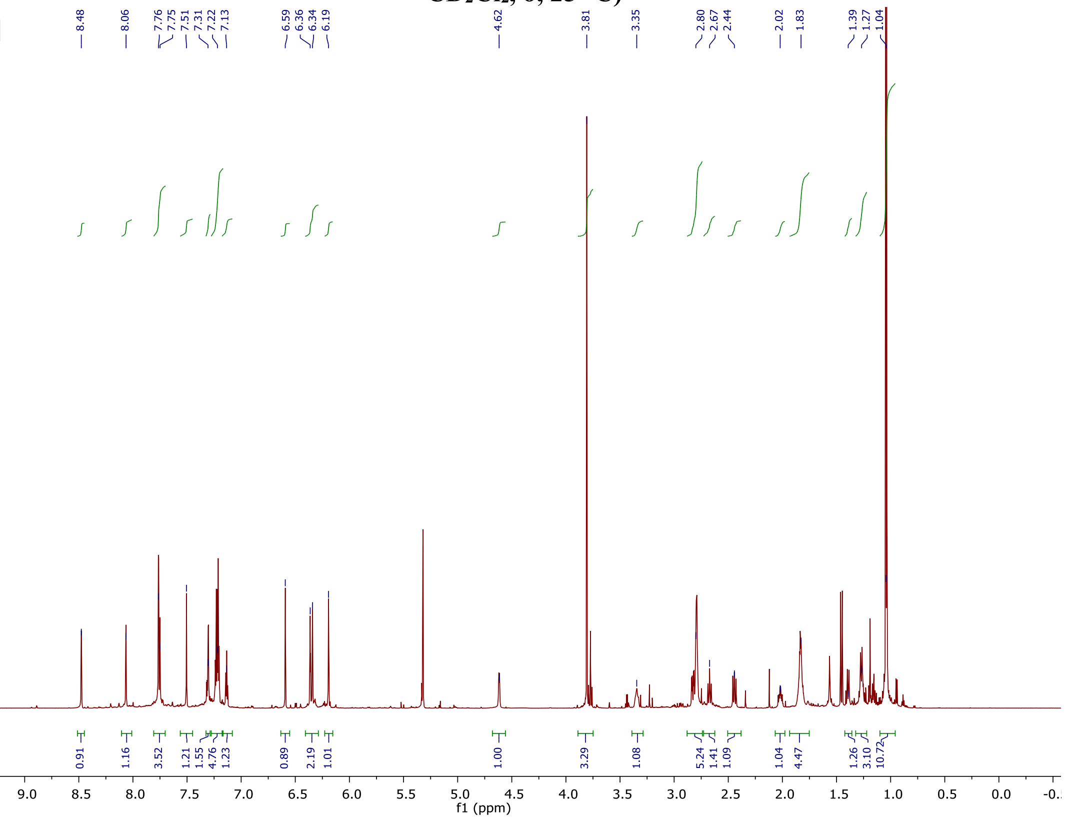

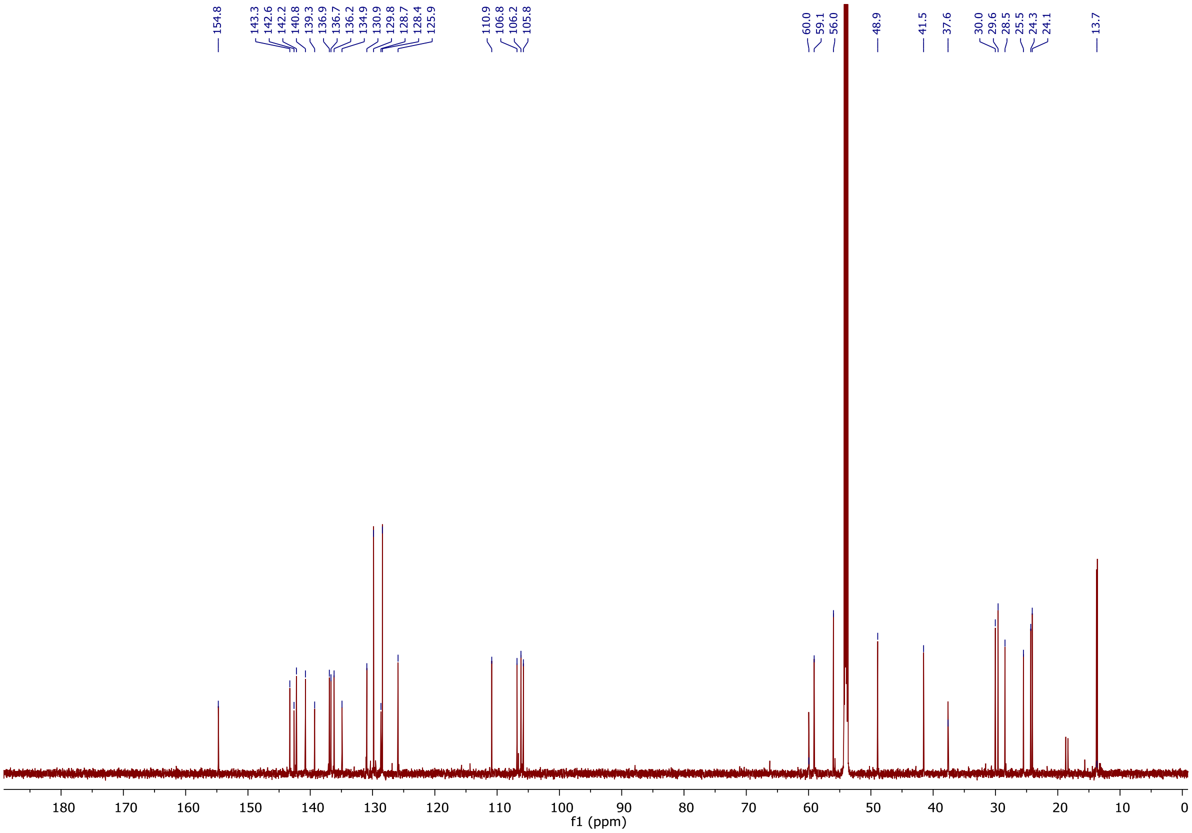

Supplementary Fig 30. Compound 33D ^{1}H NMR (800 MHz, d_3-MeCN, δ, 25 °C) and ^{13}C NMR (800 MHz, d_3-MeCN, δ, 25 °C)

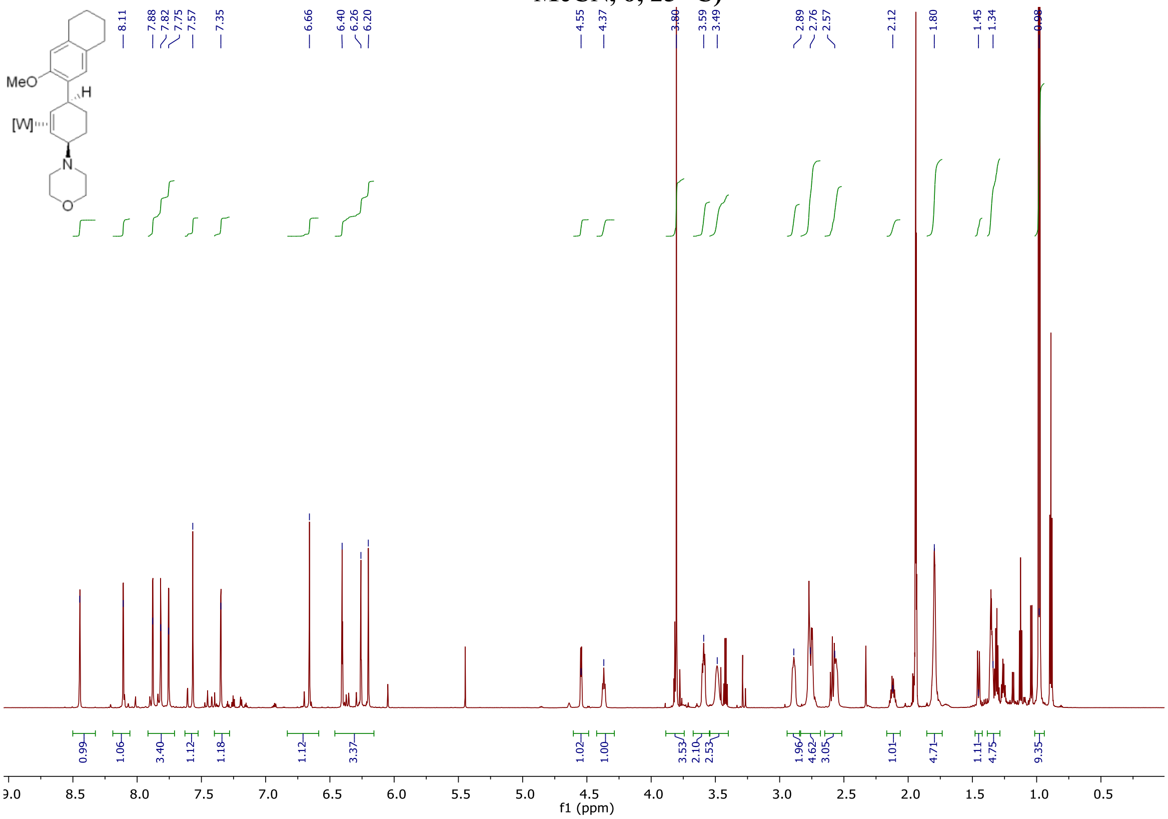

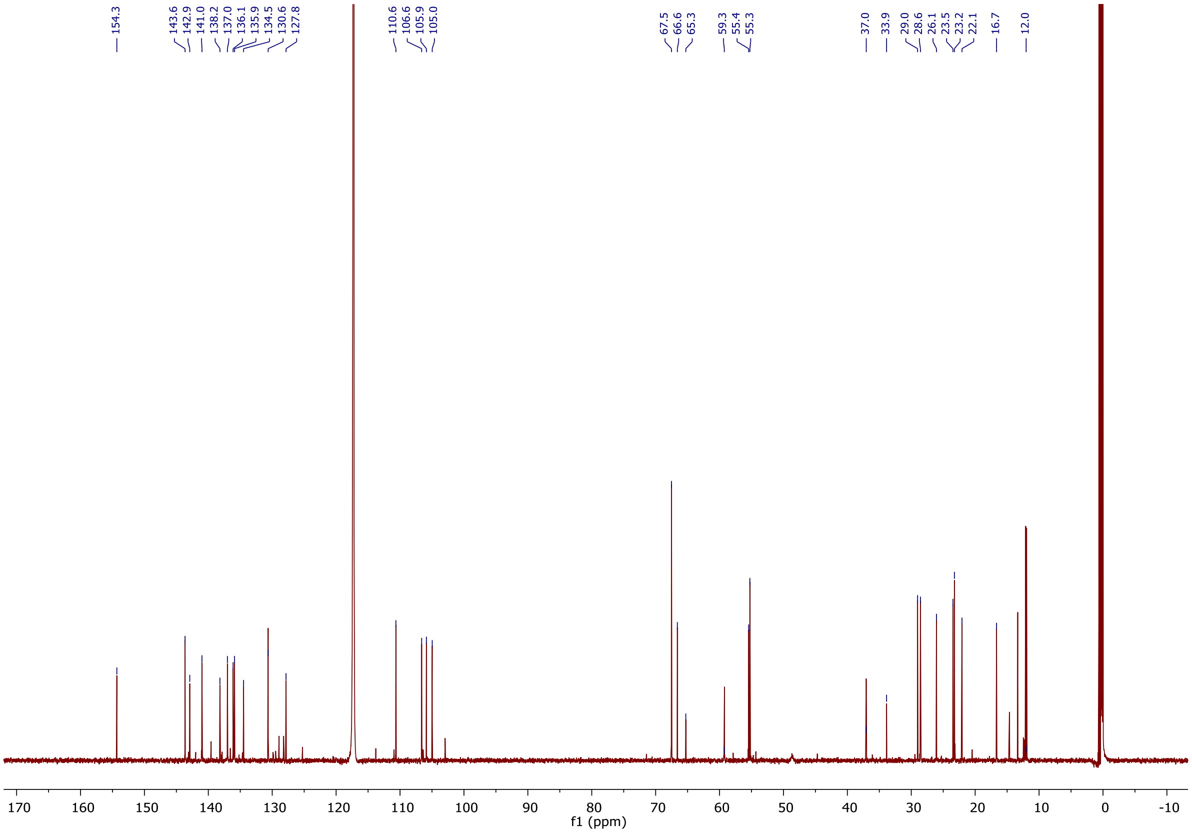

Supplementary Fig 31. Compound 34D ^{1}H NMR (800 MHz, d_3-MeCN, δ, 25 °C) and ^{13}C NMR (800 MHz, d_3-MeCN, δ, 25 °C)

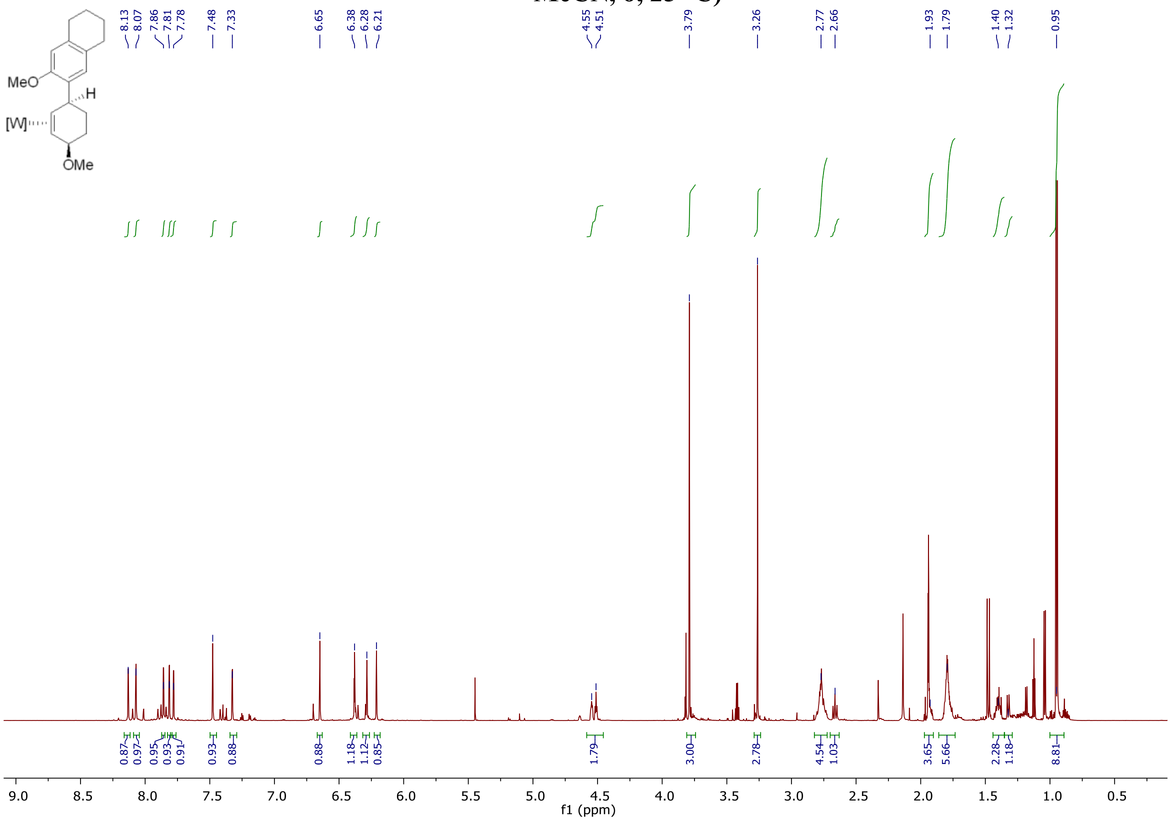

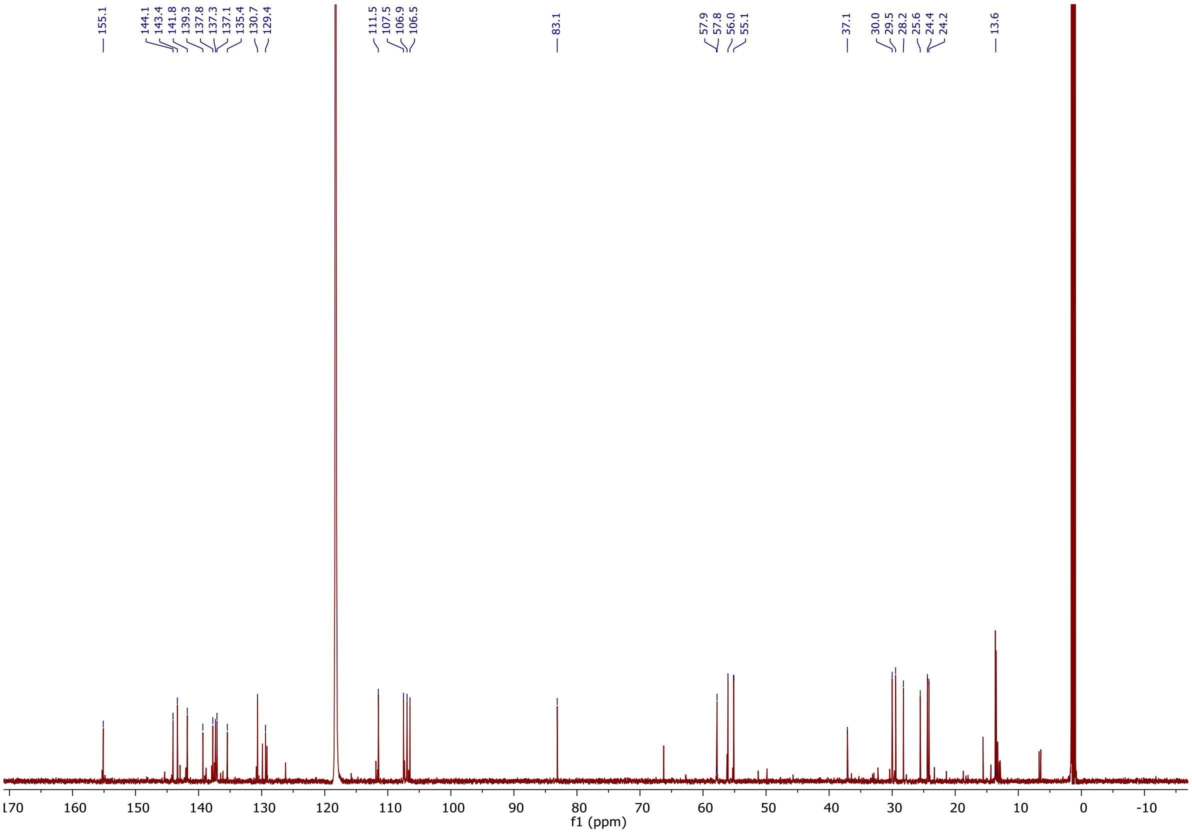

Supplementary Fig 32. Compound 35 ^{1}H NMR (800 MHz, d_6-DMSO, δ, 25 °C) and ^{13}C NMR (800 MHz, d_6-DMSO, δ, 25 °C)

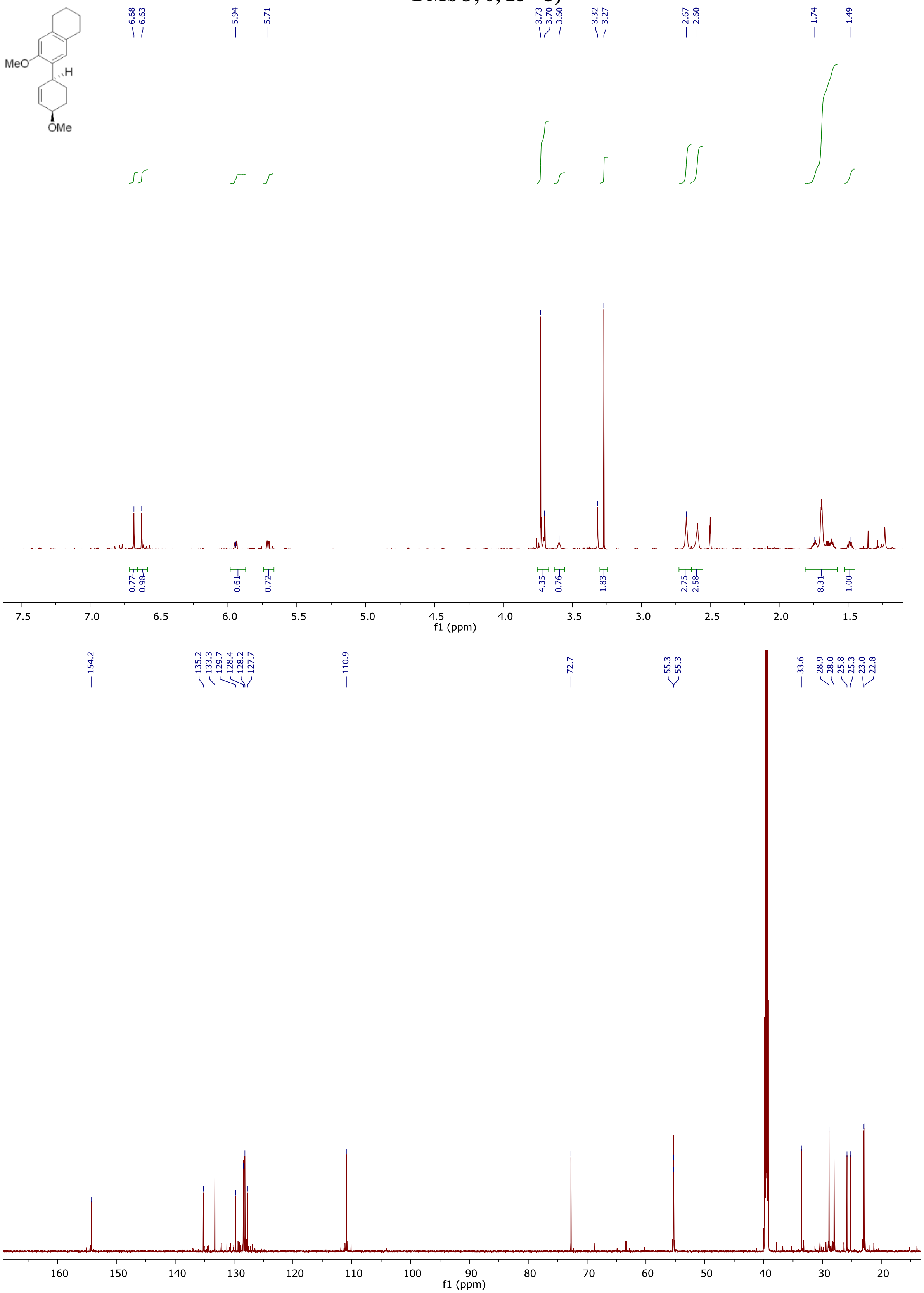

Supplementary Fig 33. Compound 36 ¹H NMR (800 MHz, *d₆*-acetone, δ, 25 °C) and ¹³C NMR (800 MHz, *d₆*-acetone, δ, 25 °C)

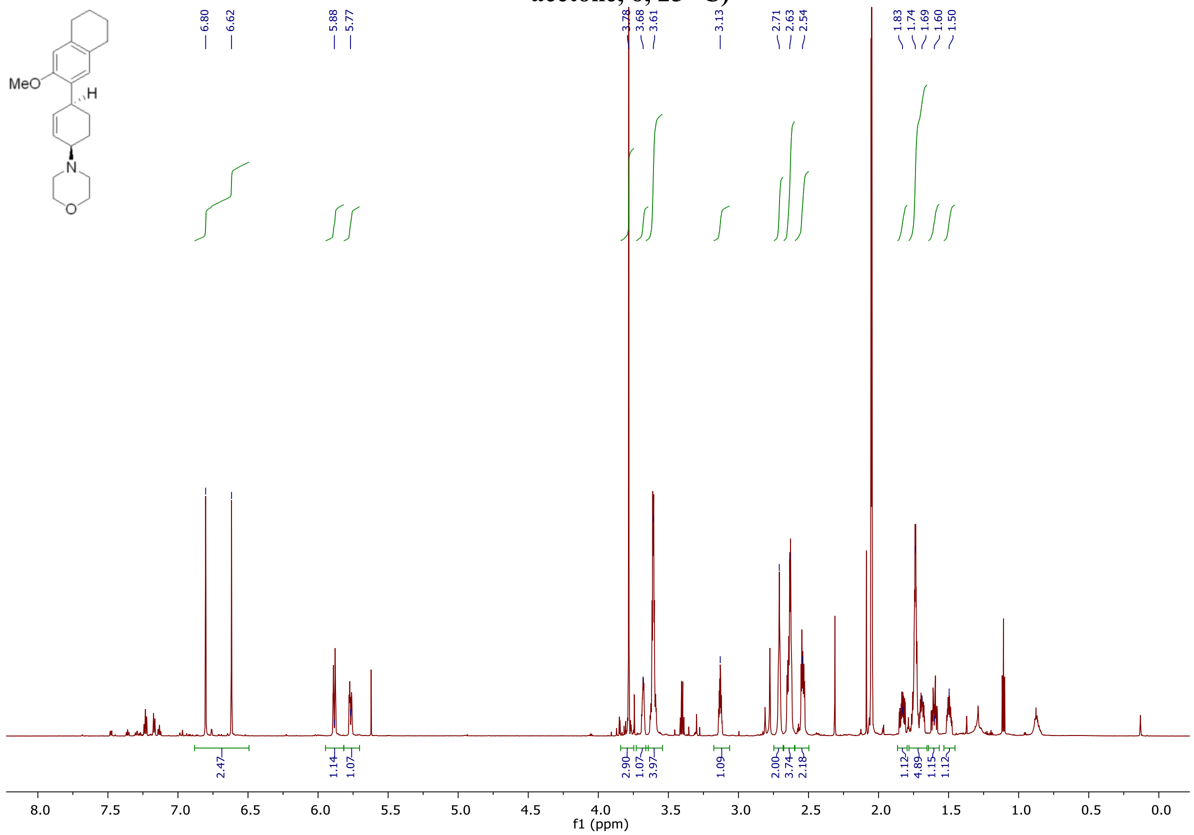

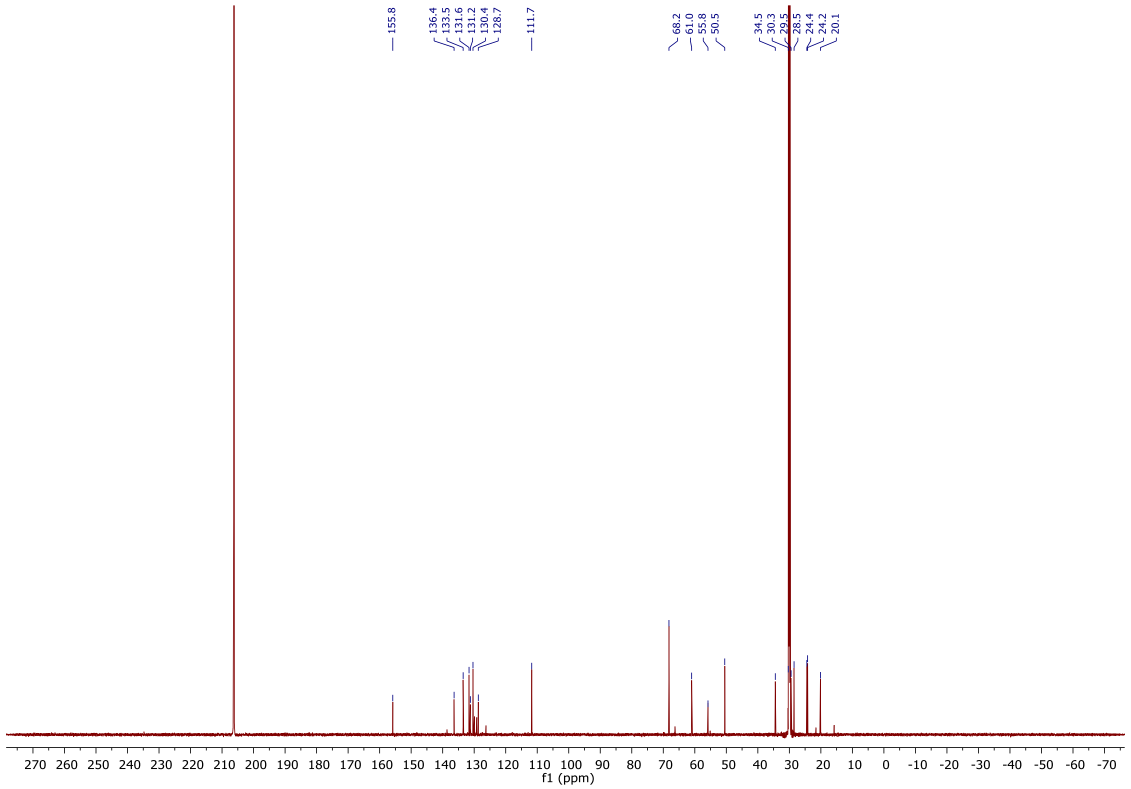

Supplementary Fig 34. Compound 37 ^{1}H NMR (800 MHz, CD_2Cl_2, δ, 25 °C) and ^{13}C NMR (800 MHz, CD_2Cl_2, δ, 25 °C)

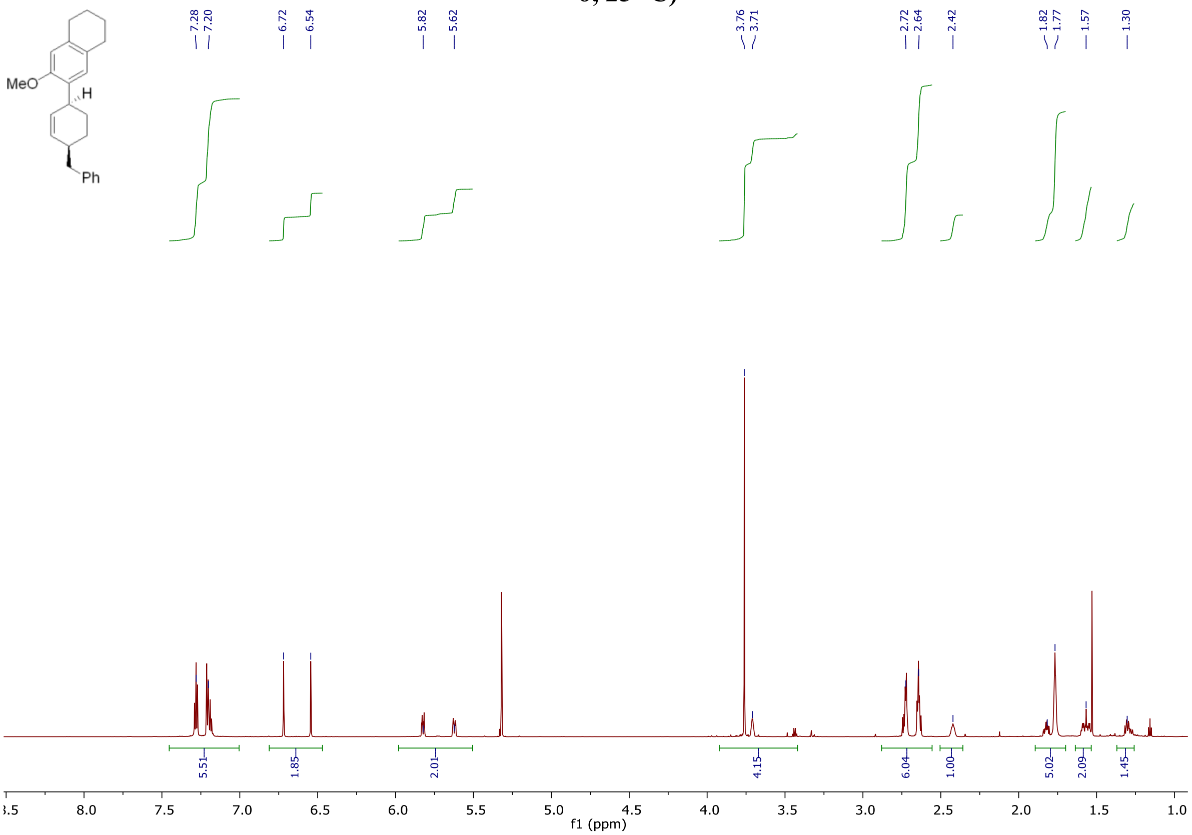

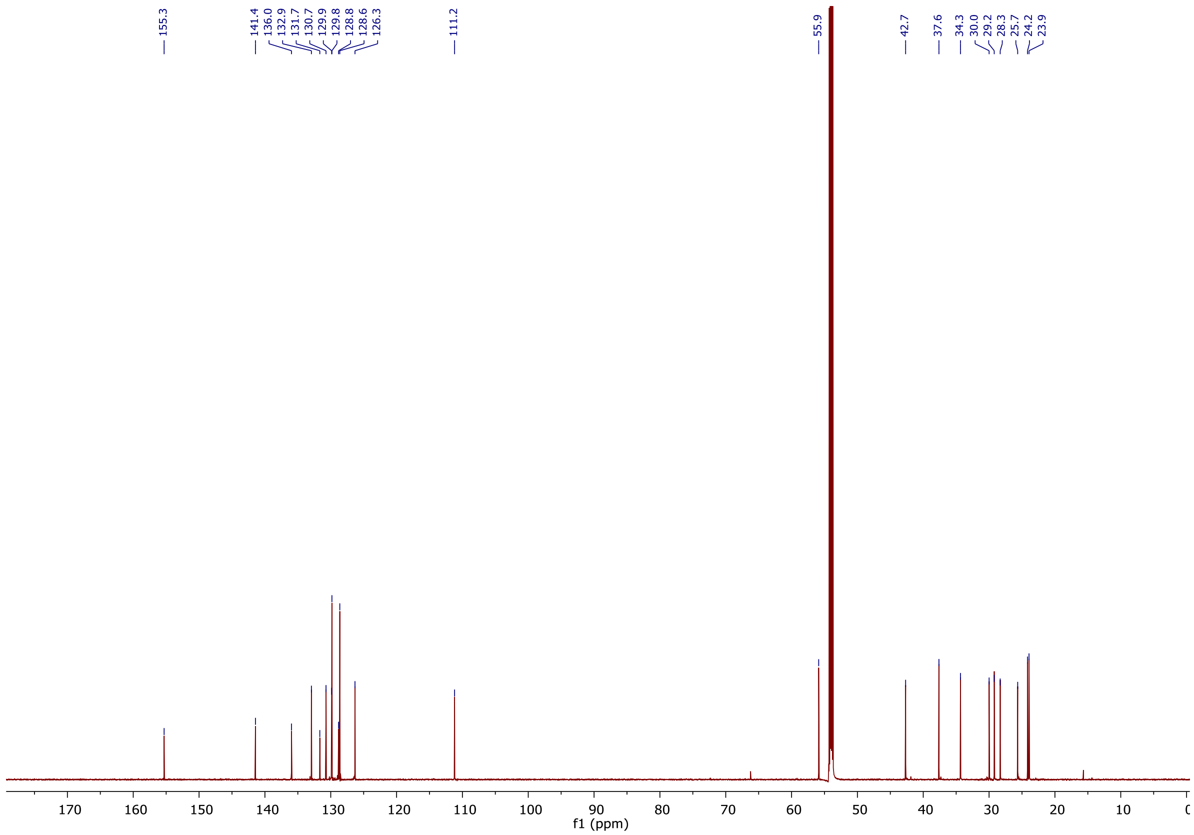
155.3
141.4
136.0
132.9
131.7
130.7
129.9
129.8
128.8
128.6
126.3
111.2
55.9
42.7
37.6
34.3
30.0
29.2
28.3
25.7
24.2
23.9
170
160
150
140
130
120
110
100
90
80
70
60
50
40
30
20
10
f1 (ppm)

Supplementary Fig 35. Compound 38 ^{1}H NMR (800 MHz, d_6-acetone, δ, 25 °C) and ^{13}C NMR (800 MHz, d_6-acetone, δ, 25 °C)

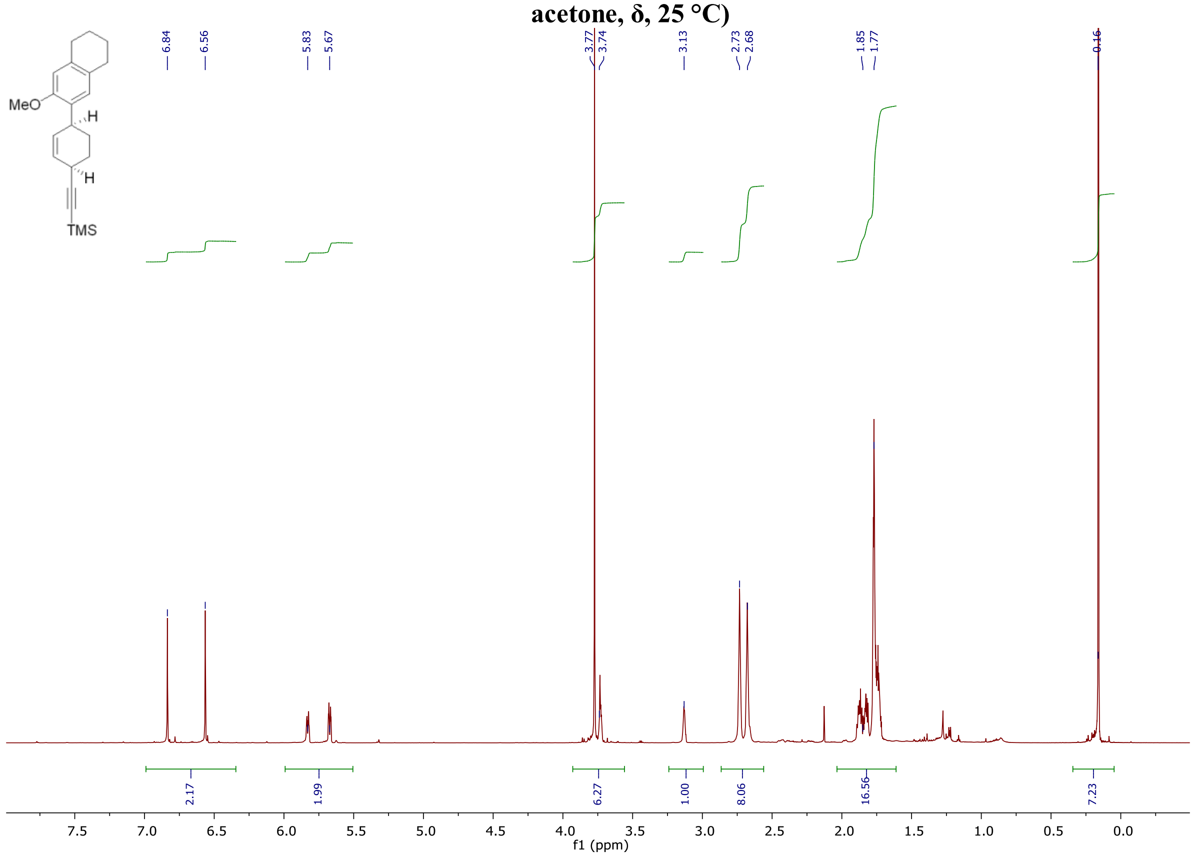

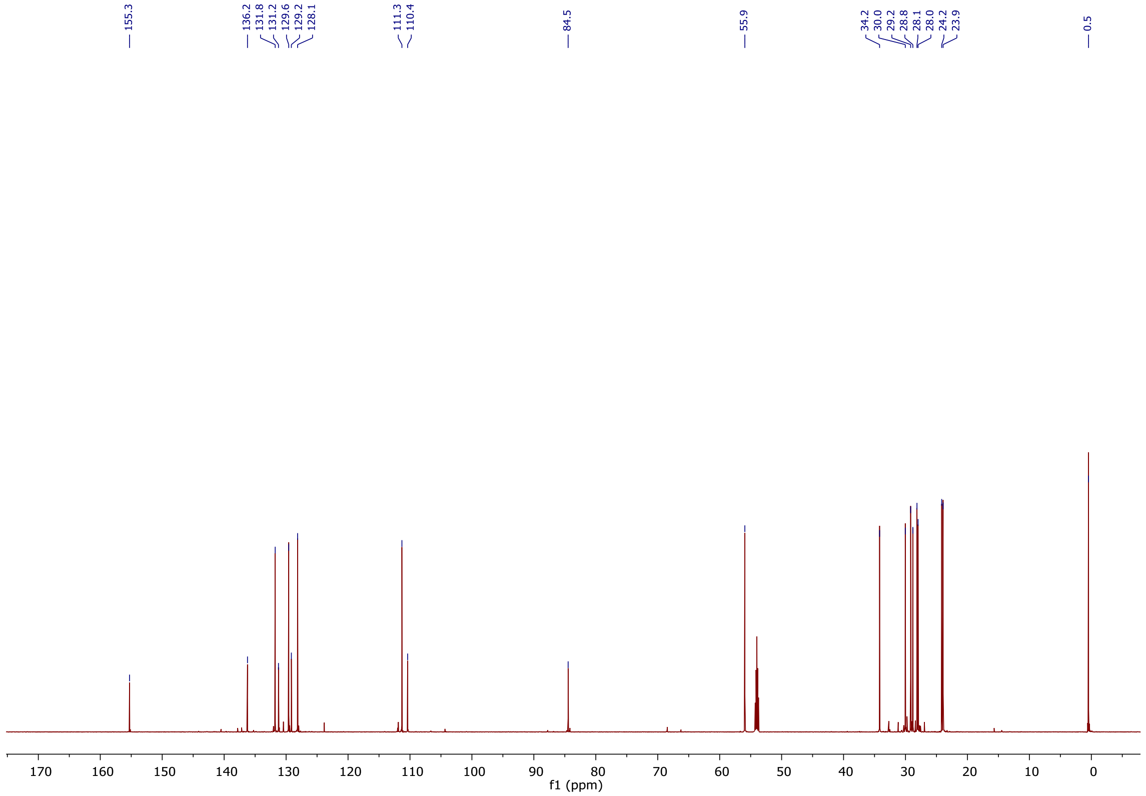

Supplementary Fig 36. Compound 39 ^{1}H NMR (800 MHz, d_6-acetone, δ, 25 °C) and ^{13}C NMR (800 MHz, d_6-acetone, δ, 25 °C)

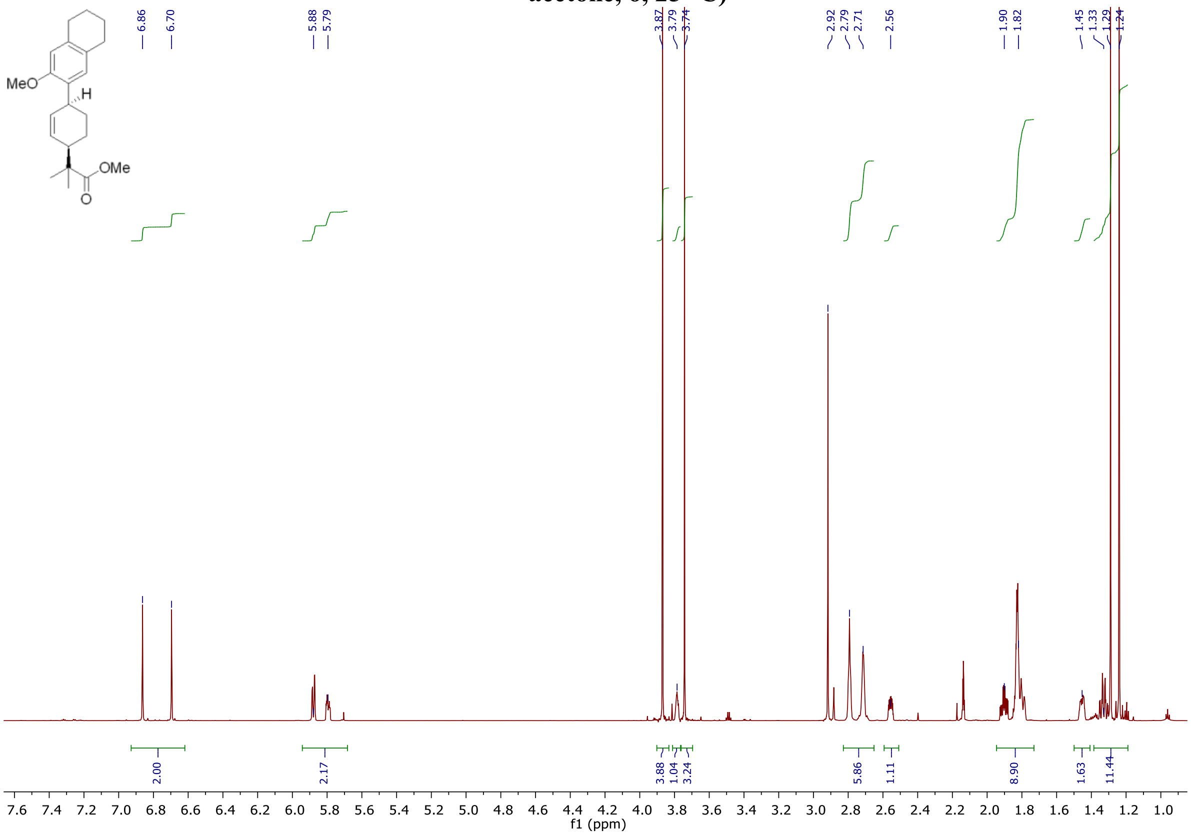

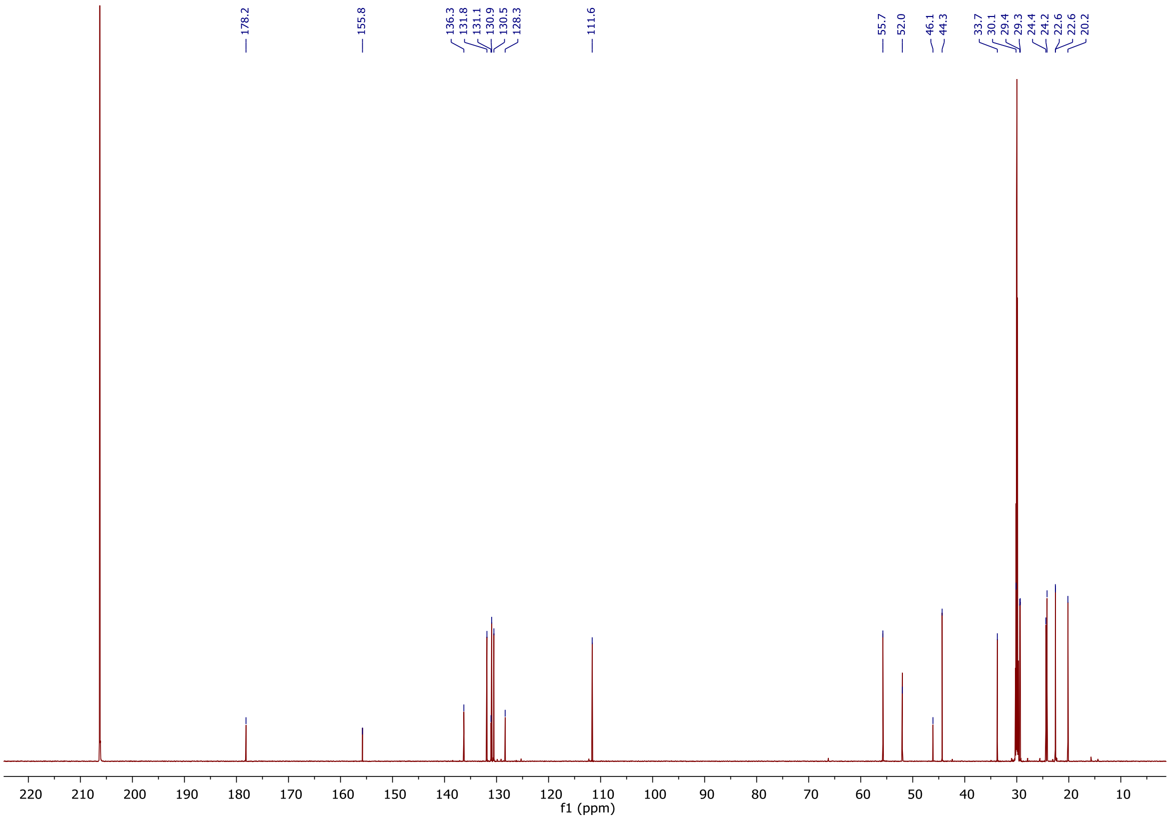

Supplementary Fig 37. Compound 40 ^{1}H NMR (800 MHz, d_6-acetone, δ, 25 °C) and ^{13}C NMR (800 MHz, d_6-acetone, δ, 25 °C)

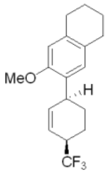

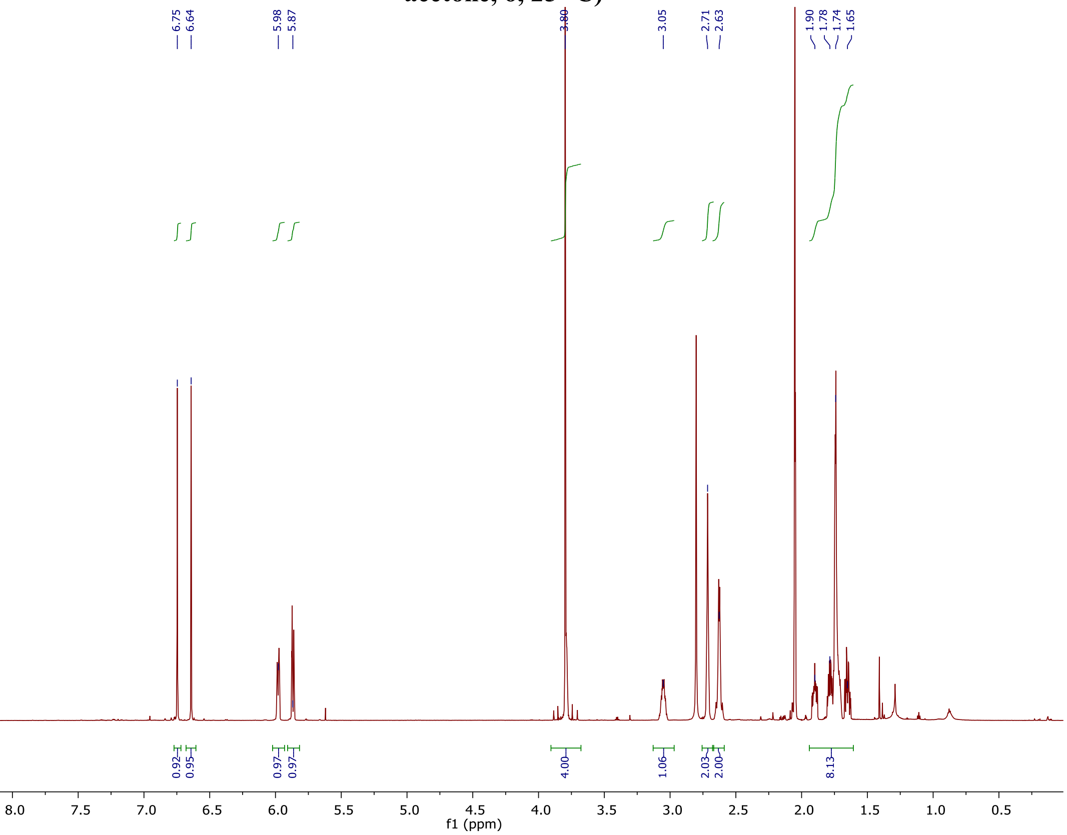

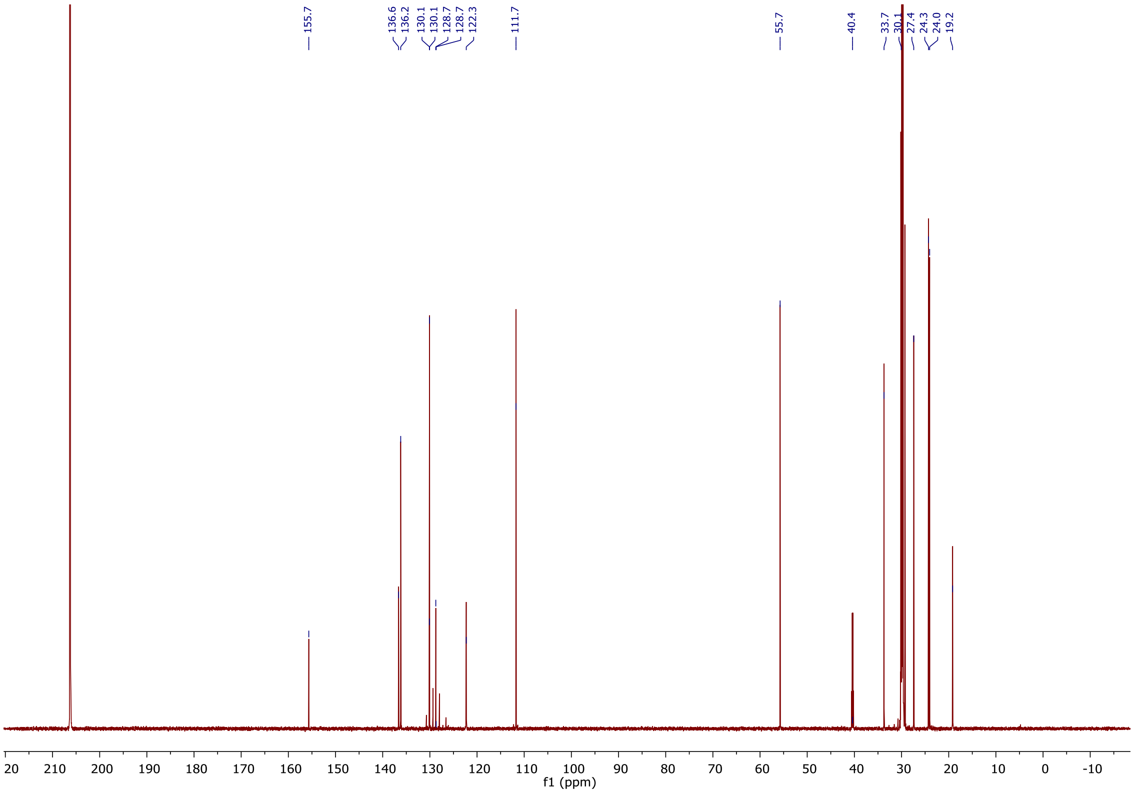

Supplementary Fig 38. Compound 41 ^{1}H NMR (800 MHz, d_3-MeCN, δ, 25 °C) and ^{13}C NMR (800 MHz, d_3-MeCN, δ, 25 °C)

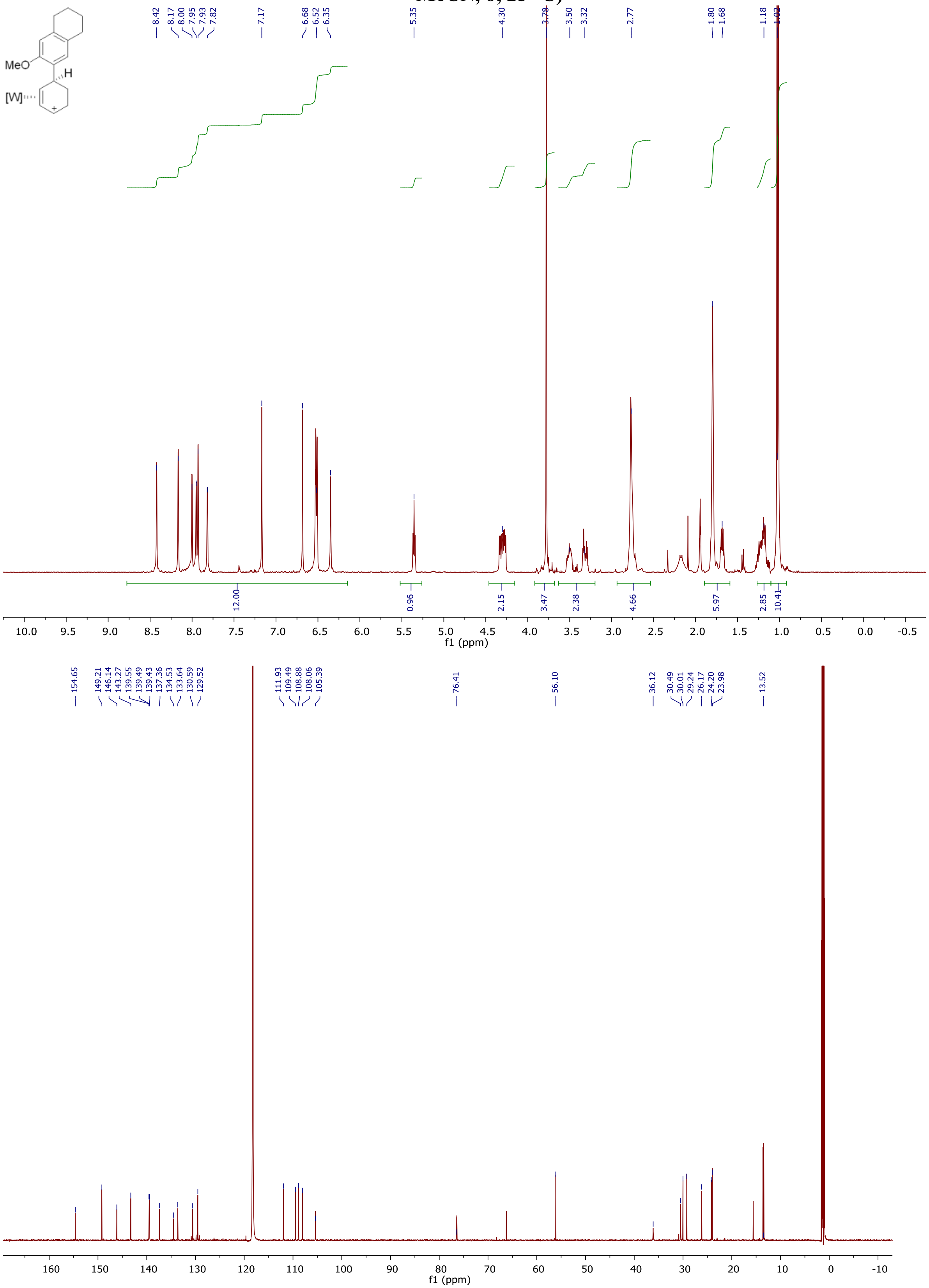

Experimental Procedures & Characterizations

General Methods: NMR spectra were obtained on 500, 600, or 800 MHz spectrometers. Chemical shifts are referenced to tetramethylsilane (TMS) utilizing residual ^{1}H or ^{13}C signals of the deuterated solvents as internal standards. Phosphorus NMR signals are referenced to 85% H_3PO_4 (δ 0.00) using a triphenyl phosphate external standard (δ −16.58). Chemical shifts are reported in ppm, and coupling constants (*J*) are reported in hertz (Hz). Infrared (IR) spectra were recorded on a spectrometer as a glaze on a diamond anvil ATR assembly, with peaks reported in cm^{-1}. Electrochemical experiments were performed under a nitrogen atmosphere. Cyclic voltametric data were recorded at ambient temperature at 100 mV/s, unless otherwise noted, with a standard three electrode cell from +1.8 to −1.8 V with a glassy carbon working electrode, tetrabutylammonium hexafluorophosphate (TBAH) electrolyte (~1.0 M), and acetonitrile (MeCN) or tetrahydrofuran (THF) as the solvent. All potentials are reported versus the normal hydrogen electrode (NHE) using cobaltocenium hexafluorophosphate ($E_{1/2}$ = −0.78, −1.75 V) or ferrocene ($E_{1/2}$ = 0.55 V) as an internal standard. Peak separation of all reversible couples was less than 100 mV. All synthetic reactions were performed in a glovebox under a dry nitrogen atmosphere unless otherwise noted. All solvents were sparged with nitrogen prior to use. Deuterated solvents were used as received from Cambridge Isotopes. When possible, pyrazole (Pz) protons of the (trispyrazolyl) borate (Tp) ligand were assigned as "Pz3/5 or Pz4". B-H peaks (around 4−5 ppm) in the ^{1}H NMR spectra are not assigned due to their quadrapole broadening; however, confirmation of the BH group is provided by IR data (around 2500 cm^{-1}). Compounds **1**, **12**, and **13D** were prepared according to previous literature procedures with some modifications.[1,2]

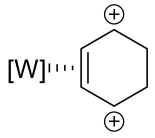

5

To an NMR tube was added **1** along with CD_2Cl_2. This homogeneous yellow reaction mixture was allowed to cool to -60 °C for 5 min before adding recently thawed HOTF (8 drops). The NMR tube was then removed from a glovebox and placed in a small dewar filled with $N_2(l)$ that froze the contents of the NMR tube solution. The reaction mixture was allowed to thaw to reveal a homogeneous red reaction mixture. The resulting NMR solution was analyzed at 0 °C in CD_2Cl_2.

^{1}H NMR (800 MHz, CD_2Cl_2, δ, 0 °C): 8.25 (1H, broad s, Tp3/5), 8.21 (buried, broad s, Tp3/5), 8.07 (1H, broad s, Tp3/5), 7.94 (1H, broad s, Tp3/5), 7.54 (1H, broad s, Tp3/5), 7.13 (1H, broad s, benzenium), 6.87 (1H, broad s, benzenium), 6.82 (1H, broad s, Tp4), 6.72 (1H, broad s, Tp4), 6.49 (1H, broad s, Tp4), 6.14 (1H, broad s, benzenium), 5.88 (1H, broad s, benzenium), 3.79 (1H, broad s, benzenium), 3.65 (1H, broad s, benzenium), 3.13 (1H, broad s, benzenium), 3.11 (1H, broad s, benzenium), 1.24 (9H, d, J_{PH} = 10.4, PMe_3). **^{13}C NMR (800 MHz, CD_2Cl_2, δ, 0 °C):** 166.0 (benzenium), 146.0 (Tp3/5), 144.8 (Tp3/5), 143.6 (Tp3/5), 141.3 (Tp3/5), 141.0 (Tp3/5), 140.8 (2C, Tp3/5 and benzenium), 110.7 (Tp4), 110.6 (Tp4), 109.0 (Tp4), 95.9 (benzenium), 86.6 (benzenium), 22.6 (benzenium), 22.1 (benzenium), 12.3 (d, J_{PC} = 33.9, PMe_3).

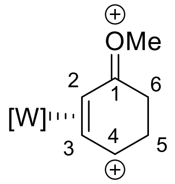

14P

To an NMR tube was added **12** and the sample was chilled to -30 °C for 5 min before adding a chilled (-30 °C) solution of HOTf (1 drop) in d_3-MeCN. This red homogenous solution was then analyzed by NMR at 25 °C and 0 °C.

^{1}H NMR (800 MHz, d_3-MeCN, δ, 0 °C): 8.34 (1H, d, J = 2.2 Hz, Tp3/5), 8.13 (1H, d, J = 2.3 Hz , Tp3/5), 8.09 (1H, d, J = 2.2 Hz, Tp3/5), 8.02 (1H, d, J = 1.9 Hz, Tp3/5), 7.90 (1H, d, J = 2.2 Hz, Tp3/5), 7.87 (1H, d, J = 2.2 Hz, Tp3/5), 6.82 (1H, m, H4), 6.63 (1H, t, J = 2.3 Hz, Tp4), 6.56 (1H, t, J = 2.4 Hz, Tp4), 6.34 (1H, t, J = 2.4 Hz, Tp4A), 5.74 (1H, , m, H3), 5.36 (1H, dd, J = 10.5, 5.5 Hz, H2), 4.62 (3H, s, OMe), 3.74 (1H, m, H5-anti), 3.52 (1H, dd, J = 21.9, 9.9 Hz, H5-syn), 3.40 (1H, dd, J = 22.6, 10.1 Hz, H6), 3.01 (1H, dd, J = 22.5, 9.7 Hz, H6'), 1.12 (9H, d, J_{PH} = 10.5 Hz, PMe_3).**^{31}P NMR (500 MHz, d_3-MeCN, δ, 25 °C):** 0.76 (J_{WP} = 248.0 Hz). **^{13}C NMR (800 MHz, d_3-MeCN, δ, 0):** 217.4 (C1), 149.4 (C4), 148.5 (Tp3/5), 145.9 (Tp3/5), 144.2 (Tp3/5, d, J_{PC} = 3.8 Hz), 140.8 (2C, overlapping Tp3/5), 140.4 (Tp3/5), 110.3 (Tp4, d, J_{PC} = 2.5), 109.8 (Tp4), 108.6 (Tp4, d, J_{PC} = 6.7 Hz), 98.7 (C3), 64.9 (2C, overlap, - OMe and C2), 27.1 (C6), 24.5 (C5), 12.6 (PMe_3, d, J_{PC} = 34.0 Hz).

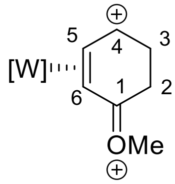

14D

To an NMR tube was added **13D** along with d_3-MeCN (~1 mL). This orange homogenous solution was chilled to -30 °C for 5 min. A drop of chilled (-30 °C) HOTf was then added to produce a homogenous red solution which was subsequently analyzed by NMR at 25 °C and 0 °C.

^{1}H NMR (600 MHz, d_3-MeCN, δ, 25 °C): 8.23 (2H, overlapping, Tp3/5), 8.20 (1H, d, J = 2.2 Hz, Tp5C), 8.08 (1H, d, J = 2.0 Hz Tp3/5), 8.05 (1H, d, J = 2.0 Hz, Tp3/5), 7.23 (1H, s, Tp3/5), 7.07 (1H, m, H4), 6.68 (1H, t, J = 2.2 Hz, Tp4), 6.58 (1H, t, J = 2.3 Hz, Tp4), 6.44 (1H, t, J = 2.3 Hz, Tp4), 6.14 (1H, m, H5), 4.36 (1H, d, J = 5.3 Hz, H6), 3.78 (1H, m, H3), 3.68 (3H, b, OMe), 3.31 (1H, dd, J = 21.1, 10.5 Hz H3'), 3.17 (1H, dd, J = 22.3, 10.6 Hz, H2), 2.68 (1H, ddd, J = 22.2, 10.2, 4.3 Hz, H2'), 1.21 (9H, d, J_{PH} = 10.4, PMe_3). **^{31}P NMR (500 MHz, d_3-MeCN, δ, 25 °C):** -0.20 (J_{WP} = 240.0 Hz). **(800 MHz, d_3-MeCN, δ, 0 °C):** 212.2 (C1), 145.5 (2C, overlapping Tp3/5), 143.0 (Tp3/5), 141.6 (Tp3/5), 141.4 (Tp3/5), 141.2 (Tp3/5), 136.4 (C4), 110.3 (2C, overlapping, Tp4), 110.0 (Tp4), 93.0 (C5), 64.1 (C6), 53.0 (OMe), 26.6 (C2), 23.9 (C3), 12.4 (PMe_3, d, J_{PC} = 34.3 Hz).

General Procedure 1: To a test tube was added **13D** and MeCN (1 mL). This solution was chilled to -30 °C for 5 min before adding a chilled (-30 °C) solution of HOTf in MeCN. A chilled (-30 °C) solution of the nucleophile in MeCN (1 mL) was then added. The reaction was monitored by

^{31}P NMR and determined to be complete based on the disappearance of **14D** and the formation of a new signal with J_{WP} ~285 Hz. The reaction mixture was then diluted with DCM (5 mL) and washed with DI H_2O (3 x 5 mL). The organic layer was dried over anhydrous $MgSO_4$ and concentrated in vacuo. The film was dissolved in minimal DCM and added to stirring Et_2O (300 mL). The resulting precipitate was collected on a fine porosity frit and washed with Et_2O (30 mL).

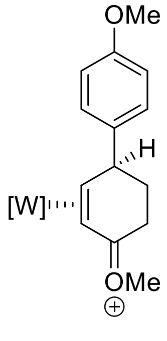

15D

Used General Procedure 1 with **13D** (1.015 g, 1.33 mmol), HOTf in MeCN (2 mL, 7.83 mmol), anisole in MeCN (3.17 mL, 20.0 mmol). Orange-tan solid (0.880 g (76%)).

^{1}H NMR (800 MHz, *d$_6$*-acetone, δ, 25 °C): 8.37 (1H, d, *J* = 1.9 Hz), 8.21 (1H, d, *J* = 2.2 Hz), 8.16 (1H, d, *J* = 2.0 Hz), 8.14 (1H, d, *J* = 2.3 Hz), 8.11 (1H, d, *J* = 2.4 Hz), 7.62 (1H, d, *J* = 1.9 Hz), 7.49 (2H, d, *J* = 8.5 Hz), 6.97 (2H, d, *J* = 8.6 Hz), 6.58 (1H, t, *J* = 2.3 Hz), 6.57 (1H, t, *J* = 2.3 Hz), 6.48 (1H, t, *J* = 2.3 Hz), 4.36 (2H, overlapping), 3.81 (3H, s), 3.61 (1H, d, *J* = 8.0), 3.25 (3H, s), 3.13 (1H, ddd, *J* = 17.2, 10.6, 6.0 Hz), 2.84 (1H, dt, J = 18.3, 4.1 Hz), 2.26 (1H, m), 1.80 (1H, m), 1.21 (9H, d, J_{PH} = 9.6). **^{31}P NMR (500 MHz, *d$_3$*-MeCN, δ, 25 °C):** -9.43 (J_{WP} = 284 Hz). **^{13}C NMR (800 MHz, *d$_6$*-acetone, δ, 25 °C):** 196.3, 159.6, 146.2, 144.9, 143.1, 142.4, 140.0, 139.7, 139.5, 129.5, 115.2, 109.1, 108.9, 108.7, 76.1 (d, J_{PC} = 15.0 Hz), 66.7, 58.4, 55.7, 44.4, 35.4, 30.1, 14.0 (d, J_{CP} = 31.6 Hz). **HRMS (ESI)** calculated for M+: 718.2197, observed 718.2201. **CV (MeCN):** $E_{p,c}$ = - 1.26 V (NHE). **IR (ATR, cm^{-1}):** ν(BH) = 2518 cm^{-1}, ν(NO) = 1600 cm^{-1}.

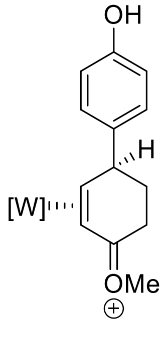

16D

Used General Procedure 1 with **13D** (0.5773 g, 0.7584 mmol), HOTf in MeCN (2 mL, 2.475 mmol), phenol in MeCN (2 mL, 3.42 mmol). Orange-tan solid (0.5049 g (77.8%)).

^{1}H NMR (800 MHz, *d$_6$*-acetone, δ, 25 °C): 8.38 (1H, d, *J* = 2.0 Hz), 8.22 (1H, d, *J* = 2.2 Hz), 8.18 (1H, d, *J* = 2.1 Hz), 8.15 (1H, d, *J* = 2.3 Hz), 8.13 (1H, d, *J* = 2.4 Hz), 7.62 (1H, d, *J* = 2.0 Hz), 7.39 (2H, d, *J* = 8.5 Hz), 6.91(2H, d, *J* = 8.5 Hz), 6.60 (1H, t, *J* = 2.3 Hz), 6.58 (1H, t, *J* = 2.3

Hz), 6.49 (1H, t, J = 2.3 Hz), 4.35 (2H, overlapping), 3.61 (1H, d, J = 8.0), 3.26 (3H, s), 3.14 (1H, ddd, J = 17.0, 10.7, 5.8 Hz), 2.85 (1H, dt, J = 18.2, 3.8 Hz), 2.25 (1H, m), 1.81 (1H, m), 1.22 (9H, d, J_{PH} = 9.6). **^{31}P NMR (500 MHz, d_3-MeCN, δ, 25 °C):** -9.34 (J_{WP} = 285 Hz). **^{13}C NMR (800 MHz, d_6-acetone, δ, 25 °C):** 196.3, 157.0, 146.2, 144.8, 143.1, 141.0, 139.9, 139.6, 139.4, 129.4, 116.5, 109.0, 108.8, 108.6, 76.3 (d, J_{PC} = 16.1 Hz), 66.7, 58.3, 44.4, 35.4, 29.8, 14.0 (d, J_{CP} = 31.3 Hz). **HRMS (ESI)** calculated for M+: 704.2041, observed 704.2045. **CV (MeCN):** $E_{1/2}$ = - 1.17 V (NHE). **IR (ATR, cm^{-1}):** ν(BH) = 2523 cm^{-1}, ν(NO) = 1609 cm^{-1}.

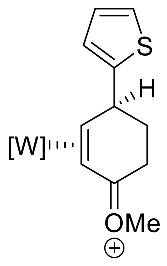

17D

Used General Procedure 1 with **13D** (0.5995 g, 0.7876 mmol), HOTf in MeCN (2 mL, 2.251 mmol), thiophene in MeCN (2 mL, 5.051 mmol). Orange-tan solid (0.4914 g (73.8%)).

^{1}H NMR (800 MHz, d_6-acetone, δ, 25 °C): 8.37 (1H, d, J = 2.2 Hz), 8.23 (1H, d, J = 2.3 Hz), 8.22 (1H, d, J = 2.3 Hz), 8.14 (1H, d, J = 2.4 Hz), 8.11 (1H, d, J = 2.5 Hz), 7.63 (1H, d, J = 2.2 Hz), 7.37 (1H, dd, J = 5.1, 1.2 Hz), 7.20 (1H, ddd, J = 3.5, 1.2, 0.7 Hz), 7.04 (1H, dd, J = 5.1, 3.5 Hz), 6.60 (1H, t, J = 2.3 Hz), 6.57 (1H, t, J = 2.3 Hz), 6.48 (1H, t, J = 2.3 Hz), 4.59 (1H, td, J = 6.0, 2.2 Hz), 4.34 (1H, ddd, J = 14.1, 8.1, 2.3 Hz), 3.58 (1H, d, J = 8.1 Hz), 3.39 (3H, s), 3.00 (1H, dt, J = 19.8, 6.6 Hz), 2.92 (1H, dt, J = 19.7, 6.7 Hz), 2.54 (1H, m), 1.96 (1H, m), 2.91 (1H, m), 1.35 (9H, d, J_{PH} = 9.5 Hz). **^{31}P NMR (500 MHz, d_3-MeCN, δ, 25 °C):** -9.2 (J_{WP} = 280 Hz). **^{13}C NMR (800 MHz, d_6-acetone, δ, 25 °C):** 198.3, 154.9, 146.1, 144.7, 143.2, 139.9, 139.7, 139.6, 127.9, 125.0, 124.7, 109.1, 108.8, 108.6, 77.0 (d, J_{PC} = 16.1 Hz), 64.9, 58.8, 40.0, 31.8, 28.8, 13.8 (d, J_{CP} = 31.5 Hz). **HRMS (ESI)** calculated for M+: 694.1655, observed 694.1659. **CV (MeCN):** $E_{p,c}$= - 1.37 V (NHE). **IR (ATR, cm^{-1}):** ν(BH) = 2521 cm^{-1}, ν(NO) = 1612 cm^{-1}.

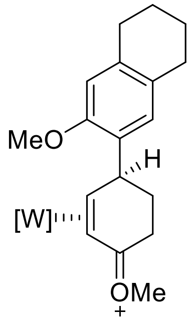

24D

Used General Procedure 1 with **13D** (0.7963 g, 1.046 mmol), HOTf (1.1321 g, 7.543 mmol) in MeCN (1 mL), and 6-methoxy-1,2,3,4-tetrahydronaphthalene (1.6545 g, 9.3894 mmol) in MeCN (1 mL). Orange-tan solid (0.6112 g (63.27%)).

^{1}H NMR (800 MHz, CD_2Cl_2, δ, 25 °C): 8.12 (1H, d, *J* = 2.2 Hz), 7.97 (1H, d, *J* = 2.3 Hz), 7.87 (1H, d, *J* = 2.5 Hz), 7.85 (1H, d, *J* = 2.5 Hz), 7.69 (1H, d, *J* = 2.3 Hz), 7.46 (1H, d, *J* = 2.1 Hz), 7.14 (1H, s), 6.63 (1H, s), 6.54 (1H, t, *J* = 2.3 Hz), 6.46 (1H, t, *J* = 2.3 Hz), 6.36 (1H, t, *J* = 2.4 Hz), 4.81 (1H, ddd, *J* = 9.3, 6.3, 2.3 Hz), 4.06 (1H, ddd, *J* = 14.9, 8.1, 2.3 Hz), 3.82 (3H, s), 3.33 (1H, d, *J* = 8.1 Hz), 3.12 (1H, m), 3.10 (3H, s), 2.78 (4H, overlapping), 2.72 (1H, m), 2.15 (1H, m), 1.80 (4H, overlapping), 1.73 (1H, m), 1.10 (9H, d, *J* = 9.4 Hz). **^{13}C NMR (800 MHz, CD_2Cl_2, δ, 25 °C):** 194.9, 154.3, 145.1, 144.1, 141.5, 139.4, 139.1, 139.0, 137.2, 134.5, 130.5, 128.7, 111.5, 108.6 (2C), 108.1, 76.8 (d, J_{CP} = 14.9 Hz), 66.2, 58.1, 55.9, 36.0, 33.8, 30.2, 30.0, 29.2, 24.0, 23.8, 14.1 (d, J_{CP} = 31.4 Hz). Composition confirmed by single crystal X-ray diffraction.

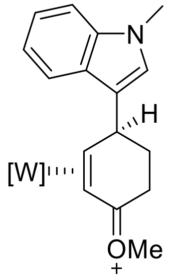

25D

Used General Procedure 1 with **13D** (0.1523 g, 0.2001 mmol), HOTf (0.1117 g, 0.7443 mmol) in MeCN (1 mL), and N-methylindole (0.5105 g, 3.8919 mmol) in MeCN (1 mL). Orange-tan solid (0.0810 g (45.4%)).

^{1}H NMR (800 MHz, *d_3*-MeCN, δ, 25 °C): 8.19 (1H, d, *J* = 2.2 Hz), 8.04 (1H, d, *J* = 2.4 Hz), 7.96 (1H, m), 7.94 (1H, d, *J* = 2.5 Hz), 7.84 (1H, d, *J* = 2.3 Hz), 7.81 (1H, dt, *J* = 8.0, 1.0 Hz), 7.53 (1H, d, *J* = 2.2 Hz), 7.41 (1H, dd, *J* = 8.2, 1.0 Hz), 7.27 (1H, s), 7.25 (1H, ddd, *J* = 8.2, 7.0, 1.1 Hz), 7.12 (1H, ddd, *J* = 8.0, 7.0, 1.0 Hz), 6.50 (1H, t, *J* = 2.3 Hz), 6.48 (1H, t, *J* = 2.3 Hz), 6.39 (1H, t, *J* = 2.3 Hz), 4.55 (1H, ddd, *J* = 8.0, 6.0, 2.3 Hz), 4.30 (1H, ddd, *J* = 14.5, 8.1, 2.3 Hz), 3.80 (3H, s), 3.42 (1H, d, *J* = 7.0 Hz), 3.19 (3H s), 2.96 (1H, dt, *J* = 19.1, 7.1 Hz), 2.68 (1H, m), 2.35 (1H, m), 2.02 (1H, dtd, *J* = 14.0, 7.8, 6.5 Hz), 1.14 (9H, d, *J* = 9.6 Hz). **^{31}P NMR (500 MHz, *d_3*-MeCN, δ, 25 °C):** -4.68 (J_{WP} = 286.5 Hz). **ESI-HRMS (m/z):** [M]+ calculated for $C_{28}H_{37}BN_8O_2PW^+$ 743.2379; found, 743.2379.

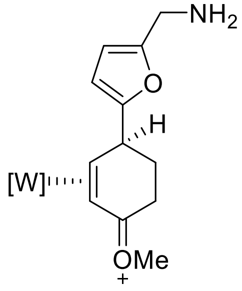

26D

Used General Procedure 2 with **13D** (0.1053 g, 0.1383 mmol), HOTf (0.1121 g, 0.7469 mmol) in MeCN (1 mL), furfurylamine (0.2631g, 2.7093 mmol) in MeCN (1 mL), and a separate solution

of HOTf (0.5776 g, 3.849 mmol) in MeCN (1 mL) to protonate the nucleophile. Orange-tan solid (0.0459 g (32.9%)).

^{1}H NMR (800 MHz, CD_2Cl_2, δ, 25 °C): 8.12 (1H, d, J = 2.3 Hz), 8.09 (1H, d, J = 2.2 Hz), 7.91 (1H, d, J = 2.3 Hz), 7.85 (1H, d, J = 2.5 Hz), 7.80 (1H, d, J = 2.4 Hz), 7.53 (2H, s (broad)), 7.43 (1H, d, J = 2.2 Hz), 6.53 (1H, d, J = 3.2 Hz), 6.52 (1H, t, J = 2.3 Hz), 6.45 (1H, t, J = 2.3 Hz), 6.33 (1H, t, J = 2.4 Hz), 6.18 (1H, d, J = 3.2 Hz), 4.43 (1H, ddd, J = 14.7, 8.2, 2.5 Hz), 4.29 (2H, overlapping), 4.23 (1H, t, J = 5.7 Hz), 3.38 (1H, d, J = 8.2 Hz), 3.14 (3H, s), 2.95 (1H, dt, J = 19.2, 6.2 Hz), 2.87 (1H, dt, J = 19.2, 6.2 Hz), 2.32 (1H, m), 2.09 (1H, m), 1.19 (9H, d, J = 9.3 Hz). **^{13}C NMR (800 MHz, CD_2Cl_2, δ, 25 °C):** 197.1, 162.4, 145.5, 144.9, 143.7, 143.6, 139.0, 138.8, 138.6, 112.8, 108.8, 108.5, 107.9, 107.0, 73.0 (d, J_{CP} = 15.8 Hz), 64.9, 58.3, 37.8, 37.5, 29.0, 28.2, 14.0 (d, J_{CP} = 32.0 Hz). [M]+ calculated for $C_{24}H_{37}BN_8O_3PW^+$ 709.2167; found, 709.2171.

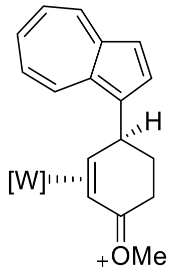

27D

Azulene (209 mg, 1.63 mmol) and MeCN (10 mL) were added to a screw top test-tube charged with a stir pea. To a separate screw top test-tube charged with a stir pea were added **13D** (500 mg, 0.657 mmol) and MeCN (2.50 mL). To another screw top test-tube charged with a stir pea were added HOTf (736 mg, 4.90 mmol) and MeCN (2.50 mL). All three test-tubes were cooled to -30 °C for 5 min. Then, the HOTf/MeCN solution was transferred to the test-tube containing **13D**. The resulting golden solution was allowed to cool for 5 min, and then this solution was transferred dropwise to the test-tube containing the azulene solution. The reaction was allowed to stir at −30 °C for 5 min, after which the reaction solution was diluted with DCM (~5 mL) and washed with DI H_2O (3 x ~3 mL). The reaction mixture was dried with anhydrous Na_2SO_4, which was then filtered off with a 30 mL medium porosity fritted disc. The filtrate was concentrated *in vacuo* to leave a blue-green oil, which was redissolved in minimal DCM and added to stirring Et_2O (~250 mL), forming a light-blue precipitate which was collected on a 30 mL fine porosity fritted disc. The precipitate was washed with Et_2O (1 x ~30 mL) and then desiccated overnight, yielding **27D** (430 mg, 0.484 mmol, 73% yield).

^{1}H NMR (800 MHz, *d3*-MeCN, δ, 25 °C): 8.53 (d, J = 9.7 Hz, 1H), 8.40 (d, J = 9.4 Hz, 1H), 8.21 (d, J = 2.2 Hz, 1H), 8.18 (d, J = 3.9 Hz, 1H), 8.03 (d, J = 2.3 Hz, 1H), 7.96 (d, J = 2.5 Hz, 1H), 7.95 (d, J = 2.6 Hz, 1H), 7.85 (d, J = 2.3 Hz, 1H), 7.69 (t, J = 9.8 Hz, 1H), 7.56 (d, J = 2.2 Hz, 1H), 7.48 (d, J = 3.9 Hz, 1H), 7.25 (t, J = 9.7 Hz, 1H), 7.21 (t, J = 9.6 Hz, 1H), 6.49 (t, J = 2.3 Hz, 1H), 6.48 (t, J = 2.3 Hz, 1H), 6.40 (t, J = 2.3 Hz, 1H), 5.05 (ddd, J = 2.3, 6.2, 8.9 Hz, 1H), 4.38 (ddd, J = 2.4, 8.2, 14.9 Hz, 1H), 3.48 (d, J = 8.1 Hz, 1H), 3.15 (s, 3H), 3.12 (m, 1H), 2.74 (dt, J = 1.6, 18.4 Hz, 1H), 2.36 (m, 1H), 1.88 (m, 1H), 0.98 (d, J = 9.5 Hz, 9H). **^{13}C NMR (800 MHz, CD_2Cl_2, δ, 25 °C):** 197.4, 146.0, 144.9, 143.0, 142.2, 140.0, 139.8, 139.5, 139.2, 138.2, 138.0, 136.9, 135.2, 134.2, 124.1, 123.3, 118.5, 109.1, 108.7, 108.7, 76.9 (d, J = 6.7 Hz), 66.6, 58.7, 37.2, 34.3, 30.1, 13.9 (d, J = 31.5 Hz). **CV (MeCN):** $E_{p,c}$ = -1.22 V (NHE). Anal. Calcd for

$C_{30}H_{36}BF_3N_7O_5PSW$: C, 40.51; H, 4.08; N, 11.02. Found: C, 40.41; H, 4.10; N, 11.15. **ESI-HRMS (m/z):** [M]+ calcd for $C_{30}H_{36}BF_3N_7O_5PSW$ 740.2270; found 740.2269. Crystals suitable for single crystal x-ray diffraction were grown *via* mixed solvent recrystallization (inner chamber = 27D/MeCN, outer chamber = methyl *tert*-butyl ether).

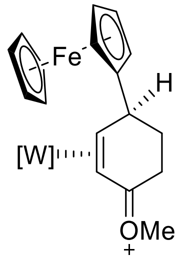

28D

To a 15 mL test tube was added **13D** (0.2036 g, 0.2675 mmol) and MeCN (1 mL). In a separate test tube was added chilled HOTf (0.1627 g, 1.0841 mmol) and MeCN (1 mL). Both solutions were chilled to -30°C for 5 min before the acid solution was added to **2D**. A chilled (-30°C) solution of ferrocene (0.3018 g, 1.6222 mmol) in MeCN was then added. After stirring at -30°C for 18 hrs, the reaction mixture was added to 200 mL of stirring Et_2O to produce a light tan precipitate. The precipitate was collected on a 15 mL fine-porosity fritted disc and washed with diethyl ether (15 mL). This solid was then added to a 4-dram vial charged with a stir bar, Zn powder (~100 mg), and MeCN (2 mL). after stirring for 5 min, the mixture was filtered. The filtrate was then added to 150 mL of stirring Et_2O to produce a light orange precipitate which was collected on a 15 mL fine-porosity fritted disc and washed with diethyl ether (15 mL) to yield **20D** (0.1130g, 44.6%).

^{1}H NMR (800 MHz, CD_2Cl_2, δ, 25 °C): 8.12 (1H, d, *J* = 2.0 Hz), 7.95 (1H, t, *J* = 1.5 Hz), 7.86 (1H, d, *J* = 2.3 Hz), 7.81 (2H, overlapping), 7.42 (1H, d, *J* = 2.0 Hz), 6.53 (1H, dd, *J* = 5.4, 2.4 Hz), 6.46 (1H, t, *J* = 2.3 Hz), 6.33 (1H, t, *J* = 2.3 Hz), 4.40 (under integrates, broad), 4.34 (1H, broad), 4.26 (under integrates,), 4.21 (under integrates, broad), 3.96 (1H, dd, *J* = 13.5, 8.3 Hz), 3.74 (1H, t, *J* = 4.4 Hz), 3.19 (3H, s), 3.16 (1H, d, *J* = 8.5 Hz), 2.90 (2H, overlapping), 2.55 (1H m, 7.2 Hz), 2.27 (1H, m), 1.33 (9H, d, *J* = 9.5 Hz). **^{13}C NMR (800 MHz, CD_2Cl_2, δ, 25 °C):** 196.8, 145.0, 143.5, 142.0, 139.2, 139.1, 138.9, 108.6, 108.6, 108.0, 79.0 (d, J_{CP} = 15.7 Hz), 70.1, 69.8, 69.4, 69.1, 67.0, 66.8, 63.8, 58.4, 37.9, 28.9, 28.1, 14.0 (d, J_{CP} = 31.7 Hz). **APCI-HRMS (m/z):** [M]+ calculated for $C_{29}H_{38}BFeN_7O_2PW^+$ 798.1771; found, 798.1796.

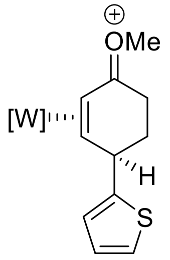

17P

To a 15-mL test tube was added **12** (0.7377 g, 1.207 mmol). The complex was chilled to -60 °C for 15 min before adding a chilled (-60 °C) solution of HOTf in EtCN (2ml, 3.709 mM). After 5 min, a chilled (-60 °C) solution of the thiophene in EtCN (2 mL, 10.02 mM)) was added. The reaction was then allowed to stir at room temperature and was monitored by ^{31}P NMR and determined to be complete based on the disappearance of **14P** and the formation of a new signal with J_{WP} = 300 Hz. The reaction mixture was then diluted with DCM (5 mL) and washed with DI H_2O (3 x 5 mL). The organic layer was dried over anhydrous $MgSO_4$ and concentrated in vacuo. The film was dissolved in minimal DCM and added to stirring Et_2O (300 mL). The resulting precipitate was collected on a fine porosity frit, washed with Et_2O (30 mL), and desiccated to give **17P** (0.7046 g (69.0%)).

^{1}H NMR (600 MHz, *d₆*-acetone, δ, 25 °C): 8.32 (1H, d, J = 2.0 Hz), 8.19 (2H, d, J = 2.2 Hz), 8.17 (1H, d, J = 2.1 Hz), 7.98 (1H, d, J = 2.2 Hz), 7.91 (1H, d, J = 1.9 Hz), 7.28 (1H, dd, J = 5.1, 1.0 Hz), 7.02 (1H, d, J = 3.4 Hz), 6.94 (1H, dd, J = 5.1, 3.5 Hz), 6.58 (1H, t, J = 2.2 Hz), 6.55 (1H, t, J = 2.2 Hz), 6.40 (1H, t, J = 2.2 Hz), 4.97 (1H, t, J = 9.1 Hz), 4.54 (3H, s), 4.07 (1H, b), 3.10 (1H, m), 3.00 (1H, dd, J = 21.1, 7.5 Hz), 2.78 (1H, d, J = 8.4 Hz), 2.62 (1H, m), 2.19 (1H, dd, J = 14.0, 7.7 Hz), 1.24 (9H, d, J_{PH} = 9.4 Hz). **^{31}P NMR (500 MHz, d_6-acetone, δ, 25 °C):** -6.2 (J_{WP} = 297 Hz). **^{13}C NMR (800 MHz, d_6-acetone, δ, 25 °C):** 207.6, 155.0, 144.2, 143.4, 143.2, 139.3, 139.0, 138.3, 127.7, 125.0, 124.4, 108.9, 108.6, 107.8, 75.7, 63.0, 60.2, 36.1, 28.6, 27.4, 11.8 (d, J_{CP} = 30.6). **CV (MeCN):** $E_{1/2}$= - 1.13 V (NHE). **IR (ATR, cm^{-1}):** ν(BH) = 2516 cm^{-1}, ν(NO) = 1600 cm^{-1}. Composition confirmed by single crystal X-ray diffraction.

General Procedure 2: To a 15-mL test tube was added **15D**, **16D**, **17D**, or **17P** and MeOH (5 mL). This solution was chilled to -30 °C for 5 min before adding $NaBH_4$. The reaction mixture was allowed to stir at -30 °C for 30 min before diluting with DCM (5 mL) and washing with DI H_2O (2 x 5 mL). The organic layer was dried over anhydrous Na_2SO_4 and concentrated in vacuo. A solution of HOTf in DME was added to the resulting oil to give a dark red homogenous solution. This solution was then added to stirring Et_2O (300 mL) to produce a precipitate which was collected on a 30 mL medium-porosity fritted funnel, washed with Et_2O (30 mL), and desiccated to give **6D**, **7D**, **8D**, or **8P**.

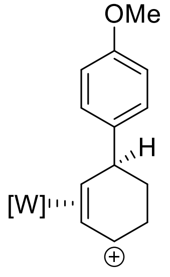

6D

Used General Procedure 2 with **15D** (0.740 g, 0.851 mmol), $NaBH_4$ (0.195 g, 5.15 mmol), and HOTf in DME (3 mL, 0.11 M). Tan solid (0.525 g, (74%)).

^{1}H NMR (800 MHz, d_3-MeCN, δ, 25 °C): 8.41 (1H, d, J = 2.1 Hz), 8.16 (1H, d, J = 2.1 Hz), 8.00 (1H, d, J = 2.3 Hz), 7.95 (1H, d, J = 2.4 Hz), 7.93 (1H, d, J = 2.2 Hz), 7.82 (1H, d, J = 2.4 Hz), 7.35 (2H, d, J = 8.6 Hz), 6.97 (2H, d, J = 8.7 Hz), 6.54 (1H, t, J = 6.7 Hz), 6.53 (1H, t, J = 2.2 Hz), 6.50 (1H, t, J = 2.3 Hz), 6.35 (1H, t, J = 2.3 Hz), 5.39 (1H, t, J = 7.4 Hz), 4.36 (1H, dd, J =

15.8, 7.1 Hz), 3.80 (3H, s), 3.73 (1H, dd, J = 10.8, 6.1 Hz), 3.50 (1H, dt, J = 18.0, 7.9 Hz), 3.31 (1H, dt, J = 19.5, 6.2 Hz), 1.77 (1H, m), 1.22 (1H, m), 1.01 (9H, d, J_{PH} = 9.8 Hz). **^{13}C NMR (800 MHz, d_3-MeCN, δ, 25 °C):** 159.5, 149.2, 146.1, 143.3, 141.5, 139.6, 139.5, 139.5, 134.4, 129.4, 115.1, 109.5, 108.9, 108.1, 105.4 (d, J_{PC} = 3.3 Hz) 75.4 (d, J_{PC} = 12.2), 55.9, 44.5 (d, J_{PC} = 2.4 Hz), 32.1, 26.2, 13.8 (d, J_{CP} = 32.7 Hz). **CV (DMA):** $E_{p,c}$ = - 0.93 V (NHE). **IR (ATR, cm^{-1}):** ν(BH) = 2519 cm^{-1}, ν(NO) = 1635 cm^{-1}.

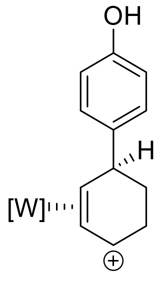

7D

Used General Procedure 2 with **16D** (0.5049 g, 0.5903 mmol), $NaBH_4$ (0.1793 g, 4.740 mmol), and HOTf in DME (2 mL, 0.6960 mM). Tan solid (0.3739 g, (76.7%)).

^{1}H NMR (800 MHz, d_3-MeCN, δ, 25 °C): 8.44 (1H, d, J = 2.1 Hz), 8.18 (1H, d, J = 2.1 Hz), 8.03 (1H, d, J = 2.3 Hz), 7.98 (1H, d, J = 2.3 Hz), 7.95 (1H, d, J = 2.1 Hz), 7.85 (1H, d, J = 2.4 Hz), 7.28 (2H, d, J = 8.5 Hz), 6.88 (2H, d, J = 8.5 Hz), 6.55 (2H, overlapping), 6.53 (1H, t, J = 2.3 Hz), 6.38(1H, t, J = 2.3 Hz), 5.40 (1H, t, J = 7.4 Hz,), 4.38 (1H, dd, J = 15.9, 7.1 Hz), 3.72 (1H, dd, J = 10.8, 6.1 Hz), 3.52 (1H, m), 3.33 (1H, dt, J = 19.5, 6.2 Hz), 1.79 (1H, m), 1.24 (1H, m), 1.03 (9H, d, J_{PH} = 9.8 Hz). **^{13}C NMR (800 MHz, d_3-MeCN, δ, 25 °C):** 156.7, 149.2, 146.2, 143.3, 140.7, 139.5, 139.4, 134.3, 129.5, 116.5, 109.5, 108.9, 108.1, 105.5 (J_{PC} = 3.4 Hz), 75.6 (d, J_{PC} = 12.0 Hz), 44.5 (d, J_{PC} = 2.2 Hz), 32.2, 30.8, 26.2, 13.7 (d, J_{CP} = 32.7 Hz). **CV (MeCN):** $E_{p,c}$= - 0.92 V (NHE). **IR (ATR, cm^{-1}):** ν(BH) = 2523 cm^{-1}, ν(NO) = 1635 cm^{-1}.

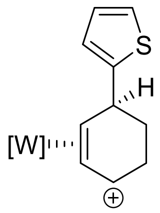

8D

Used General Procedure 2 with **17D** (0.4914 g, 0.5813 mmol), $NaBH_4$ (0.1625 g, 4.296 mmol), and HOTf in DME (2 mL, 0.9780 mM). Tan solid (0.3803 g, (80.2%)).

^{1}H NMR (600 MHz, d_3-MeCN, δ, 25 °C): 8.42 (1H, d, J = 2.1 Hz), 8.14 (1H, d, J = 2.2 Hz), 8.02 (1H, d, J = 2.3 Hz), 7.97 (1H, m), 7.95 (1H, d, J = 2.2 Hz), 7.83 (1H, m), 7.36 (1H, m), 7.11 (1H, m), 7.05 (1H, dd, J = 5.1, 3.5 Hz), 6.66 (1H, t, J = 7.1 Hz), 6.54 (1H, t, J = 2.4 Hz), 6.52 (1H, t, J = 2.3 Hz), 6.36 (1H, t, J = 2.4 Hz), 5.31 (1H, t, J = 7.5 Hz), 4.36 (1H, dd, J = 15.3, 7.2 Hz), 4.11 (1H, m), 3.46 (1H, burried), 3.32 (1H, m), 1.94 (1H, burried), 1.46 (1H, m), 1.10 (9H, d, J_{PH} = 9.9). **^{13}C NMR (800 MHz, d_3-MeCN, δ, 25 °C):** 153.6, 149.4, 146.4, 143.7, 139.7, 139.6, 139.4, 136.7, 127.9, 125.1, 124.9, 109.6, 108.2, 103.7 (d, J_{PC} = 2.8 Hz), 74.5 (d, J_{PC} = 12.8 Hz), 40.0 (d,

J_{PC} = 2.5 Hz), 32.0, 25.9, 13.6 (d, J_{CP} = 32.8 Hz). **CV (DMA):** $E_{p,c}$ = - 1.05 V (NHE). **IR (ATR, cm^{-1}):** ν(BH) = 2530 cm^{-1}, ν(NO) = 1633 cm^{-1}.

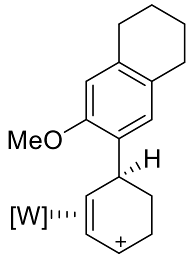

41D

Used General Procedure 2 with **24D** (1.8298 g, 1.9816 mmol), $NaBH_4$ (0.7722 g, 20.41 mmol), and HOTf (0.9970 g, 6.6431 mmol) in DME (2 mL). Tan solid (1.3477 g, (76.13%)).

^{1}H NMR (800 MHz, *d_3*-MeCN, δ, 25 °C): 8.42 (d1H, *J* = 2.2 Hz), 8.17 (1H, d, *J* = 2.2 Hz), 8.00 (1H, d, *J* = 2.4 Hz), 7.95 (1H, d, *J* = 2.4 Hz), 7.93 (1H, d, *J* = 2.3 Hz), 7.82 (1H, d, *J* = 2.5 Hz), 7.17 (1H, s), 6.68 (1H, s), 6.52 (3H, overlapping), 6.35 (1H, t, *J* = 2.4 Hz), 5.35 (1H, t, *J* = 7.4 Hz), 4.30 (2H, overlapping), 3.78 (3H, s), 3.50 (1H, m), 3.32 (1H, m), 2.77 (4H, overlapping), 1.80 (4H, overlapping), 1.68 (1H, m), 1.18 (1H, buried), 1.02 (9H, d, *J* = 9.9 Hz). **^{13}C NMR (800 MHz, *d_3*-MeCN, δ, 25 °C):** 154.7, 149.2, 146.1, 143.3, 139.6, 139.5, 139.4, 137.4, 134.5, 133.6, 130.6, 129.5, 111.9, 109.5, 108.9, 108.1, 105.4 (d, J_{CP} = 3.9 Hz), 76.4 (d, J_{CP} = 12.5 Hz), 56.1, 36.1, 30.5, 30.0, 29.2, 26.2, 24.2, 24.0, 13.5 (d, J_{CP} = 32.6 Hz). Composition confirmed by single crystal X-ray diffraction.

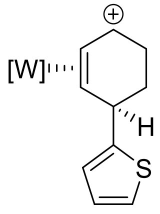

8P

Used General Procedure 2 with **17P** (0.4165 g, 0.4828 mmol), $NaBH_4$ (0.1141 g, 3.02 mmol), and HOTf in DME (2 mL, 0.3292 mM). Tan-pink solid (0.1812 g, (45.1%)).

^{1}H NMR (600 MHz, *d_3*-MeCN, δ, 25 °C): 8.42 (1H, d, *J* = 1.8 Hz), 8.01 (1H, d, *J* = 2.2 Hz), 7.98 (1H, d, *J* = 2.0 Hz), 7.96 (1H, d, *J* = 2.0 Hz), 7.94 (1H, d, *J* = 2.1 Hz), 7.80 (1H, d, *J* = 2.2 Hz), 7.36 (1H, dd, = 5.1, 1.0 Hz), 7.15 (1H, d, *J* = 3.3 Hz), 7.07 (1H, dd, *J* = 5.1, 3.5 Hz), 6.55 (1H, t, *J* = 2.3 Hz), 6.53 (1H, t, *J* = 2.3 Hz), 6.29 (1H, t, *J* = 2.3 Hz), 6.04 (1H, d, *J* = 6.5 Hz), 5.31 (1H, t, *J* = 7.3 Hz), 4.80 (1H, m), 4.73 (1H, dt, *J* = 15.1, 7.2 Hz), 3.27 (1H, m), 2.56 (1H, m), 1.99 (1H, buried), 1.46 (1H, m), 1.22 (9H, d, J_{PH} = 9.9 Hz).

^{13}C NMR (800 MHz, *d_3*-MeCN, δ, 25 °C): 150.2, 148.5, 146.2, 143.4, 139.4, 139.3, 128.4, 128.0, 125.3, 125.2, 109.3, 109,0 108.0, 104.9, 73.5 (d, J_{CP} = 11.3 Hz), 38.0, 30.4, 25.5, 12.8 (d, J_{CP} = 32.9 Hz). **CV (MeCN):** $E_{p,c}$= - 0.95 V (NHE). **IR (ATR, cm^{-1}):** ν(BH) = 2515 cm^{-1}, ν(NO) = 1634 cm^{-1}.

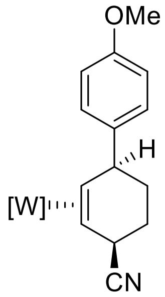

9D

A solution of **6d** (0.405 g, 0.483 mmol) was prepared in MeCN (12.15 mL) in a large test-tube with a stir-pea. A NaCN/MeOH solution (20 mg/mL, 2.37 mL, 0.966 mmol) was added to the solution and the reaction mixture was allowed to stir at room temperature for 22 hrs. At this time, a white solid was observed to have precipitated out of solution, which was then collected on a 30 mL F frit and desiccated to give **9P** (0.160 g (46%)).

^{1}H NMR (600 MHz, d_3-MeCN, δ, 25 °C): 8.07 (1H, d, J = 2.1 Hz), 8.05 (1H, d, J = 2.1 Hz), 7.88 (1H, d, J = 2.4 Hz), 7.85 (1H, d, J = 2.3 Hz), 7.84 (1H, d, J = 2.5 Hz), 7.54 (2H, d, J = 8.7 Hz), 7.31 (1H, d, J = 2.2 Hz), 6.97 (2H, d, J = 8.7 Hz), 6.39 (1H, t, J = 2.2 Hz), 6.35 (1H, t, J = 2.2 Hz), 6.25 (1H, t, J = 2.2 Hz), 4.11 (1H, t, J = 6.3 Hz), 4.04 (1H, m), 3.81 (3H, s), 2.73 (1H, t, J = 12.2 Hz), 2.00 (m, 1H), 1.93 (buried, m, 1H), 1.71 (1H, m), 1.51 (1H, m), 1.37 (1H, dt, J = 11.3, 2.4 Hz), 0.97 (9H, d, J_{PH} = 8.4 Hz). **^{13}C NMR (800 MHz, d_3-MeCN, δ, 25 °C):** 158.9, 145.5, 144.4, 143.1, 141.8, 138.1, 137.9, 137.6, 130.2, 128.4, 114.7, 107.8, 107.3, 107.1, 55.9, 55.4 (d, J_{CP} = 11.0 Hz), 53.4, 44.9 (d, J_{CP} = 3.3 Hz), 32.8, 31.6, 24.7, 13.8 (d, J_{CP} = 28.3 Hz). **CV (MeCN):** $E_{p,a}$ = 0.59 V (NHE). Composition confirmed by single crystal X-ray diffraction.

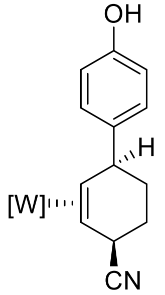

10D

To a 4-dram vial was added **7D** (0.3243 g, 0.3929 mmol), NaCN (0.0963 g, 1.965 mmol), DMSO (1 mL), and MeCN (1 mL). After stirring for 18 hrs, the heterogenous reaction mixture was passed through a 30 mL M frit to collect an off-white precipitate. The precipitate was washed with DI H_2O (3 x 10 mL), Et_2O (3 x 10 mL), pentanes (3 x 10 mL), then desiccated overnight to give **10D** (0.1422 g (51.5%)).

^{1}H NMR (600 MHz, d_6-acetone, δ, 25 °C): 8.12 (1H, d, J = 1.8 Hz), 8.10 (1H, d, J = 1.9 Hz), 7.95 (1H, d, J = 2.3 Hz), 7.94 (1H, d, J = 2.2 Hz), 7.87 (1H, d, J = 2.4 Hz), 7.43 (3H, overlapping), 6.88 (1H, d, J = 8.5 Hz), 6.41 (1H, t, J = 2.2 Hz), 6.35 (1H, t, J = 2.2 Hz), 6.30 (1H, t, J = 2.2 Hz), 4.11 (1H, t, J = 6.4 Hz), 4.06 (1H, m), 2.82 (1H, m), 2.02 (1H, m), 1.96 (1H, m), 1.75 (1H, m), 1.53 (1H, m), 1.42 (1H, d, J = 11.3 Hz), 1.03 (9H, d, J = 8.4 Hz). **^{13}C NMR (800 MHz, d_6-acetone,**

δ, 25 °C): 156.3, 144.4, 144.3, 143.2, 141.6, 137.8, 137.5, 137.3, 130.0, 128.2, 116.1, 107.5, 107.3, 106.9, 55.1 (d, J_{CP} = 10.8 Hz), 53.7, 44.9, 33.3, 31.5, 25.0, 14.0 (d, J_{CP} = 28.2 Hz). **CV (THF):** $E_{p,a}$= 0.63 V (NHE). **IR (ATR, cm^{-1}):** ν(BH) = 2503 cm^{-1}, ν(NO) = 1513 cm^{-1}, ν(CN) = 2222 cm^{-1}. Composition confirmed by single crystal X-ray diffraction.

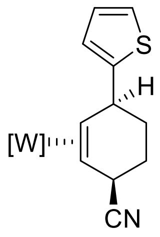

11D

To a 4-dram vial was added **8D** (0.7111 g, 0.8722 mmol), NaCN (0.1542 g, 3.146 mmol), DMSO (1 mL), and MeCN (1 mL). After stirring for 18 hrs, the reaction mix was diluted with DCM (5 mL) and washed with DI H_2O (3 x 15 mL). The organic layer was dried over anhydrous $MgSO_4$ and diluted with pentanes (200 mL). Evaporated down to ~100 mL before diluting with pentanes (50 mL). A white solid was collected on a 30 mL F frit, washed with pentanes (30 mL), and desiccated overnight to give **11D** (0.4216 g (69.8%)).

^{1}H NMR (800 MHz, *d$_6$*-acetone, δ, 25 °C): 8.14 (1H, d, *J* = 1.8 Hz), 8.13 (1H, d, *J* = 1.9 Hz), 7.97 (1H, d, *J* = 2.3 Hz), 7.96 (1H, d, *J* = 2.2 Hz), 7.88 (1H, d, *J* = 2.4 Hz), 7.45 (1H, d, *J* = 2.1 Hz) 7.32 (1H, dd, *J* = 5.1, 1.1 Hz), 7.19 (1H, dt, *J* = 3.4, 0.9 Hz), 7.04 (1H, dd, *J* = 5.1, 3.4 Hz), 6.43 (1H, t, *J* = 2.0 Hz), 6.35 (1H, t, *J* = 2.1 Hz), 6.33 (1H, t, *J* = 2.1 Hz), 4.47 (1H, t, *J* = 6.1), 4.13 (1H, m), 2.87 (1H, m), 2.11 (1H, m), 2.02 (1H, m), 1.83 (1H, m), 1.71 (1H, m), 1.39 (1H, dt, *J* = 11.2, 2.1 Hz), 1.08 (9H, d, J_{PH} = 8.4). **^{13}C NMR (800 MHz, *d$_6$*-acetone, δ, 25 °C):** 158.1, 144.4, 143.0, 141.6, 138.0, 137.5, 137.4, 127.8, 127.6, 124.6, 123.8, 107.6, 107.3, 106.9, 55.5 (d, J_{CP} = 12.8 Hz), 52.6, 41.5, 33.6, 31.4, 25.3, 13.8 (d, J_{CP} = 28.2 Hz). **CV (MeCN):** $E_{p,a}$= 0.70 V (NHE). **IR (ATR, cm^{-1}):** ν(BH) = 2492 cm^{-1}, ν(NO) = 1549 cm^{-1}, ν(CN) = 2228 cm^{-1}. Composition confirmed by single crystal X-ray diffraction.

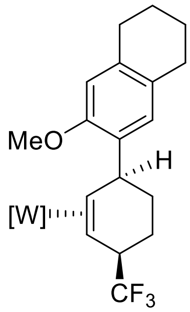

29D

To a 30 mL test tube was added **41D** (0.4228 g, 0.4733 mmol), THF (15 mL), and CF_3TMS (0.9626 g, 6.770 mmol). This solution was chilled to -60 °C for15 min before slowly adding a chilled (-60 °C) solution of Bu_4NF (0.55 mL of 1.0 M in THF) in 5 mL of THF over the course of 15 min. After 18 hrs, the reaction mix was diluted with DCM (~5 mL), washed with 20 mL of DI H_2O (2

x 10 mL) and then washed with 10 mL of brine. The organic layer was dried over $MgSO_4$ and evaporated to an oil. The oil was dissolved in minimal DCM and added to 250 mL of stirring hexanes. This heterogeneous mixture was evaporated down to 50 mL before collecting a tan-white solid on 30 mL F frit and washing with 30 mL of pentane (2 x 15 mL) to yield **29D** (0.2113 g (54.90%)).

^{1}H NMR (600 MHz, CD_2Cl_2, δ, 25 °C): 8.11 (1H, s), 8.06 (1H, d, J = 2.1 Hz), 7.76 (1H, d, J = 2.1 Hz), 7.75 (1H, d, J = 2.0 Hz), 7.73 (1H d, J = 2.1 Hz), 7.56 (1H, s), 7.30 (1H, m), 6.62 (1H, s), 6.34 (1H, t, J = 2.0 Hz), 6.26 (1H, t, J = 2.1 Hz), 6.20 (1H, t, J = 2.0 Hz), 4.63 (1H, m), 3.86 (4H, overlapping), 2.79 (4H, overlapping), 2.64 (1H, t, J = 12.2 Hz), 2.19 (1H, m), 1.82 (4H, overlapping), 1.63 (2H, overlapping), 1.39 (2H, overlapping), 1.04 (9H, d, J = 8.2 Hz). **^{13}C NMR (800 MHz, CD_2Cl_2, δ, 25 °C):** 154.7, 143.3, 142.3, 140.8, 138.2, 137.2, 136.9, 136.4, 135.3, 131.6 (q, J_{CF} = 280.2 Hz), 131.0, 128.9, 110.9, 106.9, 106.4, 105.7, 58.4 (d, J_{CP} = 10.4 Hz), 56.0, 47.3, 43.3(q, J_{CF} = 23.9 Hz), 36.7, 30.0, 29.4, 26.2, 24.3, 24.1, 18.8, 13.5 (d, J_{CP} = 28.6 Hz). Composition confirmed by single crystal X-ray diffraction.

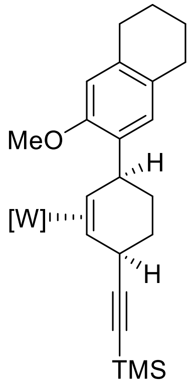

30D

To a 30 mL test tube was added **41D** (0.3266 g, 0.3656 mmol) and THF (10 mL). This solution was chilled to -60 °C. To a separate 15 mL test tube was added trimethylsilylacetylene (0.6900 g, 7.025 mmol) and THF (5 mL). A solution of nBuLi (0.5 mL, 1.6 M in hexanes) was then added dropwise to the test tube containing trimethylsilylacetylene. This solution was allowed to stir at RT for 5 min before chilling to -60 °C for 5 min and adding to the test tube containing **41D.** After 18 hrs at -60 °C, reaction mix diluted with DCM (~5 mL) and washed with 20 mL of sat. Na_2CO_3 (2 x 10 mL). The organic layer was dried over Na_2SO_4 and evaporated to an oil. The film dissolved in minimal DCM and added to 150 mL of stirring pentanes. A tan-white solid collected on 15 mL F frit and washed with 30 mL of pentane (2 x 15 mL) to yield **30D** (0.1698 g (55.19%)).

^{1}H NMR (800 MHz, d_6-acetone, δ, 25 °C): 8.41 (1H, d, J = 2.0 Hz), 8.14 (1H, d, J = 2.0 Hz), 7.94 (1H, d, J = 2.3 Hz), 7.92 (1H, d, J = 2.3 Hz), 7.83 (1H, d, J = 2.4 Hz), 7.62 (1H, s), 7.39 (1H, d, J = 2.1 Hz), 6.65 (1H, s), 6.42 (1H, t, J = 2.2 Hz), 6.30 (1H, t, J = 2.2 Hz), 6.26 (1H, t, J = 2.2 Hz), 4.70 (1H, dd, J = 6.3, 4.4 Hz), 4.12 (1H, dt, J = 8.7, 3.9 Hz), 3.83 (3H, s), 2.76 (4H, overlapping), 2.61 (1H, t, J = 11.6 Hz), 2.07 (1H, m), 1.78 (6H, overlapping), 1.60 (1H, m,), 1.48 (1H, m), 1.06 (9H, d, J = 8.2 Hz), 0.08 (9H, s). **^{13}C NMR (800 MHz, d_3-acetone, δ, 25 °C):** 155.2, 144.1, 144.1, 143.9, 141.7, 139.8, 137.8, 136.9, 136.9, 135.2, 131.1, 128.9, 118.6, 111.4, 107.4, 107.0, 106.2, 83.4, 66.3, 58.3 (d, J_{CP} = 10.4 Hz), 57.4, 56.0, 36.9, 36.9, 34.2, 27.9, 24.6, 24.4, 13.6

(d, J_{CP} = 27.6 Hz), 0.7. **APCI-HRMS (m/z):** [M+H] calculated for $C_{34}H_{49}BN_7O_2PSiW$ 842.3130; found, 842.3155.

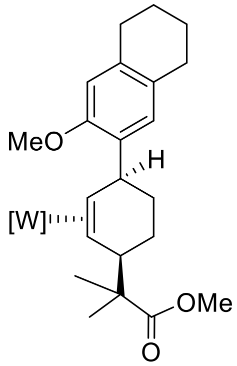

31D

To a 4-dram vial was added **41D** (0.3696 g, 0.4137 mmol), MeCN (~5 mL), and methyl trimethylsilyl dimethylketene acetal (0.8580 g, 4.922 mmol). After 18 hrs, the reaction mix was diluted with DCM (~5 mL) and washed with 20 mL of DI H_2O (2 x 10 mL). The organic layer was dried over $MgSO_4$ and evaporated to an oil. The oil was dissolved in minimal DCM and added to 300 mL of stirring pentanes. A tan-white solid collected on 30 mL F frit and washed with 30 mL of pentane (2 x 15 mL) to yield **31D** (0.2733 g (78.13%)).

^{1}H NMR (800 MHz, CD_2Cl_2, δ, 25 °C): 8.14 (1H, d, J = 1.9 Hz), 7.98 (1H, d, J = 1.9 Hz), 7.76 (1H, d, J = 2.2 Hz), 7.74 (1H, d, J = 2.5 Hz), 7.71 (1H, d, J = 2.3 Hz), 7.54 (1H, s), 7.29 (1H, d, J = 2.2 Hz), 6.59 (1H, s), 6.29 (2H, overlapping), 6.24 (1H, t, J = 2.3 Hz), 4.54 (1H, dd, J = 6.0, 2.4 Hz), 3.82 (3H, s), 3.46 (3H, s), 3.33 (1H, dd, J = 11.2, 6.1 Hz), 2.89 (1H, dd, J = 15.5, 11.6 Hz), 2.78 (4H, overlapping), 2.20 (1H, m), 1.82 (4H, overlapping), 1.30 to 1.09 (4H, overlapping), 1.07 (9H, d, J = 7.9 Hz), 1.04 (3H, s), 0.67 (3H, s). **^{13}C NMR (800 MHz, CD_2Cl_2, δ, 25 °C):** 179.3, 154.9, 143.5, 143.0, 140.3, 138.5, 137.3, 136.7, 136.3, 135.0, 131.2, 128.4, 110.8, 106.6, 106.5, 106.3, 58.6 (d, J_{CP} = 9.8 Hz), 56.0, 54.4, 51.6, 49.7, 43.8, 37.4, 30.0, 29.4, 27.0, 24.3, 24.1, 24.0, 21.1, 20.5, 13.8 (d, J_{CP} = 26.8 Hz). Composition confirmed by single crystal X-ray diffraction.

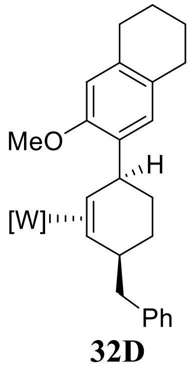

32D

To a 30 mL test tube was added **41D** (0.4753 g, 0.5320 mmol) and THF (8 mL). This solution was chilled to -60 °C for 20 min before slowly adding a chilled (-60 °C) solution of BnMgCl (2.0 mL, 1.0 M) in THF (5 mL). After 18 hrs at -60 °C, the reaction mix was diluted with DCM (~5 mL)

and washed with 20 mL of DI H_2O (2 x 10 mL). The organic layer was dried over $MgSO_4$ and evaporated to an oil. The oil was dissolved in minimal DCM and added to 300 mL of stirring pentanes. A tan-white solid collected on 30 mL F frit and washed with 30 mL of pentane (2 x 15 mL) to yield **32D** (0.1946 g (43.78%)).

^{1}H NMR (800 MHz, CD_2Cl_2, δ, 25 °C): 8.48 (1H, d, J = 2.0 Hz), 8.06 (1H, d, J = 2.0 Hz), 7.76 (2H, d, J = 2.4 Hz), 7.75 (1H, d, J = 2.3 Hz,), 7.51 (1H, s), 7.30 (1H, d, J = 2.2 Hz), 7.22 (4H overlapping), 7.13 (1H, m), 6.59 (1H, s), 6.36 (1H, t, J = 2.2 Hz), 6.34 (1H, t, J = 2.2 Hz), 6.19 (1H t, J = 2.2 Hz), 4.62 (1H, dd, J = 6.4, 2.4 Hz), 3.81 (3H, s), 3.35 (1H, m), 2.81 (5H, overlapping), 2.67 (2H, t, J = 12.2 Hz), 2.44 (1H, dd, J = 13.4, 11.0 Hz), 2.02 (1H, td, J = 12.3, 11.8, 6.5 Hz), 1.83 (4H, overlapping), 1.39 (1H, dd, J = 11.5, 2.3 Hz), 1.27 (3H, overlapping), 1.04 (9H, d, J = 8.0 Hz). **^{13}C NMR (800 MHz, CD_2Cl_2, δ, 25 °C):** 154.8, 143.3, 142.6, 142.2, 140.8, 139.3, 136.9, 136.7, 136.2, 134.9, 130.9, 129.8, 128.7, 128.4, 125.9, 110.9, 106.8, 106.2, 105.8, 60.0 (d, J_{CP} = 9.9 Hz), 59.1, 56.0, 48.9, 41.5, 37.6, 30.0, 29.6, 28.5, 25.5, 24.3, 24.1, 13.7 (d, J_{CP} = 27.1 Hz). **APCI-HRMS (m/z):** [M+H] calculated for $C_{36}H_{47}BN_7O_2PW$ 836.3204; found, 836.3201.

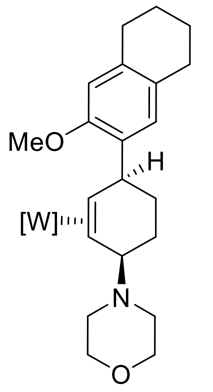

33D

To a 15 mL test tube was added **41D** (0.4511 g, 0.5049 mmol) and THF (5 mL). To a separate 15 mL test tube was added morpholine (0.2965 g, 3.403 mmol) and THF (2 mL). Both solutions were allowed to chill to -45 °C for 5 min before adding the amine solution to the test tube containing **41D**. After 18 hrs at -45 °C, a RT solution of K(tBuO) in THF (1.1380 g, 20% w/w) was added. The reaction mix was diluted with DCM (~5 mL) and washed with 20 mL of sat. Na_2CO_3 (2 x 10 mL). The organic layer was dried over Na_2SO_4 and evaporated to an oil. The film was dissolved in minimal DCM and added to 250 mL of stirring pentanes. A tan-white solid collected on 30 mL F frit and washed with 30 mL of pentane (2 x 15 mL) to yield **33D** (0.2017 g (48.10%)).

^{1}H NMR (800 MHz, d_3-MeCN, δ, 25 °C): 8.45 (1H, d, J = 2.0 Hz), 8.11 (1H, d, J = 2.0 Hz), 7.88 (1H, d, J = 2.4 Hz), 7.82 (1H, d, J = 2.3 Hz), 7.75 (1H, d, J = 2.5 Hz), 7.57 (1H, s), 7.35 (1H, d, J = 2.2 Hz), 6.66 (1H, s), 6.40 (1H, t, J = 2.2 Hz), 6.26 (1H, t, J = 2.3 Hz), 6.20 (1H, t, J = 2.3 Hz), 4.55 (1H, d, J = 5.9 Hz), 4.37 (1H, m), 3.80 (3H, s), 3.59 (2H, overlapping), 3.49 (2H, overlapping), 2.89 (2H, overlapping), 2.76 (4H, overlapping), 2.58 (overlapping, 3H), 2.12 (m, 1H), 1.80 (overlapping, 4H), 1.45 (dt, J = 11.5, 2.4 Hz, 1H), 1.34 (3H, overlapping), 0.98 (9H, d, J = 8.2 Hz). **^{13}C NMR (800 MHz, d_3-MeCN, δ, 25 °C):** 154.3, 143.6, 142.9, 141.0, 138.2, 137.0, 136.1, 135.9, 134.5, 130.6, 127.8, 110.6, 106.6, 105.9, 105.0, 67.5, 66.6, 65.3, 59.3 (d, J_{CP} = 10.3 Hz), 55.4, 55.3, 37.0, 33.9, 29.0, 28.6, 26.1, 23.5, 23.2, 22.1, 16.7, 12.0 (d, J_{CP} = 27.9 Hz). **APCI-**

HRMS (m/z): [M+H] calculated for $C_{36}H_{46}BN_8O_3PW$ 831.3262; found, 744.2603 corresponding to [M-morpholine]+, $C_{29}H_{40}BN_7O_2PW^+$.

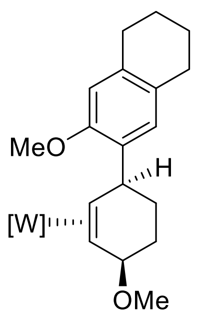

34D

To a 15 mL test tube was added **41D** (0.2090 g, 0.2342 mmol) and MeOH (3 mL). This solution was chilled to -60 °C for 5 min before adding a chilled (-60 °C) solution of KOH in MeOH (1.0 mL, 2 M). After 18 hrs at -60 °C, reaction mix diluted with DCM (~5 mL) and washed with 20 mL of sat. Na_2CO_3 (2 x 10 mL). The organic layer was dried over Na_2SO_4 and evaporated to an oil. The film was dissolved in minimal DCM and added to 250 mL of stirring pentanes. A tan-white solid collected on 30 mL F frit and washed with 30 mL of pentane (2 x 15 mL). to give **34D** (0.1200 g (49.8%)).

^{1}H NMR (800 MHz, *d$_3$*-MeCN, δ, 25 °C): 8.13 (1H, d, *J* = 2.0 Hz), 8.07 (1H, d, *J* = 2.0 Hz), 7.86 (1H, d, *J* = 2.4 Hz), 7.81 (1H, d, *J* = 2.3 Hz), 7.78 (1H, d, *J* = 2.4 Hz), 7.48 (1H, s), 7.33 (1H, d, *J* = 2.1 Hz), 6.65 (1H, s), 6.38 (1H, t, *J* = 2.2 Hz), 6.28 (1H, t, *J* = 2.3 Hz), 6.21 (1H, t, *J* = 2.2 Hz), 4.55 (1H, dd, *J* = 7.3, 4.2 Hz), 4.51 (1H, t, *J* = 6.0 Hz), 3.79 (3H, s), 3.26 (3H, s), 2.77 (4H, overlapping), 2.66 (1H, t, *J* = 12.2 Hz), 1.93 (1H, buried), 1.80 (5H, overlapping), 1.40 (2H, overlapping), 1.32 (1H, dd, *J* = 11.5, 2.0 Hz), 0.95 (9H, d, *J* = 8.4 Hz). **^{13}C NMR (800 MHz, *d$_3$*-MeCN, δ, 25 °C):** 155.1, 144.1, 143.4, 141.8, 139.3, 137.8, 137.3, 137.1, 135.4, 130.7, 129.4, 111.5, 107.5, 106.9, 106.5, 83.1, 57.9 (d, J_{CP} = 9.9 Hz), 57.8, 56.0, 55.1, 37.1, 30.0, 29.5, 28.2, 25.6, 24.4, 24.2, 13.6 (d, J_{CP} = 28.9 Hz).

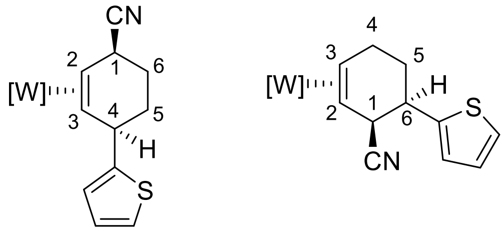

11P(a) **11P(b)**

To a 4-dram vial was added **8P** (0.0986 g, 0.1209 mmol), NaCN in methanol solution (2.2 M, 0.5 mL), and MeCN (1 mL). After stirring for 18 hrs, the reaction mix was diluted with DCM (5 mL) and washed with DI H_2O (3 x 5 mL). The organic layer was dried over anhydrous $MgSO_4$ and diluted with pentanes (100 mL). Evaporated down to ~50 mL before diluting with pentanes (25 mL). A white solid was collected on a 15 mL F frit, washed with pentanes (15 mL), and desiccated overnight to give a 2:1 mixture of **11P(a)** and **11P(b)** (0.0447 g (53.4%)). Full characterization of

11P was made difficult due to overlapping peaks of the two products. However, an NOE interaction between H1 (4.11 ppm) and H6 (3.65 ppm) for **11P(b)** suggests the minor product has a 3,4-substituion pattern. In product **11P(a)**, H1 (3.86 ppm) and H4 (4.59 ppm) have NOE interactions with the PMe_3 ligand (1.20 ppm) and a pyrazole ring proton (8.13 ppm), respectively, but not with each other. Key ^{1}H NMR peaks of the cyclohexene ligand are given.

^{1}H NMR (800 MHz, CD_2Cl_2, δ, 25 °C): 11P(a): 8.13 (1H, d, J = 2.1 Hz, Pz 3/5), 4.59 (1H, m, H4), 3.86 (1H, t, J = 5.4 Hz, H1), 2.82 (overlapping, t, J = 11.5 Hz, H2), 1.49 (1H, m, H3), 1.20 (9H, d, J = 8.9 Hz, PMe_3). **^{1}H NMR (800 MHz, CD_2Cl_2, δ, 25 °C): 11P(b):** 7.93 (0.5H, d, J = 2.0 Hz, Pz 3/5), 4.11 (0.5H, m, H1), 3.65 (0.5H, dt, J = 9.4, 4.4 Hz, H6), 2.82 (overlapping, m, H3), 1.41 (0.5H, m, H2), 1.22 (4.5H, d, J = 8.3 Hz, PMe_3).

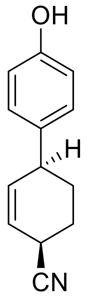

21

To a 4-dram vial was added **10D** (0.1466 g, 0.2088 mmol), $NOPF_6$ (0.0613 g, 0.3503 mmol), and acetone (1 mL). After stirring for 30 min, the reaction mix was evaporated to a film, dissolved in minimal CH_2Cl_2, and added to a 1:1 solution of hexanes and Et_2O (200 mL). A dark-brown precipitate was collected on a 30 mL F frit and washed with Et_2O (100 mL). The filtrate was evaporated to dryness, dissolved in minimal CH_2Cl_2, and purified via silica gel flash chromatography (EtOAc:hexanes, ~40%). Product isolated as an off-white solid and desiccated overnight to give **21** (0.0290 g (69.7%)).

^{1}H NMR (800 MHz, *d_6*-acetone, δ, 25 °C): 8.19 (1H, s), 7.05 (2H, d, J = 8.5 Hz), 6.80 (2H, d, J = 8.5 Hz), 5.93 (1H, dt, J = 9.9, 2.4 Hz), 5.86 (1H, ddd, J = 9.9, 4.2, 2.4 Hz), 3.49 (1H, m), 3.39 (1H, m), 2.03 (1H, buried), 1.97 (2H, overlapping), 1.71 (1H, m). **^{13}C NMR (800 MHz, *d_6*-acetone, δ, 25 °C):** 157.0, 136.3, 135.9, 129.5, 123.0, 122.0, 116.3, 41.2, 30.5, 26.8, 25.8. **IR (ATR, cm^{-1}):** ν(CN) = 2249 cm^{-1}. Composition confirmed by single crystal X-ray diffraction.

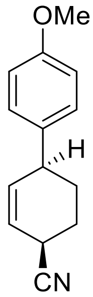

22

To a 4-dram vial was added **9D** (0.100 g, 0.140 mmol), $NOPF_6$ (0.035 g, 0.200 mmol), and acetone (1 mL). After stirring for 30 min, the reaction mix was evaporated to a film, dissolved in minimal CH_2Cl_2, and added to a 1:1 solution of hexanes and Et_2O (200 mL). A dark-brown precipitate was collected on a 30 mL F frit and washed with Et_2O (100 mL). The filtrate was evaporated to dryness, dissolved in minimal CH_2Cl_2, and purified via silica gel flash chromatography (EtOAc:hexanes, ~40%). Product isolated as tan oil and desiccated overnight to give **20** (0.010 g (34%)).

^{1}H NMR (600 MHz, *d₃*-MeCN, δ, 25 °C): 7.13 (2H, d, *J* = 8.6 Hz), 6.88 (2H, d, *J* = 8.4 Hz), 5.93 (1H, m), 5.84 (1H, m), 3.76 (3H, s), 3.41 (2H, m), 2.02 (1H, m), 1.94 (2H, buried, m), 1.67 (1H, m). **^{13}C NMR (800 MHz, d_3-MeCN, δ, 25 °C):** 159.4, 137.7, 135.9, 129.6, 123.0, 122.6, 115.0, 55.9, 41.0, 30.4, 26.9, 25.8. **GCMS (EI)** calculated: 213.28, observed 213.05. **IR (ATR, cm^{-1}):** ν(CN) = 2236 cm^{-1}.

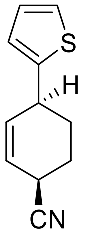

23

To a 4-dram vial was added **11D** (0.1346 g, 0.1944 mmol), $NOPF_6$ (0.0608 g, 0.3475 mmol), and acetone (1 mL). After stirring for 30 min, the reaction mix was evaporated to a film, dissolved in minimal CH_2Cl_2, and added to a 1:1 solution of hexanes and Et_2O (200 mL). A dark-brown precipitate was collected on a 30 mL F frit and washed with Et_2O (100 mL). The filtrate was evaporated to dryness, dissolved in minimal CH_2Cl_2, and purified via silica gel flash chromatography. Product isolated as light-yellow oil and desiccated overnight to give **23** (0.0212 g (57.6%)). An 11% impurity was observed in the ^{1}H NMR spectrum that is believed to be a diene (additional alkene peak a 6.12 ppm). The identity of this impurity is also supported by GCMS (m/z = 162.05), which, corresponds to loss of HCN from **23** and a molecular formula of $C_{10}H_{10}S$.

^{1}H NMR (600 MHz, d_6-acetone, δ, 25 °C): 7.31 (1H, dd, *J* = 5.1, 1.1 Hz), 6.98 (1H, dd, *J* = 5.1, 3.5 Hz), 6.91 (1H, d, *J* = 3.5 Hz), 6.05 (1H, dt, *J* = 9.8, 2.5 Hz), 5.86 (1H, m), 3.80 (1H, m), 3.53 (1H, m), 2.16 (1H, m), 2.03 (2H, overlapping), 1.87 (1H, m). **^{13}C NMR (800 MHz, d_6-acetone, δ, 25 °C):** 148.7, 134.7, 127.8, 125.1, 124.7, 123.3, 121.8, 36.7, 30.5, 26.8, 25.4. **GCMS (EI)** calculated: 189.05, observed 189.06. **IR (ATR, cm^{-1}):** ν(CN) = 2239 cm^{-1}.

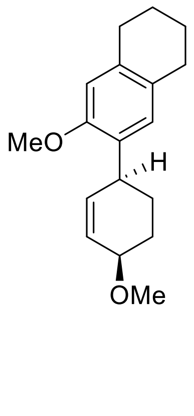

35

To a 15 mL test tube was added **34D** (0.1728 g, 0.2229 mmol) and acetone (~3 mL). This solution was chilled to -60 °C for 5 min before slowly adding a chilled (-60 °C) solution of $NOPF_6$ (0.0494 g, 0.282 mmol) in acetone (~1 mL). After 5 min, reaction removed from the glovebox, diluted with DCM (~5 mL) and washed with 20 mL of sat. Na_2CO_3 (2 x 10 mL). The aqueous layers were combined and back-extracted with DCM (~5 mL), The organic layers were combined and dried over Na_2SO_4 and evaporated onto basic alumina. The product was purified using flash chromatography on purified via silica gel flash chromatography (EtOAc:hexanes, ~80%). Yellow oil (0.0205 g (33.8%)).

^{1}H NMR (800 MHz, *d_6*-DMSO, δ, 25 °C): 6.68 (1H s), 6.63 (1H, s), 5.94 (1H, ddd, *J* = 10.1, 3.5, 2.3 Hz), 5.71 (1H, ddd, *J* = 10.1, 3.2, 1.2 Hz,), 3.73 (3H, s), 3.70 (1H, buried), 3.60 (1H, m), 3.27 (3H, s), 2.67 (2H, overlapping), 2.60 (2H, overlapping), 1.79 to 1.58 (7H, overlapping), 1.49 (1H, dtd, *J* = 13.3, 6.9, 3.5 Hz). **^{13}C NMR (800 MHz, *d_6*-DMSO, δ, 25 °C):** 154.2, 135.2, 133.3, 129.7, 128.4, 128.2, 127.7, 110.9, 72.7, 55.3, 55.3, 33.6, 28.9, 28.0, 25.8, 25.3, 23.0, 22.8. **APCI-HRMS (m/z):** [M+H] calculated for $C_{18}H_{24}O_2$ 272.1776; found 241.1588 corresponding to [M-OMe]+, $C_{17}H_{21}O$+.

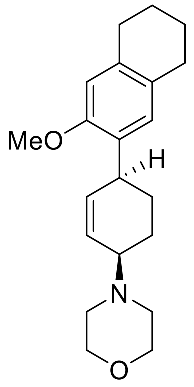

36

To a 15 mL test tube was added **33D** (0.0966 g, 0.116 mmol) and MeCN (~3 mL). This solution was chilled to -30 °C for 5 min before slowly adding a chilled (-30 °C) solution of $NOPF_6$ (0.0540 g, 0.309 mmol) in MeCN (~1 mL). After 5 min, reaction removed from the glovebox, diluted with DCM (~5 mL) and washed with 20 mL of sat. Na_2CO_3 (2 x 10 mL). The aqueous layers were combined and back-extracted with DCM (~5 mL), The organic layers were combined and dried over Na_2SO_4 and evaporated onto basic alumina. The product was purified using flash chromatography on basic alumina. Yellow oil (0.0150 g (39.4%)).

^{1}H NMR (800 MHz, *d_6*-acetone, δ, 25 °C): 6.80 (1H, s), 6.62 (1H, s), 5.88 (1H, dt, *J* = 10.4, 2.5 Hz), 5.77 (1H, dt, *J* = 10.2, 3.2 Hz), 3.78 (3H, s), 3.68 (1H, m), 3.61 (4H, m), 3.13 (1H, m), 2.71 (2H, m), 2.63 (4H, m), 2.54 (2H, m), 1.83 (1H, m), 1.74 (4H, m), 1.69 (1H, m), 1.60 (1H, m,), 1.50 (1H, m). **^{13}C NMR (800 MHz, *d_6*-acetone, δ, 25 °C) δ:** 155.8, 136.4, 133.5, 131.6, 131.2, 130.4, 128.7, 111.7, 68.2, 61.0, 55.8, 50.5, 34.5, 30.3, 29.5, 28.5, 24.4, 24.2, 20.1. **APCI-HRMS (m/z):** [M+H] calculated for $C_{21}H_{29}NO_2$ 328.2271; found 328.2270.

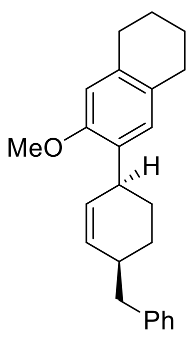

37

To a 4-dram vial was added **32D** (0.1610 g, 0.1927 mmol), acetone (~2 mL), and $NOPF_6$ (0.0556 g, 0.318 mmol). The reaction mix was removed from the glovebox and allowed to stir for approximately 30 min. The reaction mix was then evaporated to a film, dissolved in minimal CH_2Cl_2, and added to a 1:1 solution of hexanes and Et_2O (~200 mL). A dark-brown precipitate was collected on a 30 mL F frit and washed with Et_2O (100 mL). The filtrate was evaporated to dryness, dissolved in minimal CH_2Cl_2, and purified via silica gel flash chromatography (EtOAc:hexanes, ~40%). Yellow oil (0.0400 g (62.4%)).

^{1}H NMR (800 MHz, CD_2Cl_2, δ, 25 °C): 7.28 (2H, t, *J* = 7.6 Hz), 7.20 (3H, overlapping), 6.72 (1H, s), 6.54 (1H, s), 5.82 (1H, dt, *J* = 10.1, 2.6 Hz), 5.62 (1H, dt, *J* = 10.1, 3.0 Hz), 3.76 (3H, s), 3.71 (1H, m), 2.72 (3H, overlapping), 2.64 (3H, overlapping), 2.42 (1H, m), 1.82 (1H, m), 1.77 (4H, t, *J* = 3.5 Hz), 1.57 (2H, overlapping), 1.30 (1H, m,). **^{13}C NMR (800 MHz, CD_2Cl_2, δ, 25 °C):** 155.3, 141.4, 136.0, 132.9, 131.7, 130.7, 129.9, 129.8, 128.8, 128.6, 126.3, 111.2, 55.9, 42.7, 37.6, 34.3, 30.0, 29.2, 28.3, 25.7, 24.2, 23.9. **APCI-HRMS (m/z):** [M+H] calculated for $C_{24}H_{28}O$ 333.2213; found 333.2215.

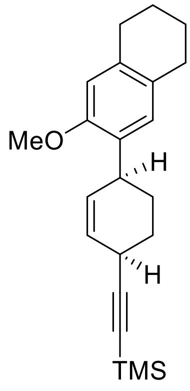

38

To a 4-dram vial was added **30D** (0.0969 g, 0.115 mmol), acetone (~2 mL), and $NOPF_6$ (0.0328 g, 0.1875 mmol). The reaction mix was removed from the glovebox and allowed to stir for approximately 30 min. The reaction mix was then evaporated to a film, dissolved in minimal CH_2Cl_2, and added to a 1:1 solution of hexanes and Et_2O (~200 mL). A dark-brown precipitate was collected on a 30 mL F frit and washed with Et_2O (100 mL). The filtrate was evaporated to dryness, dissolved in minimal CH_2Cl_2, and purified via silica gel flash chromatography (EtOAc:hexanes, ~40%). Yellow oil (0.0285 g (73.1%)).

^{1}H NMR (800 MHz, d_6-acetone, δ, 25 °C): 6.84 (1H, s), 6.56 (1H, s), 5.83 (1H, dt, J = 9.9, 3.2 Hz), 5.67 (1H, dt, J = 9.9, 2.7 Hz), 3.77 (3H, s), 3.73 (1H, m), 3.13 (1H, m), 2.73 (2H, overlapping), 2.68 (2H, overlapping), 1.85 (2H, overlapping), 1.77 (6H, overlapping), 0.16 (9H, s). **^{13}C NMR (800 MHz, d_6-acetone, δ, 25 °C):** 155.3, 136.2, 131.8, 131.2, 129.6, 129.2, 128.1, 111.3, 110.4, 84.5, 55.9, 34.2, 30.0, 29.2, 28.8, 28.1, 28.0, 24.2, 23.9, 0.5. **APCI-HRMS (m/z):** [M+H] calculated for $C_{22}H_{30}OSi$ 339.2139; found 339.2151. **IR (ATR, cm^{-1}):** ν(CC) 2165 cm^{-1}.

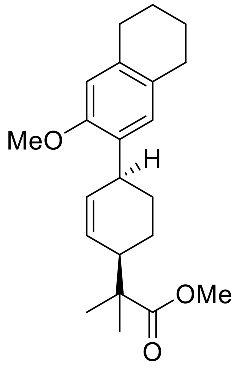

39

To a 4-dram vial was added **31D** (0.1337 g, 0.1581 mmol), acetone (~2 mL), and $NOPF_6$ (0.0411 g, 0.2349 mmol). The reaction mix was removed from the glovebox and allowed to stir for approximately 30 min. The reaction mix was then evaporated to a film, dissolved in minimal CH_2Cl_2, and added to a 1:1 solution of hexanes and Et_2O (~200 mL). A dark-brown precipitate was collected on a 30 mL F frit and washed with Et_2O (100 mL). The filtrate was evaporated to dryness, dissolved in minimal CH_2Cl_2, and purified via silica gel flash chromatography (EtOAc:hexanes, ~40%). Yellow oil (0.0320 g (59.1%)).

^{1}H NMR (800 MHz, d_6-acetone, δ, 25 °C): 6.86 (1H, s), 6.70 (1H, s), 5.88 (1H, dq, J = 10.3, 1.8 Hz), 5.79 (1H, ddd, J = 11.7, 4.3, 2.0 Hz), 3.87 (3H, s), 3.79 (1H, ddd, J = 7.0, 4.7, 2.5 Hz), 3.74 (3H, s), 2.79 (2H, overlapping), 2.71 (2H, overlapping), 2.56 (1H, m), 1.90 (1H, tdd, J = 13.1, 6.2, 2.9 Hz), 1.82 (5H, overlapping), 1.45 (1H, m), 1.33 (1H, tdd, J = 13.2, 10.7, 2.9 Hz), 1.29 (3H, s), 1.24 (3H, s). **^{13}C NMR (800 MHz, d_6-acetone, δ, 25 °C):** 178.2, 155.8, 136.3, 131.8, 131.1, 130.9, 130.5, 128.3, 111.6, 55.7, 52.0, 46.1, 44.3, 33.7, 30.1, 29.4, 29.3, 24.4, 24.2, 22.6, 22.6, 20.2. **APCI-HRMS (m/z):** [M+H] calculated for $C_{22}H_{30}O_3$ 343.2268; found 343.2263. **IR (ATR, cm^{-1}):** ν(CO) 1728 cm^{-1}.

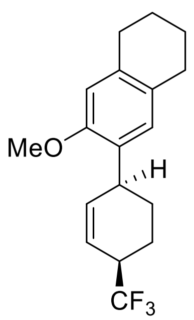

40

To a 4-dram vial was added **29D** (0.1355 g, 0.1666 mmol), acetone (~2 mL), and $NOPF_6$ (0.0427 g, 0.2440 mmol). The reaction mix was removed from the glovebox and allowed to stir for approximately 30 min. The reaction mix was then evaporated to a film, dissolved in minimal CH_2Cl_2, and added to a 1:1 solution of hexanes and Et_2O (~200 mL). A dark-brown precipitate was collected on a 30 mL F frit and washed with Et_2O (100 mL). The filtrate was evaporated to dryness, dissolved in minimal CH_2Cl_2, and purified via silica gel flash chromatography (EtOAc:hexanes, ~40%). Yellow oil (0.0320 g (59.1%)). Yellow oil (0.0320 g (62%)).

^{1}H NMR (800 MHz, *d$_6$*-acetone, δ, 25 °C): 6.75 (1H, s), 6.64 (1H, s), 5.98 (1H, dt, J = 10.2, 3.4 Hz), 5.87 (1H, dt, J = 10.2, 2.1 Hz), 3.80 (4H, s), 3.05 (1H, dp, J = 8.6, 3.1 Hz), 2.71 (2H, s), 2.63 (2H, q, J = 5.1 Hz), 1.90 (1H, dddd, J = 13.7, 10.9, 6.0, 3.0 Hz), 1.79 (1H, dtd, J = 13.3, 6.6, 3.0 Hz), 1.74 (5H, overlapping), 1.65 (1H, dddd, J = 13.6, 11.0, 8.3, 3.0 Hz). **^{13}C NMR (800 MHz, *d$_6$*-acetone, δ, 25 °C):** 155.7, 136.6, 136.2, 130.1, 130.1, 128.7, 128.7 (q, J_{CF} = 278.4 Hz), 122.3, 111.7, 55.7, 40.4 (q, J_{CF} = 26.6 Hz), 33.7, 27.4, 24.3, 24.0, 19.2 (q, J_{CF} = 2.21 Hz). **APCI-HRMS (m/z):** [M+H] calculated for $C_{18}H_{21}F_3O$ 311.1617; found 311.1621.

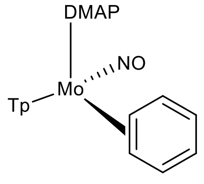

1-Mo

To a 1 L oven-dried Erlenymeyer flask was added MoTp(NO)(DMAP)(η2 -3,4-α,α,α-trifluorotoluene) (8.28 g, 0.0165 mmol) followed by anhydrous benzene (1 L, 11.2 mol) and the homogeneous red reaction mixture was allowed to stir over a period of 2.5 h. This reaction mixture was then added to 1 L of stirring hexanes in a 2 L filter flask and the resulting mixture was filtered through a fine 350 mL fine porosity fritted disc. The filtrate from this reaction was then concentrated in vacuo to ~ 1700 mL and a vibrant orange solid was isolated on a new 350 mL fine porosity fritted disc and washed with hexanes (2 x 100 mL). The solvent of the resulting filtrate was then removed in vacuo until ~ 1200 mL of solvent remained upon removal of solvent and cooling more orange solid precipitates from solution. The resulting heterogeneous orange solution was filtered through another fine 350 mL fritted disc to isolate an orange solid and this solid was washed with hexanes (2 x 100 mL) before it too was allowed to desiccate. After several hours of

desiccation under dynamic vacuum the orange solids were combined and the ^{1}H NMR signals were consistent with those previously reported for 2 (4.94 g, 55%).

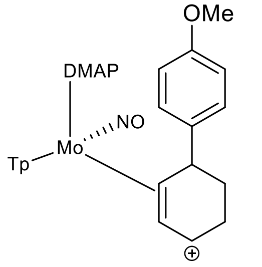

6D-Mo

To a solution of DCM (1 mL) complex **1-Mo** (0.122 g, 0.220 mmol) was added and this solution was frozen in N_2 (l). To this solution as added a recently thawed solution of HOTf (10 drops) and the reaction mixture was allowed to thaw at - 40 °C over the course of 15 min. This reaction mixture was then added to a standing solution of Et_2O (15 mL) and this solution as allowed to sit at -40 °C over a period of 16 hrs to induce precipitation. The next day a yellow solid had developed form solution. The organic layer was decanted and the solid was dried under active vacuum for 1 h before the solid was analyzed by ^{1}H NMR spectroscopy. Given the obvious signs of decomposition a mass was not taken of the isolated solid. Key ^{1}H NMR peaks of the cyclohexene ligand are given.

^{1}H NMR (800 MHz, *d_6*-acetone, δ, 25 °C): 7.24 (2H, d, J = 8.4 Hz, anisole-H3/H5) , 7.13 (2H, d, *J* = 6.8 Hz, DMAP H2/H6), 6.86 (2H, d, *J* = 8.5 Hz, anisole-H2/H6), 6.62 (1H, t, *J* = 2.0 Hz, Tp4), 6.50 (1H, t, *J* = 2.0 Hz, Tp4), 6.47 (1H, m), 6.30 (1H, t, *J* = 2.0 Hz, Tp4), 6.18 (1H, t, *J* =7.2 Hz, H2), 4.90 (1H, d, *J* = 7.0 Hz, H3), 4.05 (1H, dd, *J* = 10.1, 6.0 Hz, H4), 3.77 (3H, s, OMe).

Supplementary Fig 39. DFT Calculations for 1 (vacuum)

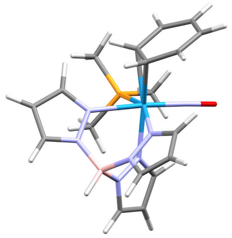

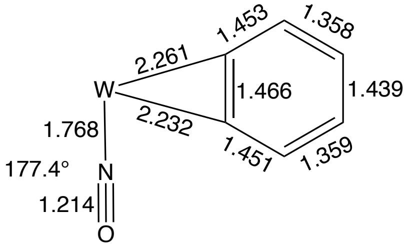

[WTp(NO)(PMe_3)(C_6H_6)] (**1**) (*reference*)

Supplementary Fig 40. DFT Calculations for 1H, 1H ---- 2, & 2 (vacuum)

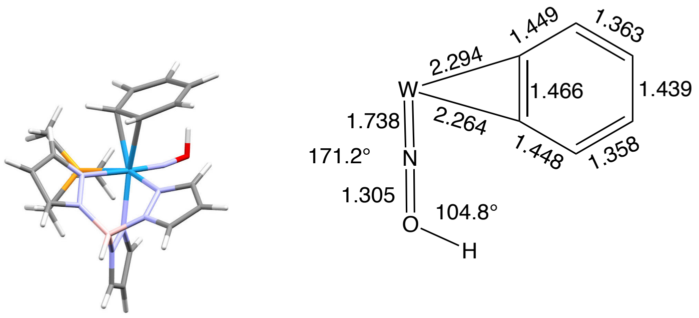

[WTp(NOH)(PMe$_3$)(C$_6$H$_6$)]$^+$ (**1H**) (E = 0) (vacuum)

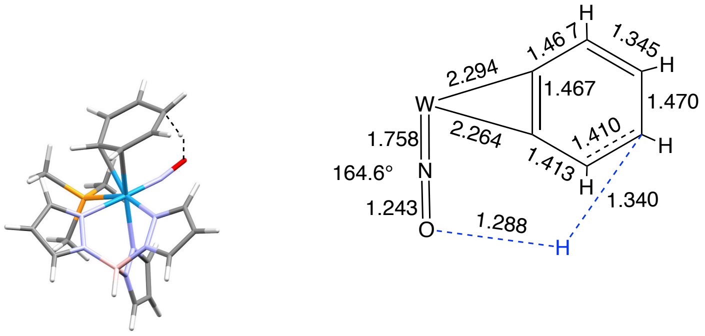

TS: **1H ---- 2** (E = 8.3 kcal/mol) (vacuum)

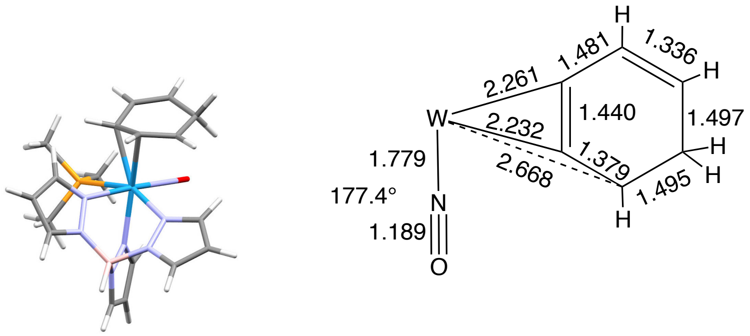

[WTp(NO)(PMe$_3$)(C$_6$H$_7$)]$^+$ (**2**) (E = -7.9 kcal/mol) (vacuum)

Supplementary Fig 41. DFT Calculations for 2H, 2H --- 5, & 5 (vacuum)

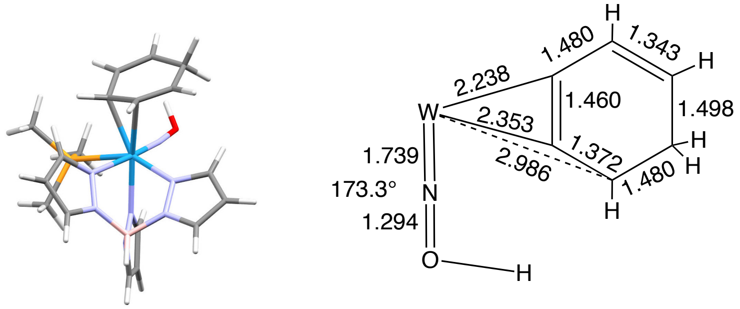

$[WTp(NOH)(PMe_3)(C_6H_7)]^{2+}$ (**2H**) (E = 0 kcal/mol) (vacuum)

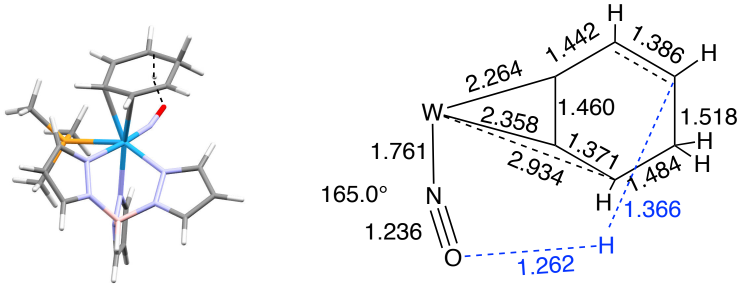

TS: **2H --- 5**) (E = 6.7 kcal/mol) (vacuum)

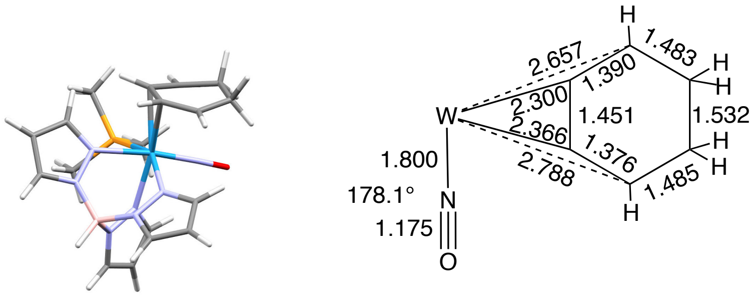

$[WTp(NOH)(PMe_3)(C_6H_7)]^{2+}$ (**5**) (E = -14.2 kcal/mol) (vacuum)

* hydrogens on bound carbons not shown.

Compound #	Structure Name	Gibbs (abs)	Gibbs (rel) kcal/mol	Gibbs (vac)	TS	(TS (vac)
**1H**	A	-1592.79908	8.7	7.9		na
**1H---2**	AG	-1592.78603	16.9	16.2	8.19	8.3
**2**	G	-1592.81301	0.0	0		na
**2H**	GP	-1593.19866	14.0	14.2		na
**2H--5**	DPTS	-1593.18887	20.1	20.9	6.14	6.7
**5**	DP	-1593.22091	0.0	0		na

Supplementary Tab 1. Calculated free energies in vacuum and in CH_2Cl_2.

Structures presented in the main text were located using the Gaussian 16 (Revision B.01) program with the M06 functional and 6-31G** basis set for all atoms except W, where the LANL2DZ basis set and pseudopotential was used. The default optimization criteria and UltraFine integration grid were used. All ground state structures were verified to ensure no imaginary frequencies, and all transition state structures were verified to have a single imaginary frequency.

All ground state structures were verified to ensure no imaginary frequencies, and all transition state structures were verified to have a single imaginary frequency. All geometry optimizations were performed using the SMD solvent model for dichloromethane and reported Gibbs energy values include the Gibbs energy of solvation.

To evaluate the feasibility of proton transfer from the nitrosyl to the bound benzene ligand, we performed DFT calculations on the relevant reactant, transition state, and product structures. Calculations indicate that the transition state is only 8 kcal/mol above the protonated nitrosyl species, suggesting that this is a viable protonation pathway.

A second proton transfer from the nitrosyl ligand to the bound benzenium ligand was also investigated, and it was found that this reaction was also fairly facile (TS barrier: 6 kcal/mol), suggesting that in either the first or the second protonation sequences, the nitrosyl ligand may serve as an aid towards proton transfer.

The default optimization criteria and UltraFine integration grid were used, except in the case of the nitrosyl-protonated benzenium complex. We encountered significantly oscillations in the energy of this structure during convergence, all within <$5*10^{-6}$ H in energy. This persisted even when we restricted the step size to 0.01 Bohr and calculated frequencies at every step. We therefore took several of these structures and performed frequency calculations on them to understand the error in the calculated Gibbs energies for these oscillating structures. We found that several of these structures display Gibbs energies within ~0.6 kcal/mol of each other, which is sufficient for the claims made in this work. We report these energies below and include these structures in our combined structure files and use the lowest energy complex for the calculated values in the maintext. We note that this provides an upper-bound estimate on the TS barrier for protonation from the nitrosyl for the structures calculated here.

Structure Trial	Absolute Energy (H)	Relative Gibbs Energy (kcal/mol)
**1**	-1593.199323	0
**2**	-1593.198655	0.59
**3**	-1593.197452	1.17

Supplementary Tab 2: Absolute energies and Gibbs energies for various protonated-nitrosyl benzenium structure. Between structures which oscillated <5*10^{-6} H in absolute energy, we found that subsequent frequency calculations yielded Gibbs energies with a standard deviation of 0.6 kcal/mol.

Supplementary Fig 42. Treatment of 1 with neat DOTf

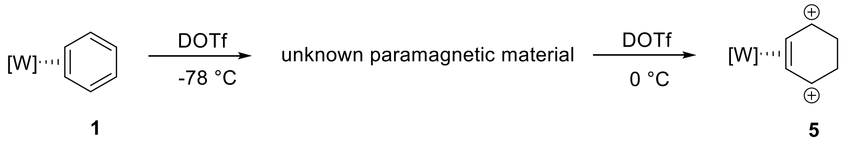

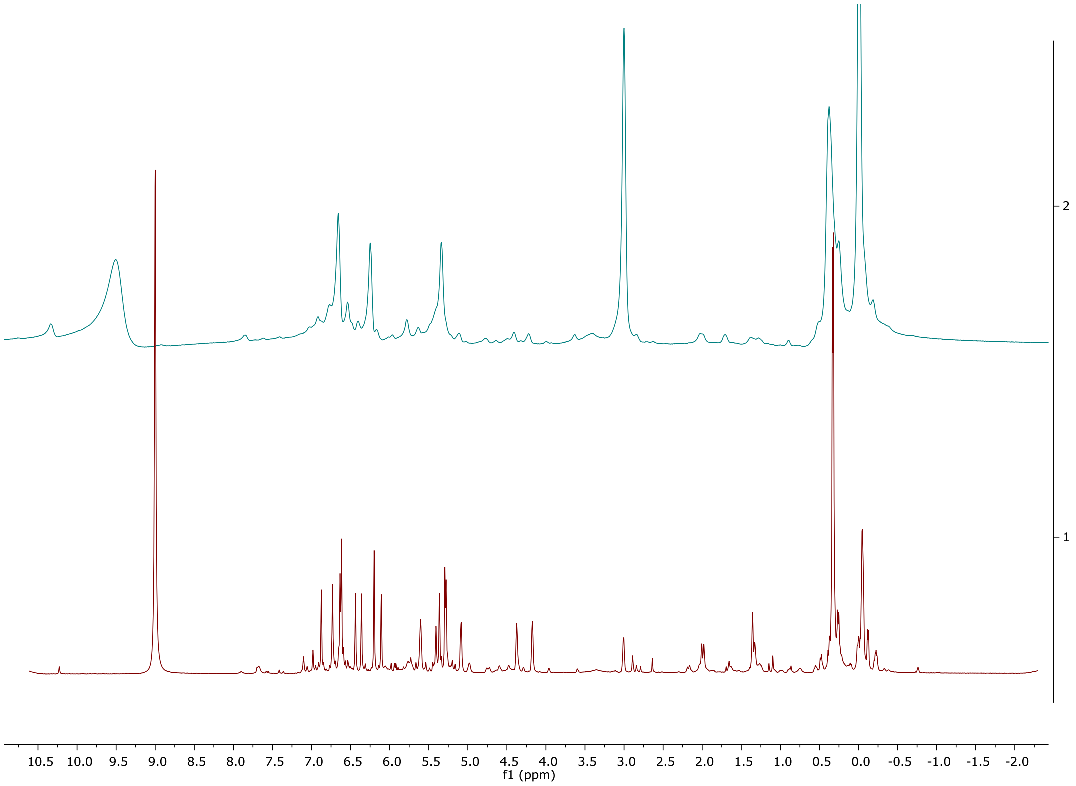

From top to bottom: Spectrum after initial treatment of **1** with neat DOTf at -78 °C. Spectrum after gradually warming to 0 °C (approximately 10 minutes).

Crystallographic Data

A single crystal of **16D, 17P, 9P, 10D, 11D, 21, 24D, 27D, 29D,** or **31D** was coated with Paratone oil and mounted on a MiTeGen MicroLoop. The X-ray intensity data for **16D** and **24D** were

measured on a Bruker Kappa APEXII Duo diffractometer. An Incoatec Microfocus IμS (Cu K_α, λ = 1.54178 Å) and a multi-layer mirror monochromator were used for **16D**. A fine-focus sealed tube (Mo K_α, λ = 0.71073 Å) and a graphite monochromator were used for **24D**. The X-ray intensity data for all other crystals were measured on a Bruker D8 Venture PhotonIII Kappa four-circle diffractometer system. An Incoatec IμS 3.0 micro-focus sealed X-ray tube (Mo Kα, λ = 0.71073 Å) and a HELIOS double bounce multilayer mirror monochromator were used for **9P**, **21, 29D,** and **31D**. An Incoatec IμS 3.0 micro-focus sealed X-ray tube (Cu Kα, λ = 1.54178 Å) and a HELIOS MX double bounce multilayer mirror monochromator were used for **17P, 10D, 11D,** and **27D**. All frames were integrated with the Bruker SAINT software package[3] using a narrow-frame algorithm. Data were corrected for absorption effects using the Multi-Scan method (SADABS).[3] Each structure was solved and refined using the Bruker SHELXTL Software Package[4] within APEX3 or APEX4 [3] and OLEX2.[5] Non-hydrogen atoms were refined anisotropically. Most O-H and B-H hydrogen atoms were located in the electron diffraction map and refined isotropically, as were H10 and H11 of **9P** and **11D**. All other hydrogen atoms were placed in geometrically calculated positions with $U_{iso} = 1.2U_{equiv}$ of the parent atom ($U_{iso} = 1.5U_{equiv}$ for methyl).

For **16D**, **10D and 31D**, solvent molecules located in the crystal lattice were severely disordered and could not be adequately modeled with or without restraints. Thus, the structure factors were modified using the PLATON SQUEEZE[6] technique, in order to produce a "solvate-free" structure factor set. PLATON reported a total electron density of 319 e^- and total solvent accessible volume of 1676 $Å^3$ for **16D**, 187 e^- and a total solvent accessible volume of 893 $Å^3$ for **10D**, and 172 e^- and total solvent accessible volume of 602 $Å^3$ for **31D**. The relative occupancy of the disordered atoms in **9P, 11D**, **21, 24D, 29D, 31D** was freely refined. Constraints and restraints were used as need on the anisotropic displacement parameters and bond lengths of the disordered atoms. For **27D**, a global RIGU restraint was used due to the weakness of the diffraction data.

Supplementary Tab 3: Crystal data for compounds **16D**, **17P**, **9P**, **10D**, **11D** and **21**.

	**16D**	**17P**	**9P**	**10D**	**11D**
CCDC number	2220172	2220173	2220174	2220175	2220176
Formula	$C_{26}H_{34}BF_3N_7O_6PSW$	$C_{24}H_{32}BF_3N_7O_5PS_2W$	$C_{27.84}H_{36.84}BCl_{0.32}N_{8.84}O_2PW$	$C_{25}H_{32}BN_8O_2PW$	$C_{23}H_{30}BN_8OPSW$
FW (g/mol)	855.29	845.31	764.27	702.21	692.24
Temp (K)	100(2)	100(2)	100(2)	100(2)	100(2)
λ (Å)	1.54178	1.54178	0.71073	1.54178	1.54178
Size (mm)	0.034 x 0.047 x 0.067	0.010 x 0.120 x 0.150	0.070 x 0.152 x 0.388	0.012 x 0.013 x 0.112	0.036 x 0.052 x 0.152
Crystal habit	yellow plate	colorless plate	colorless plate	colorless needle	colorless plate
Crystal system	monoclinic	triclinic	monoclinic	orthorhombic	monoclinic
Space group	P 2_1/c	P -1	I 2/a	P c c n	P 2_1/n

a (Å)	22.0195(5)	9.9268(6)	15.9366(5)	32.2576(18)	13.6835(6)
b(Å)	14.5709(3)	10.5902(8)	12.0562(3)	11.9426(10)	12.2825(5)
c (Å)	24.4871(5)	16.4598(16)	32.3564(13)	15.6550(9)	15.8207(7)
α (°)	90	100.543(7)	90	90	90
β (°)	102.934(2)	106.964(6)	96.4240(10)	90	95.549(3)
γ (°)	90	94.688(6)	90	90	90
Volume ($Å^3$)	7657.2(3)	1610.2(2)	6177.8(4)	6030.9(7)	2646.5(2)
Z	8	2	8	8	4
Density (g/cm^3)	1.484	1.743	1.643	1.547	1.737
μ (mm^{-1})	7.008	8.889	3.861	7.890	9.668
F(000)	3392	836	3049	2784	1368
θ range (°)	2.06 to 68.36	2.88 to 59.12	1.80 to 30.53	2.74 to 68.27	4.08 to 68.46
Index ranges	-24 ≤ h ≤ 26<br>-17 ≤ k ≤ 17<br>-29 ≤ l ≤ 26	-11 ≤ h ≤ 10<br>-11 ≤ k ≤ 11<br>-18 ≤ l ≤ 18	-22 ≤ h ≤ 22<br>-16 ≤ k ≤ 17<br>-46 ≤ l ≤ 46	-38 ≤ h ≤ 38<br>-13 ≤ k ≤ 14<br>-18 ≤ l ≤ 18	-16 ≤ h ≤ 16<br>-14 ≤ k ≤ 14<br>-16 ≤ l ≤ 19
Reflns collected	57698	20146	55469	33273	37659
Independent reflns	14046 [R_{int} = 0.0847]	4627 [R_{int} = 0.1572]	9439 [R_{int} = 0.0487]	5518 [R_{int} = 0.1549]	4852 [R_{int} = 0.0923]
Data / restraints /parameters	14046 / 2 / 843	4627 / 0 / 404	9439 / 2 / 418	5518 / 1 / 350	4852 / 156 / 386
GOF on F^2	1.019	1.026	1.049	0.996	1.068
R_1 (I>2σ(I))	0.0439	0.0929	0.0275	0.0622	0.0478
wR_2 (all data)	0.1109	0.2617	0.0610	0.1869	0.1343

Supplementary Tab 4. Crystallographic data for **24D**, **27D**, **29D**, **31D**

	**21**	**24D**	**27D**	**29D**	**31D**
CCDC number	2220177	2254578	2254579	2254580	2254581
Formula	$C_{13}H_{13}NO$	$C_{35}H_{50}BF_3N_7O_7PSW$	$C_{35}H_{48}BF_3N_7O_6PSW$	$C_{30}H_{40}BF_3N_7O_2PW$	$C_{34}H_{49}BN_7O_4PW$
FW (g/mol)	199.24	995.51	977.49	813.32	845.43
Temp (K)	100(2)	100(2)	100(2)	100(2)	100(2)
λ (Å)	0.71073	0.71073	1.54178	0.71073	0.71073
Size (mm)	0.194 x 0.550 x 0.567	0.106 x 0.119 x 0.124	0.029 x 0.034 x 0.066	0.045 x 0.060 x 0.132	0.103 x 0.144 x 0.221
Crystal habit	colorless block	yellow block	blue-green plate	pale yellow needle	colorless block
Crystal system	monoclinic	monoclinic	monoclinic	orthorhombic	monoclinic
Space group	P 2_1/c	P 2_1/n	P 2_1	P $2_12_12_1$	P 2_1/c
a (Å)	10.9372(4)	10.5853(8)	10.8303(5)	8.7222(4)	15.9401(5)
b(Å)	8.9098(3)	13.7626(12)	14.1402(7)	15.2574(6)	13.3600(4)
c (Å)	11.4878(4)	26.901(2)	13.0345(7)	24.0871(8)	19.6480(6)
α (°)	90	90	90	90	90
β (°)	107.7370(10)	95.804(2)	95.463(4)	90	104.7470(10)
γ (°)	90	90	90	90	90
Volume ($Å^3$)	1066.25(6)	3898.9(5)	1987.07(17)	3205.5(2)	4046.4(2)
Z	4	4	2	4	4
Density (g/cm^3)	1.241	1.696	1.634	1.685	1.388
μ (mm^{-1})	0.079	3.129	6.834	3.712	2.936
F(000)	424	2008	984	1624	1712
θ range (°)	2.95 to 28.27	1.52 to 28.34	3.41 to 66.95	2.15 to 28.29	2.02 to 29.58
Index ranges	-14 ≤ h ≤ 14<br>-9 ≤ k ≤ 11<br>-15 ≤ l ≤ 15	-13 ≤ h ≤ 14<br>-18 ≤ k ≤ 18<br>-35 ≤ l ≤ 35	-12 ≤ h ≤ 12<br>-16 ≤ k ≤ 16<br>-15 ≤ l ≤ 14	-11 ≤ h ≤ 9<br>-20 ≤ k ≤ 20<br>-27 ≤ l ≤ 32	-22 ≤ h ≤ 22<br>-17 ≤ k ≤ 18<br>-26 ≤ l ≤ 27
Reflns collected	17388	46211	18353	40453	79390
Independent reflns	2644 [R_{int} = 0.0342]	9721 [R_{int} = 0.0795]	6923 [R_{int} = 0.1443]	7954 [R_{int} = 0.0939]	11346 [R_{int} = 0.0480]
Data / restraints /parameters	2644 / 5 / 156	9721 / 0 / 575	6923 / 467 / 504	7954 / 2 / 420	11346 / 168 / 495

GOF on F^2	1.089	1.029	1.067	1.057	1.013
R_1 ($I>2\sigma(I)$)	0.0423	0.0397	0.0739	0.0420	0.0240
wR_2 (all data)	0.1172	0.0843	0.1748	0.0639	0.0487

References

(1) Welch, K. D.; Harrison, D. P.; Lis, E. C.; Liu, W.; Salomon, R. J.; Harman, W. D.; Myers, W. H. Large-Scale Syntheses of Several Synthons to the Dearomatization Agent {TpW(NO)(PMe3)} and Convenient Spectroscopic Tools for Product Analysis. *Organometallics* **2007**, *26* (10), 2791–2794. https://doi.org/10.1021/om070034g.

(2) Keane, J. M.; Ding, F.; Sabat, M.; Harman, W. D. Solid-State Induced Control of Kinetically Unstable Stereoisomers. *J. Am. Chem. Soc.* **2004**, *126* (3), 785–789. https://doi.org/10.1021/ja0305860.

(3) Bruker (2012). *Saint; SADABS; APEX3; APEX4*. Bruker AXS Inc., Madison, Wisconsin, USA.

(4) Sheldrick, G. M. (2015). *Acta Cryst.* A**71**, 3-8.

(5) Dolomanov, O. V.; Bourhis, L. J.; Gildea, R. J.; Howard, J. A. K.; Puschmann, H. *J. Appl. Cryst.* (2009). **42**, 339-341.

(6) Spek, A. L. *Acta Crystallogr. Sect C: Struct. Chem.* **2015**, *C71*, 9-18.
