# Supplementary material for: The double protonation of dihapto-coordinated benzene complexes enables dearomatization using aromatic nucleophiles
Source: Nat Commun. 2023 May 30;14:3145. doi: 10.1038/s41467-023-38945-0 (PMC10229636; doi:10.1038/s41467-023-38945-0)
Supplement: Supplementary file 3 — Description of Additional Supplementary Files [file 41467_2023_38945_MOESM3_ESM.pdf]

Description of Additional Supplementary Files

File Name: Supplementary Data 1
Description: combined cif file containing all structured solved by single crystal XRD

File Name: Supplementary Data 2
Description: Combined list of coordinates for optimized structures
