# Supplementary material for: The double protonation of dihapto-coordinated benzene complexes enables dearomatization using aromatic nucleophiles
Source: Nat Commun. 2023 May 30;14:3145. doi: 10.1038/s41467-023-38945-0 (PMC10229636; doi:10.1038/s41467-023-38945-0)
Supplement: Supplementary file 5 — Supplementary Data 2 [file 41467_2023_38945_MOESM5_ESM.doc]

55

A DCM

W -0.37180 0.15270 -0.14030

P -1.02920 -2.30900 -0.10880

N 0.89160 -0.45680 1.64430

N 2.24410 -0.33600 1.57950

N 2.60270 -0.53770 -0.89720

N 1.31910 -0.61270 -1.32130

N 2.35730 1.70310 0.12060

N 1.03340 1.87450 -0.12400

N -1.39080 0.55700 -1.48750

C 0.61500 -0.99010 2.84470

H -0.40950 -1.17810 3.14180

C 1.78670 -1.22370 3.55830

H 1.88610 -1.64070 4.55010

C 2.79630 -0.79090 2.71470

H 3.87120 -0.76860 2.84350

C 2.64800 -1.47240 -2.90760

H 2.98870 -1.92760 -3.82650

C 3.41720 -1.04940 -1.83630

H 4.48790 -1.06540 -1.67610

C 0.86100 3.15490 -0.46850

H -0.12720 3.51870 -0.72300

C 2.08200 3.83110 -0.43930

H 2.26650 4.87250 -0.66150

C 3.00240 2.87070 -0.06070

H 4.07240 2.92950 0.09520

C 0.41620 -3.43000 -0.14030

H 0.07170 -4.46960 -0.09300

H 1.00490 -3.28880 -1.05190

C -2.00240 -3.04100 1.25600

H -1.45700 -2.96620 2.20270

H -2.97860 -2.55900 1.36440

C -2.00960 -2.76970 -1.57690

H -2.20860 -3.84780 -1.57610

H -2.96240 -2.22770 -1.55890

B 2.94380 0.29500 0.36000

H 4.12840 0.34400 0.53110

C -2.15070 0.23550 1.29510

H -2.00830 -0.49210 2.09370

C -1.43620 1.49630 1.33670

H -0.69700 1.64580 2.12610

C -2.10360 2.68670 0.84970

H -1.63150 3.65220 1.02360

C -3.99810 1.35890 0.12580

C -3.45500 0.22930 0.66130

H -4.02650 -0.69910 0.65580

C 1.33760 -1.17000 -2.54020

H 0.41240 -1.32610 -3.08250

H -2.16040 -4.10250 1.03090

H 1.05610 -3.22600 0.72710

H -1.47520 -2.50210 -2.49420

C -3.31630 2.62080 0.24180

H -4.99590 1.32750 -0.30960

H -3.80710 3.52500 -0.11100

O -2.28680 0.83700 -2.39950

H -3.12070 1.01320 -1.89800

55

AG DCM

O -2.12300 1.58040 -2.07690

N 1.14050 1.82950 -0.01600

N 2.44580 1.55340 0.23590

N 0.86370 -0.58740 1.64760

N 2.21980 -0.56490 1.56130

N 1.24650 -0.58760 -1.35040

N 2.53590 -0.62510 -0.93490

N -1.32690 0.88800 -1.40430

C 3.17390 2.67900 0.12100

C 2.32990 3.72160 -0.21670

C 1.06680 3.13530 -0.29850

C 2.75940 -1.13440 2.65100

C 1.73610 -1.54720 3.48640

C 0.57200 -1.17940 2.81630

C 3.31450 -1.06920 -1.93590

C 2.51700 -1.33260 -3.03820

C 1.22770 -1.00450 -2.62420

B 2.94000 0.09490 0.37510

H 4.12780 0.05850 0.52680

H 4.24380 2.65240 0.28600

H 2.59090 4.75680 -0.38380

H 0.11670 3.58430 -0.56260

H 3.83520 -1.19990 2.75470

H 1.82100 -2.03730 4.44560

H -0.45590 -1.31360 3.13040

H 4.38480 -1.15380 -1.79480

H 2.82760 -1.70150 -4.00520

H 0.29090 -1.05100 -3.16740

C 0.26230 -3.41810 -0.51420

H -0.13350 -4.44040 -0.52050

H 0.81020 -3.24190 -1.44420

H 0.95520 -3.30860 0.32950

C -2.18690 -2.50540 -1.77180

H -2.47690 -3.56020 -1.84560

H -3.08510 -1.88440 -1.67680

H -1.65620 -2.21180 -2.68350

W -0.37100 0.19920 -0.10240

P -1.12270 -2.23910 -0.31560

C -2.05350 -3.05470 1.02900

H -2.25020 -4.09080 0.72930

H -1.46070 -3.06590 1.94940

H -3.00910 -2.55870 1.21920

C -3.99840 1.25070 0.21260

C -3.47350 0.15060 0.78470

C -2.10650 0.12170 1.31320

C -1.43260 1.41470 1.47890

C -2.01990 2.54350 0.86300

C -3.19640 2.47650 0.09190

H -4.08690 -0.74280 0.89780

H -1.94810 -0.60360 2.11010

H -0.72390 1.58350 2.28930

H -1.54530 3.51510 0.99960

H -3.71370 3.42090 -0.08780

H -5.01200 1.25210 -0.18050

H -2.69150 2.24030 -1.13310

56

DP DCM

W -0.41760 0.25180 0.00960

P -1.16800 -2.19540 -0.46050

N 0.80000 -0.73560 1.62670

N 2.15420 -0.77960 1.52340

N 2.38910 -0.69170 -0.97400

N 1.09740 -0.50900 -1.35190

N 2.47120 1.39790 0.31410

N 1.18500 1.74170 0.03700

N -1.24040 0.96560 -1.41040

C 0.49230 -1.38090 2.76700

H -0.53320 -1.48530 3.09650

C 1.64230 -1.84660 3.39370

H 1.71220 -2.39630 4.32110

C 2.67600 -1.43910 2.56790

H 3.74810 -1.56620 2.65040

C 2.28520 -1.10520 -3.15420

H 2.55290 -1.36320 -4.16860

C 3.11560 -1.04380 -2.04620

H 4.18100 -1.21060 -1.94650

C 1.19180 3.03010 -0.34060

H 0.27210 3.52250 -0.63740

C 2.48490 3.53820 -0.28130

H 2.80940 4.54340 -0.50820

C 3.26290 2.46620 0.12410

H 4.33080 2.38270 0.28290

C 0.21930 -3.35110 -0.68620

H -0.19610 -4.36340 -0.75360

H 0.77830 -3.13680 -1.60060

C -2.12080 -3.04460 0.84270

H -1.50800 -3.14830 1.74400

H -3.06180 -2.54920 1.09430

C -2.19700 -2.33690 -1.95360

H -2.54960 -3.36930 -2.05910

H -3.05580 -1.65990 -1.87780

B 2.88570 -0.09530 0.36790

H 4.07050 -0.21030 0.47530

C -2.11580 0.13860 1.55490

H -2.03400 -0.76480 2.15280

C -1.35670 1.33630 1.86720

H -0.60860 1.33360 2.65630

C -1.60980 2.48710 1.15550

H -0.94510 3.33540 1.31670

C -3.06640 0.21940 0.54590

H -3.57780 -0.69250 0.23570

C 1.03170 -0.74410 -2.67410

H 0.09200 -0.65390 -3.20620

H -2.35430 -4.04840 0.46750

H 0.89260 -3.29890 0.17740

H -1.61420 -2.06720 -2.84010

O -1.74290 1.43180 -2.37290

C -2.80660 2.73090 0.31860

H -2.51030 3.23580 -0.61080

H -3.39420 3.48610 0.86190

C -3.63930 1.48160 0.02990

H -3.86350 1.38360 -1.03960

H -4.62860 1.53550 0.51230

55

G DCM

W -0.37630 0.28470 -0.02260

P -1.30000 -2.08400 -0.38090

N 0.79990 -0.70670 1.64260

N 2.14760 -0.82110 1.54300

N 2.40720 -0.79920 -0.96040

N 1.12780 -0.58260 -1.35510

N 2.56360 1.32410 0.28950

N 1.29710 1.75040 0.03250

N -1.24870 0.89890 -1.44030

C 0.44980 -1.33310 2.77590

H -0.58690 -1.37160 3.08860

C 1.57240 -1.86080 3.41130

H 1.60890 -2.41360 4.33910

C 2.63050 -1.50880 2.59120

H 3.69390 -1.69340 2.67910

C 2.29970 -1.32450 -3.11460

H 2.56230 -1.64510 -4.11260

C 3.12690 -1.24010 -2.00630

H 4.18340 -1.44770 -1.88970

C 1.38360 3.04750 -0.29170

H 0.49600 3.60540 -0.56630

C 2.70750 3.47790 -0.22790

H 3.09100 4.46910 -0.42320

C 3.42040 2.34930 0.13600

H 4.48110 2.19660 0.29160

C 0.00750 -3.34490 -0.59840

H -0.46310 -4.33030 -0.69460

H 0.61200 -3.15180 -1.48890

C -2.32830 -2.90110 0.89080

H -1.79900 -2.94370 1.84830

H -3.28540 -2.38980 1.02630

C -2.34340 -2.22170 -1.86950

H -2.70500 -3.25120 -1.97680

H -3.19990 -1.54480 -1.77300

B 2.90710 -0.18570 0.37250

H 4.08810 -0.34490 0.49430

C -2.19100 0.19390 1.35800

H -2.19980 -0.63340 2.06520

C -1.41410 1.34660 1.74330

H -0.67690 1.30810 2.54400

C -1.54850 2.49070 0.98780

H -0.85350 3.31000 1.16380

C -3.76150 1.67860 0.16640

C -3.48390 0.48230 0.69640

H -4.22780 -0.31390 0.66760

C 1.05820 -0.88620 -2.65880

H 0.12330 -0.77530 -3.19570

H -2.52690 -3.92620 0.55610

H 0.66120 -3.34710 0.28250

H -1.78050 -1.94610 -2.76670

H -4.72690 1.87290 -0.29600

O -1.83440 1.27010 -2.41490

C -2.76220 2.79270 0.17360

H -2.48800 3.07870 -0.85520

H -3.20310 3.71260 0.59430

56

GP DCM

W -0.37650 0.22730 -0.08620

P -0.93460 -2.30360 -0.14520

N 0.87650 -0.47190 1.64290

N 2.23310 -0.45030 1.53360

N 2.49490 -0.59030 -0.96240

N 1.19400 -0.57010 -1.34870

N 2.40720 1.61530 0.13740

N 1.08570 1.85420 -0.07240

N -1.37440 0.59610 -1.45860

C 0.60160 -0.99700 2.85120

H -0.42020 -1.11330 3.18960

C 1.77530 -1.32130 3.51940

H 1.87670 -1.75300 4.50450

C 2.78610 -0.95420 2.64570

H 3.86340 -1.00930 2.73940

C 2.42770 -1.30890 -3.06280

H 2.71880 -1.67810 -4.03550

C 3.24970 -1.02840 -1.98210

H 4.32460 -1.09430 -1.87000

C 0.96370 3.15480 -0.38560

H -0.00820 3.57520 -0.61600

C 2.21030 3.76890 -0.36350

H 2.43900 4.80620 -0.56090

C 3.09480 2.75470 -0.03040

H 4.16970 2.76260 0.10020

C 0.55190 -3.35220 -0.23790

H 0.22890 -4.39890 -0.19520

H 1.10330 -3.19000 -1.16790

C -1.84550 -3.07310 1.23220

H -1.31490 -2.93400 2.17950

H -2.86360 -2.68180 1.31710

C -1.94100 -2.73700 -1.59900

H -2.11450 -3.81950 -1.60370

H -2.90610 -2.22170 -1.54250

B 2.93110 0.16940 0.31360

H 4.11900 0.14940 0.43850

C -2.13380 0.07120 1.27830

H -2.05890 -0.68490 2.05770

C -1.54190 1.36830 1.59850

H -0.77400 1.46120 2.36660

C -2.01990 2.51160 1.00680

H -1.52950 3.46140 1.22100

C -3.95070 1.23970 0.07250

C -3.43860 0.11090 0.58410

H -3.99830 -0.82160 0.52070

C 1.14720 -0.99290 -2.62380

H 0.20130 -1.05030 -3.14910

H -1.90310 -4.14700 1.01860

H 1.20700 -3.14770 0.61660

H -1.43630 -2.45160 -2.52720

H -4.93080 1.23790 -0.39980

C -3.24050 2.54950 0.17990

H -3.00700 2.98370 -0.80830

H -3.89570 3.31930 0.62460

O -2.17360 0.79170 -2.45860

H -3.07820 0.52490 -2.16070

56

GP 2 DCM

W -0.3796 0.2302 -0.0899

P -0.9209 -2.3080 -0.1382

N 0.8720 -0.4615 1.6450

N 2.2289 -0.4506 1.5327

N 2.4921 -0.5939 -0.9645

N 1.1913 -0.5682 -1.3502

N 2.4112 1.6107 0.1339

N 1.0903 1.8540 -0.0741

N -1.3834 0.5869 -1.4623

C 0.5964 -0.9726 2.8592

H -0.4248 -1.0753 3.2037

C 1.7692 -1.2983 3.5283

H 1.8694 -1.7201 4.5178

C 2.7807 -0.9468 2.6490

H 3.8577 -1.0074 2.7417

C 2.4221 -1.2993 -3.0696

H 2.7119 -1.6641 -4.0443

C 3.2453 -1.0273 -1.9876

H 4.3201 -1.0966 -1.8764

C 0.9718 3.1566 -0.3809

H 0.0007 3.5799 -0.6094

C 2.2201 3.7670 -0.3572

H 2.4518 4.8044 -0.5508

C 3.1019 2.7488 -0.0289

H 4.1767 2.7531 0.1014

C 0.5734 -3.3469 -0.2120

H 0.2538 -4.3947 -0.1696

H 1.1331 -3.1861 -1.1371

C -1.8292 -3.0696 1.2454

H -1.3047 -2.9093 2.1929

H -2.8523 -2.6898 1.3206

C -1.9099 -2.7782 -1.5939

H -2.0737 -3.8621 -1.5741

H -2.8801 -2.2726 -1.5662

B 2.9302 0.1634 0.3118

H 4.1179 0.1400 0.4378

C -2.1351 0.0766 1.2806

H -2.0534 -0.6742 2.0641

C -1.5477 1.3787 1.5887

H -0.7787 1.4819 2.3545

C -2.0315 2.5142 0.9864

H -1.5445 3.4679 1.1908

C -3.9628 1.2281 0.0749

C -3.4459 0.1068 0.5973

H -4.0056 -0.8266 0.5498

C 1.1425 -0.9827 -2.6277

H 0.1941 -1.0358 -3.1484

H -1.8725 -4.1471 1.0475

H 1.2183 -3.1393 0.6492

H -1.3921 -2.5137 -2.5223

H -4.9453 1.2188 -0.3924

C -3.2528 2.5392 0.1600

H -3.0190 2.9556 -0.8359

H -3.9073 3.3169 0.5914

O -2.1863 0.7736 -2.4615

H -3.0811 0.4654 -2.1755

56

GP 3 DCM

W -0.3764 0.2303 -0.0871

P -0.9340 -2.2996 -0.1397

N 0.8751 -0.4682 1.6435

N 2.2318 -0.4560 1.5309

N 2.4915 -0.5987 -0.9662

N 1.1903 -0.5716 -1.3511

N 2.4141 1.6070 0.1337

N 1.0933 1.8521 -0.0734

N -1.3786 0.5922 -1.4588

C 0.5999 -0.9857 2.8551

H -0.4212 -1.0911 3.1988

C 1.7731 -1.3150 3.5218

H 1.8740 -1.7425 4.5088

C 2.7842 -0.9586 2.6440

H 3.8613 -1.0199 2.7356

C 2.4191 -1.3106 -3.0686

H 2.7077 -1.6788 -4.0425

C 3.2433 -1.0368 -1.9881

H 4.3181 -1.1080 -1.8772

C 0.9762 3.1544 -0.3815

H 0.0054 3.5791 -0.6091

C 2.2254 3.7633 -0.3586

H 2.4581 4.8004 -0.5525

C 3.1061 2.7443 -0.0303

H 4.1809 2.7475 0.0997

C 0.5497 -3.3517 -0.2405

H 0.2233 -4.3971 -0.1925

H 1.0956 -3.1949 -1.1745

C -1.8345 -3.0666 1.2458

H -1.2947 -2.9268 2.1878

H -2.8514 -2.6744 1.3402

C -1.9504 -2.7344 -1.5859

H -2.1217 -3.8172 -1.5890

H -2.9163 -2.2215 -1.5224

B 2.9318 0.1589 0.3096

H 4.1196 0.1340 0.4339

C -2.1319 0.0770 1.2807

H -2.0545 -0.6755 2.0633

C -1.5418 1.3765 1.5942

H -0.7734 1.4745 2.3612

C -2.0218 2.5158 0.9963

H -1.5322 3.4674 1.2044

C -3.9531 1.2360 0.0728

C -3.4382 0.1108 0.5894

H -3.9968 -0.8229 0.5323

C 1.1402 -0.9903 -2.6274

H 0.1934 -1.0429 -3.1515

H -1.8950 -4.1406 1.0341

H 1.2109 -3.1475 0.6094

H -1.4518 -2.4488 -2.5175

H -4.9339 1.2303 -0.3981

C -3.2435 2.5470 0.1706

H -3.0114 2.9745 -0.8209

H -3.8987 3.3194 0.6108

O -2.1806 0.7801 -2.4582

H -3.0799 0.4928 -2.1637
